# Antibody Persistence and Booster Responses to Split-Virion H5N1 Avian Influenza Vaccine in Young and Elderly Adults

**DOI:** 10.1371/journal.pone.0165384

**Published:** 2016-11-04

**Authors:** Rajeka Lazarus, Sarah Kelly, Matthew D. Snape, Corinne Vandermeulen, Merryn Voysey, Karel Hoppenbrouwers, Annick Hens, Pierre Van Damme, Stephanie Pepin, Isabel Leroux-Roels, Geert Leroux-Roels, Andrew J. Pollard

**Affiliations:** 1 Oxford Vaccine Group, Department of Paediatrics, Centre for Clinical Vaccinology and Tropical Medicine, University of Oxford and the NIHR Oxford Biomedical Research Centre, Oxford, United Kingdom; 2 Center for Environment and Health, Leuven University, Leuven, Belgium; 3 Vaccinology Center, Leuven University, Leuven, Belgium; 4 Nuffield Department of Primary Care Health Sciences, University of Oxford, Oxford, United Kingdom; 5 Centre for the Evaluation of Vaccination, Vaccine & Infectious Disease Institute, University of Antwerp, Antwerp, Belgium; 6 Sanofi Pasteur, Marcy l’Etoile, France; 7 Center for Vaccinology, Ghent University and Ghent University Hospital, Ghent, Belgium; Public Health England, UNITED KINGDOM

## Abstract

Avian influenza continues to circulate and remains a global health threat not least because of the associated high mortality. In this study antibody persistence, booster vaccine response and cross-clade immune response between two influenza A(H5N1) vaccines were compared. Participants aged over 18-years who had previously been immunized with a clade 1, A/Vietnam vaccine were re-immunized at 6-months with 7.5 μg of the homologous strain or at 22-months with a clade 2, alum-adjuvanted, A/Indonesia vaccine. Blood sampled at 6, 15 and 22-months after the primary course was used to assess antibody persistence. Antibody concentrations 6-months after primary immunisation with either A/Vietnam vaccine 30 μg alum-adjuvanted vaccine or 7.5 μg dose vaccine were lower than 21-days after the primary course and waned further with time. Re-immunization with the clade 2, 30 μg alum-adjuvanted vaccine confirmed cross-clade reactogenicity. Antibody cross-reactivity between A(H5N1) clades suggests that in principle a prime-boost vaccination strategy may provide both early protection at the start of a pandemic and improved antibody responses to specific vaccination once available.

***Trial Registration*:** ClinicalTrials.gov NCT00415129

## Introduction

The first influenza pandemic of the 21st century in 2009 was caused by a novel influenza A(H1N1) strain that was first recognized in Mexico [1] and not by the A(H5N1) strain as was anticipated. However, the threat posed by avian influenza viruses, including the A(H5N1) viruses, persists.

The A(H5N1) virus is enzootic in some parts of Africa and Asia resulting in regular outbreaks in poultry and wild birds. Human cases of A(H5N1) peaked in 2006 but new cases continue to be diagnosed and a total of 844 confirmed infections has been reported to the World Health Organization (WHO) to date [2]. Whereas the pandemic A(H1N1) 2009 influenza strain had a mortality very similar to that of seasonal influenza, the mortality associated with A(H5N1) and A(H7N9) avian viruses is approximately 60% and 30%, respectively [2]. The higher mortality rate associated with avian influenza is in part due to the lack of pre-existing immunity against avian derived influenza viruses in the human population. This lack of pre-existing immunity also explains the poor antibody responses to A(H5N1) vaccines.

Sporadic transmission of (H5N1) influenza virus amongst close household contacts has been observed but sustained human-to-human transmission has not yet been reported [3]. Five key amino acid gene mutations that have been demonstrated to occur when the virus is passaged through ferrets suffice to make the virus more transmissible. Therefore continued vigilance is warranted and preparedness plans need to be maintained [4]. The 2009 A(H1N1) influenza outbreak uncovered the shortcomings of existing preparedness plans, more specifically the inability of the community as a whole to respond quickly to the emergence of a new pandemic and the incapacity to develop, manufacture and deliver an effective vaccine to the target population in time. Two major challenges in designing and implementing a A(H5N1) pandemic vaccine strategy are anticipating antigenic variants as a result of antigenic drift and overcoming the weak immunogenicity due to the lack of pre-existing immunity. Both challenges may be tackled by using a pre-pandemic vaccine to prime the population prior to a pandemic. This strategy is based on two assumptions: first, that priming of a population with a pre-pandemic vaccine will induce and maintain cross-reactive antibodies that will convey protection against the pandemic virus before the pandemic strain-specific vaccine becomes available, and second that boosting with a strain-matched pandemic vaccine will produce faster, higher and more cross-protective antibody responses in a primed compared to an unprimed population [5–7].

In this study, antibody persistence, booster response and cross-clade responses in adults who had been previously vaccinated with two doses of a clade 1 A(H5N1) high dose alum-adjuvanted or unadjuvanted low dose vaccine were evaluated after re-immunization with an unadjuvanted low dose vaccine containing the original vaccine strain or a high dose alum-adjuvanted clade 2 strain.

## Methods

A booster immunization was given in an open-label, phase 2 study. The primary phase was conducted between May and December 2006, and has previously been reported [8]. This secondary phase was conducted between December 2006 and October 2008.

The primary study was conducted at 4 study sites in Europe, whereas the booster study conducted in 3 out of these 4 sites.

### Study Design

In the previously reported randomized, open-label, uncontrolled phase 2 trial, 600 adults (divided equally between two age groups: 18 to 60 years and over 60 years) were randomized to receive 2 doses (D0, D21) of H5N1 clade 1 vaccine containing either 7.5 μg haemagglutinin without adjuvant or, 30 μg with 600 μg aluminum hydroxide [Al3+] as adjuvant [8].

**Fig 1:**
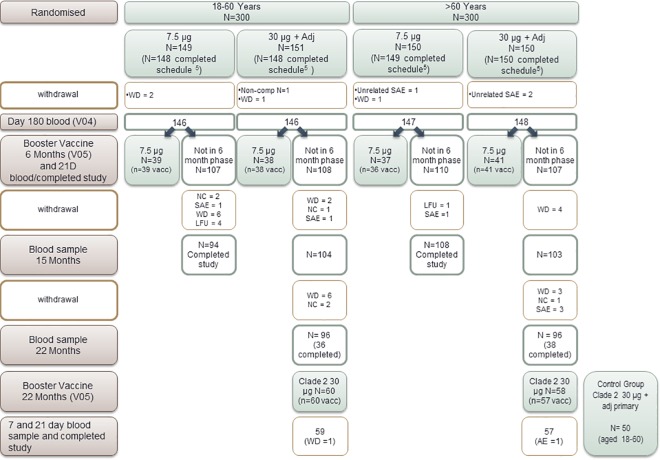
Study CONSORT diagram. NC: non compliance with protocol; SAE: serious adverse event; WD: voluntary withdrawal for unspecified reasons; LFU: lost to follow up.

The booster study, presented here, was conducted in 2 phases. A cohort of participants at one study site (n = 154) received a booster dose of the 7.5 μg (H5N1) clade 1 vaccine, 6 months after having received the primary immunization course with the 7.5 μg (H5N1) clade 1 vaccine, either without adjuvant or with 30 μg alum. At a later stage and in 2 of the 3 remaining sites, subjects who had been primed with two doses of the 30 μg alum-adjuvanted vaccine, were invited to participate at the booster study which implied that they received one booster immunization with a high dose (30 μg) (H5N1) clade 2 alum-adjuvanted A(H5N1) vaccine at month 22 after the primary immunization (Fig 1). Further details about the study design are available in S1 File clinical trial protocol.

In order to assess antibody persistence all participants were invited to undergo a blood test at 6 months after starting the study. Thereafter, all participants who did not receive a booster vaccine at 6 months were invited to have a blood test to assess antibody persistence at 15 and 22 months.

In one study site, parallel to the 22-month clade 2 booster immunization an additional 50 A(H5N1) vaccine-naïve adults, aged between 18 and 60 years, were recruited to receive a primary course of the same clade 2 vaccine that was used as booster. These participants received 2 doses of the vaccine given three weeks apart.

### Study Participants

Adults who had previously participated in the primary study were invited to take part in the follow-up and booster phase [8]. The following exclusion criteria were used for all participants who took part in the booster phase of the study: systemic hypersensitivity to any component of a vaccine or life-threatening reaction after previous vaccines; congenital or acquired immunodeficiency or receipt of immunosuppressive therapy such as anti-cancer chemotherapy or long-term systemic corticosteroid therapy (for more than 2 consecutive weeks in the last 3 months before the visit); receipt of blood products within the past 3 months; vaccination within 4 weeks prior to the trial vaccination or vaccination planned within 4 weeks after any trial vaccination; pregnancy (confirmed by positive urine test) or a serious adverse event related to the trial vaccine following vaccination. For the 50 naive participants, recruited to receive clade 2 as a primary vaccination, the following additional exclusion criteria were applied: chronic illness that could have interfered with the trial conduct or completion; previous vaccination with avian influenza vaccine; planned or recent participation in another clinical trial; bleeding disorder that prohibited intra-muscular injections or a febrile illness on the day of inclusion.

The study was approved by the institutional ethics review boards based in each participating Belgium institution (Universities of Antwerp, Ghent and Leuven) and the National Health Service Oxford ethics committee for the Oxford site., The study was conducted in accordance with the Declaration of Helsinki and Good Clinical Practice. Written informed consent was obtained from all participants before enrolment in this part of the study. The trial is registered with Clinicaltrials.gov: NCT00415129.

### Study Procedures

#### Blood sampling

Up to 30 ml of blood was sampled to assess antibody persistence at 6, 15 and 22-months in the different groups of participants as described above. For those who received booster immunizations at 6 months, blood was sampled prior to and 21 days after immunization. For those who received the 22-month booster immunization, blood was sampled just prior to booster immunization and at 7 and 21 days after immunization. The 7-day blood was taken to look for evidence of early response suggestive of immunological memory. Finally those immunized with a two-dose schedule of the clade 2 vaccine as a primary course had blood sampled prior to each immunization and 21 days after the second dose.

#### Booster vaccines

The vaccines were inactivated, split-virion preparations of vaccine strains; clade 1: A/Vietnam/1194/2004/NIBRG-14 (H5N1) (7.5 μg HA without adjuvant) or clade 2: A/Indonesia/5/05-RG2 (H5N1) (30 μg with Al(OH)_3_), propagated in embryonated eggs. The dose of Al(0H)_3_, used as the adjuvant, was 600 μg/dose (expressed as the content of Al3+). Both formulations were presented in ready-to-use multi-dose vials from which 0.5 ml were withdrawn and injected intramuscularly into the deltoid muscle using a standard syringe and 25 G, 25 mm needle.

### Safety and Reactogenicity Analysis

After each immunization participants were monitored for 30 minutes for immediate reactions. Participants kept diaries in which they recorded any solicited (prelisted) local or systemic reactions that occurred up to 7 days after immunization. Specifically participants were asked to record injection site induration, injection site ecchymosis, temperature, malaise and shivering for 3 days after vaccination. Participants were also asked to record any other adverse event that occurred up to 21 days after immunization. Serious adverse events were collected throughout the study. Participants were followed up between 7 and 28 months (12 months for the group receiving the clade 1 A(H5N1)/Vietnam booster; 22 months for participants who took part in the primary phase of the study only; 28 months for participants who received the clade 2 A/Indonesia booster at 22 months and 7 months for 50 additional participants primed with the clade 2 A(H5N1N)/Indonesia.

### Antibody Response

Serum samples were used for antibody titration against one of the vaccine strains (clade 1 or clade 2) using haemagglutination inhibition (HI) and seroneutralization (SN) assays.

The HI assay reflects the ability of the specific anti-influenza virus antibodies to inhibit haemagglutination of erythrocytes by influenza virus as previously described [8]. Briefly, the HI assays were performed on serial twofold dilutions of all available samples using horse erythrocytes (lower detection limit 1:8; seroresponse threshold of 1:32).

The SN assay is based on the ability of antibodies to inhibit the infection of Madin-Darby canine kidney (MDCK) cell culture, as previously described [8]. Briefly, inactivated human serum was incubated with virus then added to the MDCK cell culture. After an overnight incubation, enzyme-linked immunosorbent assay was used to measure the degree of infection of the MDCK. The lower level of detection of this assay was 1:20.

In all assays samples were tested in duplicate and the titer analyzed was the geometric mean of the duplicates, expressed as the reciprocal of dilution. Samples without detectable antibody activity were assigned the titer of half the assay detection limit. These analyses were performed under blinded conditions at the Public Health England laboratories (Colindale, London, UK).

### Statistical Analysis

Sample size for the additional participants receiving a primary vaccination series of the clade 2 vaccine A(H5N1)/Indonesia, was arbitrarily set at 50 subjects. The sample size required to determine the booster response to clade 2 A(H5N1)/Indonesia was deemed to be lower than the initial cohort size, as only half the original study population were eligible to continue in the study, i.e. those who received the higher dose vaccine during the primary phase. Accordingly, participants at centre 4 were not invited to take part in the booster phase of the study. The minimal data sets underlying the findings are available to interested researchers who in the first instance may request access to anonymized patient-level data and clinical study documents at www.clinicalstudydatarequest.com. If further assistance is required then the corresponding author is to be contacted.

The study was descriptive and no formal statistical comparisons between groups have been made. The results are presented with 95% confidence intervals of point estimates calculated using normal approximation for quantitative data and exact binomial distribution (Clopper-Pearson method) for proportions. The population analyzed for all the timepoints presented here are the full analysis set. A per-protocol analysis was pre-designated for the A(H5N1) vaccine naïve group receiving the primary course with clade 2 vaccine, however there were no protocol deviations in this group therefore the per protocol and full analysis set were equivalent. For the groups that received either booster vaccination the full analysis set is defined as all those who received the booster immunization. The analysis included all data available at each timepoint.

HI data are expressed as GMT’s in accordance with criteria based on those defined for the HI method by the committee for Medicinal Products for Human Use (CHMP) for seasonal influenza vaccines which are applied to pandemic vaccines in absence of specific alternative criteria. These criteria are: a seroprotection rate (% with HI titers ≥ 40 dil-1) of over 70%; a rate of seroconversion or significant titer increase (pre-vaccination titer of < 8 and post-vaccination titer ≥ 32, or ≥ 4-fold titer increase between pre and post titers) of over 40% and an increase in geometric mean titer ratio (GMTR) between post and pre vaccination titer by over 2.5. In adults over the age of 60 these criteria are modified to a seroprotection rate of over 60%, seroconversion rate or significant increase in titer of over 30% and a GMTR of over 2 [9]. It is recommended that antibody persistence is also evaluated to help guide re-immunization practices, however there are no formal immunogenicity criteria provided. Only data relating to HI antibodies are presented here.

Analyses were performed by the Sanofi Pasteur Biostatistics Department, Marcy I’Etoile, France, using SAS software version 8.2 (SAS Institute, Cary NC, USA)) and were independently validated by the Oxford Vaccine Group, University of Oxford, using SAS version 9.3 (M.V.).

## Results

At 6-months after the start of the primary phase of the study 587 out of the original 600 participants remained eligible to continue in the study (Fig 1). For the 6-month, 7.5 μg booster arm, 155 participants completed the study at this point. For the 22-month, clade 2, 30 μg alum-adjuvanted booster arm 118 participants completed the study at this point. Antibody persistence after primary vaccination was evaluated at 6, 15 and 22 months in 587, 409 and 192 participants, respectively. Additional information on the procedures performed at each study visit can be found in S1 Fig (trial flow charts) that compliments the Fig 1.

### Safety and Reactogenicity

In total 33 serious adverse events (SAEs) have been reported in this phase of the study, that is up to 42 days after the start of the study. SAEs that occurred in the primary phase are reported in the primary phase publication [8]. There were no SAE in the 21 days following receipt of either strength of booster or primary clade 2 vaccination. None of the SAEs were considered by the investigators to be related to vaccination.

Up to 21 days after the booster dose of the 7.5 μg clade 1 A(H5N1)/Vietnam vaccine the percentage of adults aged between 18 and 60 years reporting at least one solicited reaction was 50% in those primed with the high dose adjuvanted vaccine and 35.9% in those primed with the low dose non-adjuvanted vaccine. In adults aged over 60 years at least one solicited event was reported by 19.5% and 27.8% in the high dose and low dose priming groups respectively.

Up to 21 days after booster immunization with the high-dose alum-adjuvanted clade 2 A/Indonesia vaccine 74.6% of adults aged between 18 and 60 reported at least one solicited injection site reaction and 42.4% at least one solicited systemic reaction whilst 36.8% and 33.3% of those aged over 60 reported at least one solicited injection site and systemic reaction, respectively.

Most common reactions in both groups were pain at the injection site, headache and malaise which started within 3 days of vaccination. Unsolicited reactions that started after 21 days were considered not related to the vaccine. A break down of the type of reactions in the first 7 days after immunization is given in Table 1.

**Table 1:** Solicited adverse events following booster vaccination or in case of clade 2 primary vaccination within 3 to 7-days after vaccination.

Age groups	18–60 years						> 60 years							18–60 years	
Primary vaccine dose	7.5 μg(n = 39)			30 μg + Adjuvant (n = 38)	30 μg +Adjuvant (n = 60)		7.5 μg(n = 36)		30 μg +Adjuvant(n = 41)			30 μg +Adjuvant(n = 58)		No previous H5N1 vaccine(n = 50)	
Booster dose	7.5 μg Booster group				Clade 2 Booster Group		7.5 μg Booster group					**Clade 2 Booster Group**		**Clade 2 Priming group**	
	%	95% CI	%	95% CI	%	95% CI	%	95% CI		%	95% CI	%	95% CI	%	95% CI
**Injection site reactions**															
Pain	20.5	9.3;36.5	28.9	15.4;45.9	61	47.4;73.5	8.3	1.8;22.5		7.3	1.5;19.9	28.1	17.0;41.5	44	30;58.7
Erythema	12.8	4.3;27.4	13.2	4.4;28.1	25.4	15.0;38.4	2.8	0.1;14.5		0.0	0.0;8.6	12.3	5.1;23.7	10	3.3;21.8
Swelling	0	0;9.0	2.6	0.1;13.8	15.3	7.2;27	5.6	0.7;18.7		0.0	0.0;8.6	8.8	2.9;10.3	8	2.2;19.2
Induration*	7.7	1.6;20.9	2.6	0.1;13.8	25.4	15;38.4	0.0	0.0;9.7		0.0	0.0;8.6	8.8	2.9;10.3	14	5.8;26.7
Ecchymosis*	0.0	0.0;9.0	0	0.0;9.3	5.1	1.1;14.1	0.0	0.0;9.7		2.4	0.1;9.7	5.2	1.9;10.9	2	1.3;16.5
**Systemic Reactions**															
Fever*	0.0	0.1;12.9	2.6	01;13.8	3.4	0.4;11.7	2.8	0.1;14.5		2.4	0.1;12.9	5.3	1.1;14.6	4	0.5;13.7
Headache	17.9	7.5;33.5	18.4	7.7;34.3	30.5	19.2;43.9	13.9	4.7;29.5		9.8	2.7;23.1	19.3	10;31.9	26	14.6;40.3
Malaise*	5.1	0.6;17.3	7.9	1.7;21.4	16.9	8.4;29	2.8	0.1;14.5		2.4	0.1;12.9	10.5	4.0;21.5	16	2.2;19.2
Myalgia	5.1	0.6;17.3	7.9	1.7;21.4	18.6	9.7;30.9	5.6	0.7;18.7		4.9	0.6;16.5	10.5	4.0;21.5	10	1.3;21.8
Shivering*	0.0	0.0;9.0	5.3	0.6;17.7	6.8	1.9;16.5	0.0	0.0;9.7		0.0	0.0;8.6	5.3	1.1;14.6	6	0.0;7.1

n = number of participants’ data analysed, based on the number of subjects who received at least 1 dose of vaccine

*participants were asked to specifically record these adverse events for 3-days after vaccination

In the naïve group (18–60 years old) who received a primary course of the clade 2 A(H5N1)/Indonesia vaccine no SAEs were reported. Overall 72% of participants experienced a solicited reaction within 21 days of immunization. Injection site pain and headache were the most commonly reported reactions in the first 7 days after immunization (Table 1).

### Immunogenicity

#### Antibody persistence

At 6 months after primary immunization with the 7.5 μg clade 1 A(H5N1)/Vietnam vaccine the percentage of individuals who had antibody levels above the threshold for seroprotection (defined as HI antibody titer **≥**32) was 6.2% in the 18 to 60 years group and 14.4% in the over 60 years group (Table 2).

**Table 2:** Antibody persistence as defined by HI antibody titer at 6/15/22-months following the first vaccination with clade 1 A/Vietnam/1194/2004/NIBERG-14.

Age Groups	160 years				> 60 years			
Primary vaccination	7.5 μg	30 μg + Adjuvant			7.5 μg	30 μg + Adjuvant		
**Time (months) since vaccination**	**6 (n = 146)**	**6 (n = 146)**	**15 (n = 98)**	**22 (n = 95)**	**6 (n = 146)**	**6 (n = 148)**	**15 (n = 101)**	**22 (n = 94)**
% subjects with GMT ≥ 32 (95% CI)	6.2 (2.9; 11.4)	6.2 (2.9; 11.4)	3.1 (0.6; 8.7)	1.1 (0.0; 5.7)	14.4 (9.1; 21.1)	18.9 (13.0; 26.2)	12.9 (7.0; 21)	11.7 (6.0; 20)
GMT (95% CI)	5.43 (4.85; 6.08)	5.99 (5.33; 6.73)	4.6 (4.20; 5.06)	4.33 (4.03; 4.66)	7.63 (6.41; 9.08)	9.42 (7.78; 11.42)	6.8 (5.55; 8.30)	6.37 (5.22; 7.76)

GMT: Geometric mean titer n = number of participants

In the group who had received the 30 μg adjuvanted clade 1 A(H5N1)/Vietnam vaccine, 6.2% of participants aged 18 to 60 and 18.9% of participants aged over 60 had HI titres > 32. Again these levels decreased with time (Table 2).

#### Booster and primary responses

Booster response to 7.5 μg clade 1 A/Vietnam vaccine at 6 months (Table 3).

**Table 3:** 21 Day Booster response to 7.5 μg clade 1 A/Vietnam/1194/2004/NIBERG-14 6-months after the first vaccination.

Age group		18 to 60 years		> 60 years	
Primary vaccine dose		7.5 μg (N = 39)	30 μg + Adjuvant (N = 38)	7.5 μg (N = 36)	30 μg + Adjuvant(N = 41)
	EMA threshold				
GMT (95% CI)	None	8.67 (6.32; 11.89)	9.96 (7.17; 13.82)	11.53 (7.56; 17.59)	18.8 (12.5; 28.3)
GMTR (95% CI)	>2.5	1.59 (1.22; 2.06)	1.45 (1.21; 1.75)		
GMTR (95% CI)	> 2^#^			1.31 (1.06; 1.62)	1.34 (1.09; 1.67)
Seroconversion* % (95% CI)	> 40%	10.3 (2.9; 24.2)	2.6 (0.1; 13.8)		
Seroconversion* % (95% CI)	> 30%^#^			2.8 (0.1; 14.5)	9.8 (2.7; 23.1)
Seroprotection (titre ≥ 40) % (95% CI)	> 70%	12.8 (4.31; 27.4)	13.2 (4.4; 28.1)		
Seroprotection (titre ≥ 40) % (95% CI)	> 60%^#^			19.4 (8.2; 36.0)	29.3 (16.1; 45.5)

n = number of participants

EMA: European Medicines Authority

GMT: Geometric mean titer

GMTR: Geometric mean titer ratio (post-vaccination titer/pre-vaccination titer)

*Seroconversion: subjects with pre-vaccination titers < 8 and post-vaccination titres > 32 or significant increase in titres defined as pre-vaccination titre < 8 with at least a 4-fold increase in post-vaccination titers

# Modified EMA criteria for adults aged over 60 years of age

The response to this booster vaccine at 6 months after the primary immunisation as measured by HI antibody did not meet any of the CHMP criteria in either of the two age groups.

Booster response to 30μg with adjuvant clade 2 A/Indonesia vaccine at 22 months (Table 4).

**Table 4:** Booster response to 30 μg adjuvanted clade 2/ Indonesia/5/05-RG2 vaccine at 22 months following the first vaccination.

			18 to 60 years		> 60 years	
Strain used to test response			Clade 1 (Vietnam)	Clade 2 (Indonesian)	Clade 1 (Vietnam)	Clade 2 (Indonesian)
	EMA threshold	Time after vaccination (days)	N = 59	N = 59	N = 57	N = 57
GMT (95% CI)	None	7	12.1 (9.35; 15.8)	12.6 (9.56; 16.5)	8.25 (6.25; 10.9)	6.16 (5.02; 7.56)
		21	14.4 (10.8; 19.2)	15.2 (11.3; 20.3)	10.7 (7.71; 14.9)	9.84 (7.06; 13.7)
GMTR (95% CI)	>2.5 or > 2^#^	7	2.93 (2.28; 3.77)	3.07 (2.34; 4.03)	1.54 (1.25; 1.90)	1.49 (1.21; 1.82)
		21	**3.47 (2.63; 4.59)**	**3.71 (2.78; 4.94)**	2.01 (1.50; 2.71)	2.39 (1.72; 3.33)
Seroconversion* % (95% CI)	> 40% or 30%^#^	7	27.1 (16.4; 40.3)	28.8 (17.8; 42.1)	12.5 (5.2; 24.1)	8.9 (3.0; 19.6)
		21	39 (26.5; 52.6)	**42.4 (29.6; 55.9)**	17.9 (8.9; 30.4)	21.4 (11.6; 34.4)
GMTtitre ≥ 32% (95% CI)	> 70% or > 60%^#^	7	27.1 (16.4; 40.3)	28.8 (17.8; 42.1)	22.8 (12.7; 35.8)	10.5 (4.0; 21.5)
		21	39 (26.5; 52.6)	42.2 (29.6; 55.9)	26.3 (15.5; 39.7)	22.8 (12.7; 35.8)

EMA: European Medicines Authority

GMT: Geometric mean titer

GMTR: Geometric mean titer ratio (post-vaccination titer/pre-vaccination titer)

*Seroconversion: subjects with pre-vaccination titers < 8 and post-vaccination titres > 32 or significant increase in titres defined as pre-vaccination titre < 8 with at least a 4-fold increase in post-vaccination titers about 3 weeks after vaccination

# Modified EMA criteria for adults aged over 60 years of age

Numbers shown in bold are those which meet the specified EMA criterion

The booster response was tested against both clade 1 and 2 viruses. At 7 days after the booster immunization the GMTR was above the threshold required by the CHMP (fulfilling 1 out 3 requirements) in the 18 to 60 year group against both clade 1 and clade 2 strains but not in the over 60 age group.

By 21 days after immunization in the 18 to 60 year age group 2 out of 3 CHMP criteria (seroconversion rate and GMTR) were fulfilled when tested against the clade 2 virus and a single criterion (GMTR) was met when tested against the clade 1 virus. In the over 60 year group the only CHMP criterion met was the GMTR for both of the viral strains tested.

Primary Response to clade 2 vaccine (Table 5).

**Table 5:** Primary response following clade 2 A/Indonesia /5/05-RG2 as determined by HIH method.

	EMA threshold	Time after vaccination (days)	Primary vaccination 30 μg + adjuvant (n = 50)
GMT (95% CI)	None	0	4.0 (4.0; 4.0)
		21	4.4 (4.08; 4.760
		42	12.8 (8.84; 18.6)
GMTR % (95% CI)	> 2.5	21	2.9 (2.08; 4.06)
		42	**3.20 (2.21; 4.64)**
Seroconversion% (95% CI)	> 40%	21	0.0 (0.0; 7.1)
		42	26.0 (14.6; 40.3)
Seroprotection % (titre ≥ 32)	> 70%	0	0.0 (0.0;7.1)
		21	0.0 (0.0; 7.1)
		42	26 (14.6; 40.3)

n = number of participants

EMA: European Medicines Authority

GMT: Geometric mean titer

GMTR: Geometric mean titer ratio (post-vaccination titer/pre-vaccination titer)

*Seroconversion: subjects with pre-vaccination titers < 8 and post-vaccination titres > 32 or significant increase in titres defined as pre-vaccination titre < 8 with at least a 4-fold increase in post-vaccination titers

In the naïve group who received the clade 2 vaccine only, prior to immunization there was no evidence of prior immunity to the clade 2 A/Indonesia virus. Twenty-one days after a single dose of vaccine one CHMP criterion (GMTR over 2.5) was met. This did not change 21 days after the second immunization. The demographics of the booster and naïve group are given in the supplementary material.

## Discussion

This study investigated the potential usefulness of a prime-boost vaccination strategy using two different influenza A(H5N1) strains to induce cross clade protection. The data show that a heterologous booster vaccine strain can induce cross-clade reactivity. Despite the fact that the primary vaccination schedule had induced antibody responses that met CHMP criteria in some groups, 6 months after the booster dose antibody levels had waned below the CHMP thresholds established for primary vaccination. It is important to stress here that no CHMP criteria exist for the duration of antibody persistence and the extent of cross-clade reactivity of influenza vaccines. It may not be indicated to appreciate the value of this prime-boost strategy using a possibly non-appropriate scoring system [9].

### Antibody Persistence

Few reports are available on antibody persistence with influenza A(H5N1) vaccines exceding a 6 month period whereas a pandemic threat can persist for several years. Lin et al., (2009) reported that 6-months after immunization with low dose, alum-adjuvanted, whole virion A(H5N1) vaccine antibodies were only present in 4.8% to 20.8% of adults aged under 60 years and thus failed to meet any CHMP at this time point [10]. In contrast, it has been demonstrated that antibodies induced by a low dose influenza A(H5N1) vaccine formulated with an oil-in-water adjuvant persist at levels that meet all 3 CHMP criteria 6-months after immunization [11]. In head-to-head comparisons of low-dose, unadjuvanted vaccines and oil-in-water emulsion-adjuvanted vaccines, the latter were shown to elicit higher antibody titres that persisted much longer in both young and old adults [12, 13].

In the present study participants were followed for 22 months following the first immunization, which is the longest follow-up period reported. Six months after primary vaccination the magnitude of HI titres differed more by age group than by primary vaccination group (low-dose, unadjuvanted vs. high-dose, alum-adjuvanted). In the younger age group 6% of participants had HI titres detected at 6 months, irrespective of the type of vaccine administered. In the older age group 14.4% and 18.9% had detectable HI titres with the low- and high-dose vaccine, respectively. This trend continued for 22 months after immunization and may reflect the effect of prior priming via natural exposure or seasonal influenza vaccine in the older group. In fact even at baseline, prior to A(H5N1) immunization, 16% of the older age group had elevated HI titres; similar results have been found by other investigators [14,15].

### Cross-Clade Immunity

Vaccine-induced cross-clade immune response is defined as the ability to induce immune responses against pathogen strains that are genetically distinct from the strain(s) used to produce the vaccine [16]. Practically this infers that immunization with a vaccine containing antigen(s) from one clade of pandemic virus may elicit some degree of protection against a genetically distinct virus strain. A cross-reactive immune response between H5N1 vaccines would potentially allow dose-sparing during subsequent waves of the pandemic. Cross-reactivity between avian influenza viral clades has previously been demonstrated using vaccines containing oil-in-water emulsions as adjuvants, such as MF59 and AS03 and whole-virion vaccines [17–22]. A primary course of a low-dose clade 1 vaccine containing an oil-in-water adjuvant induced antibodies against a clade 2 virus in 77% of study participants [21]. When the same antigen was administered without any adjuvant no cross-reactive response was observed, demonstrating the importance of the adjuvant for the induction of cross-reactivity [22]. Wu et al., (2009) used a two-dose schedule of whole-virion vaccine containing an alum-adjuvant. Twenty-eight days after the second vaccine dose seroconversion was induced against two heterologous clade 2 strains tested in more than 40% of the participants 28 days, thus meeting 1 out of 3 CHMP criteria [23]. To our knowledge no direct comparisons of cross-reactivity between vaccines containing oil-in-water-based adjuvants and alum-adjuvanted vaccines or alum-adjuvanted vaccines and non-adjuvanted vaccines have been performed. Studies that have compared immunogenicity of alum-adjuvanted vaccines with that of non-adjuvanted vaccines suggest that aluminium does not improve the immunogenicity of the vaccines, in particular when low antigen doses are used [24, 25]. Whether the presence of alum contributes to cross-reactivity remains unanswered.

The main study limitation stemmed from the lower than expected antibody responses to the low dose vaccine described in the primary phase of the study [8] this meant that that only those who received the high dose vaccine with adjuvant were invited to have the Clade 2 booster vaccine. The loss of half of all study participants by the amendment to study design meant that there was inadequate power for a formal statistical analysis for the booster phase of the study therefore centre 4 was not included on grounds of commercial feasibility to allow the remainder of the study to go one. The amended study design also accounts for the arbitrary sample size of the group who received primary vaccination with the clade 2 vaccine.

In conclusion antibody cross-reactivity between A(H5N1) clades suggests that in principle a prime-boost vaccination strategy may provide both early protection at the start of a pandemic and improved antibody responses to specific vaccination once available. Further criteria to assess this strategy are needed.

## Supporting Information

S1 Fig Trial Flow Chart. (DOCX)

S1 File Safety and Immunogenicity of an Intramuscular, Inactivated, Split-Virion, Pandemic Influenza A/H5N1 Vaccine in Adults and the Elderly Clinical Trial Protocol, Amendment 3. (PDF)

S2 File TREND Statement Checklist. (PDF)

S1 Table Baseline Characteristics of Groups at time of first vaccination. (DOCX)
