# Supplementary figures and images for: Antibody Persistence and Booster Responses to Split-Virion H5N1 Avian Influenza Vaccine in Young and Elderly Adults
Source: PLoS One. 2016 Nov 4;11(11):e0165384. doi: 10.1371/journal.pone.0165384 (PMC5096706; doi:10.1371/journal.pone.0165384)

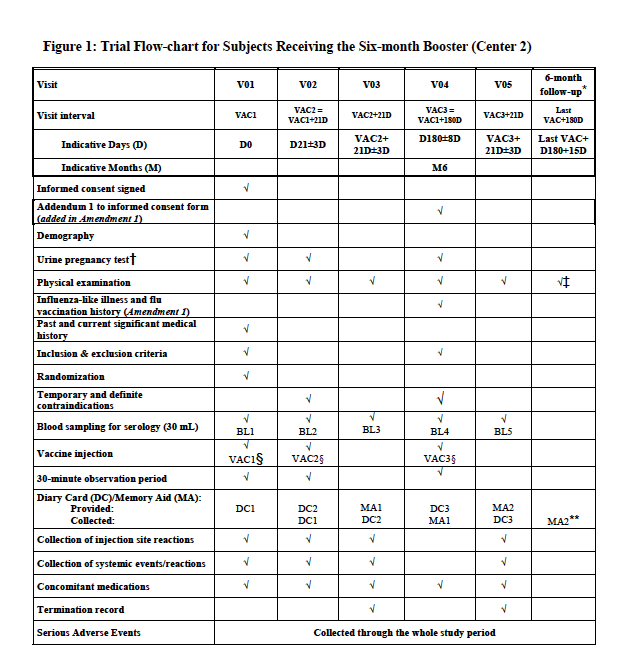


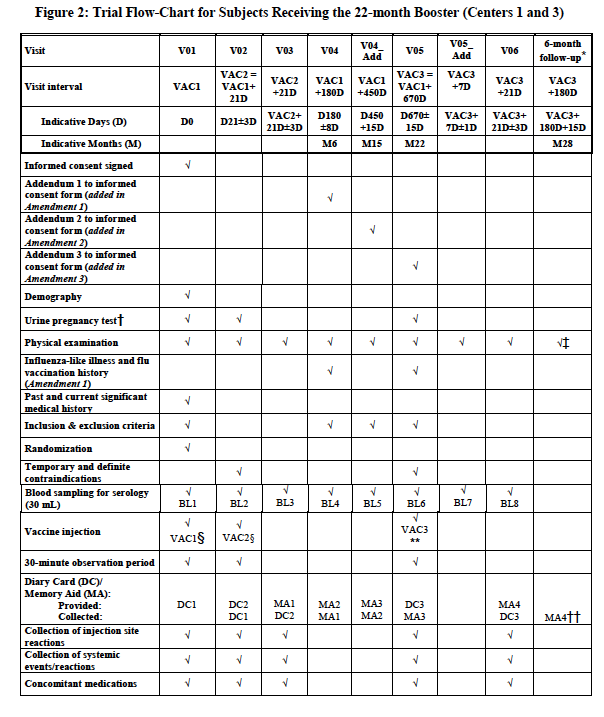


Figure 2 Continued


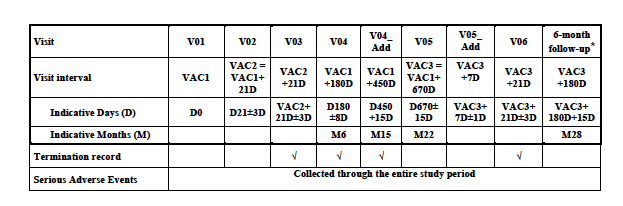

Supplement: S1 Fig — (DOCX) [file pone.0165384.s001.docx]
