# Supplementary material for: Antibody Persistence and Booster Responses to Split-Virion H5N1 Avian Influenza Vaccine in Young and Elderly Adults
Source: PLoS One. 2016 Nov 4;11(11):e0165384. doi: 10.1371/journal.pone.0165384 (PMC5096706; doi:10.1371/journal.pone.0165384)
Supplement: S1 File — (PDF) [file pone.0165384.s002.pdf]

Redaction of

Safety and Immunogenicity of an Intramuscular, Inactivated, Split-Virion, Pandemic Influenza A/H5N1 Vaccine in Adults and the Elderly

A Phase II, open, randomized, multicenter trial in 600 subjects divided into two groups according to the vaccine formulation used (7.5 µg HA without adjuvant and 30 µg HA with adjuvant of an A/Vietnam H5N1 vaccine strain). Each subject will receive two administrations 21 days apart as primary vaccination series. Among these 600 subjects, a subset of 160 subjects will receive a booster vaccination at 6 months (with 7.5 µg HA without adjuvant of the same vaccine strain as the primary vaccination series). A subset of subjects receiving the 30 µg HA with adjuvant formulation as primary vaccination series (N=140) will receive a booster vaccination at 22 months (with 30 µg HA with adjuvant of an A/Indonesia H5N1 vaccine strain). The remaining subjects will receive no booster. *(Amendments 1, 2 and 3).*

In addition, a further 50 subjects will be vaccinated with 30 µg HA with adjuvant of an A/Indonesia H5N1 vaccine strain, and will receive no booster *(Amendments 1 and 3)*.

Clinical Trial Protocol, Amendment 3

Trial Code: GPA02 **Development Phase:** II

Sanofi Disclosure Statement:

Making clinical trial data, information and results available to researcher's promises to advance science and medicine, contribute to improvements in public health, and improve knowledge about, and trust in, pharmaceutical product development. While making information available we will continue to protect the privacy of the participants in our clinical trials and to maintain incentives for investment in biomedical research through redaction of proprietary and commercially confidential information (CCI).

Please visit www.clinicalstudydatarequest.com to review Sanofi's data sharing guidelines.

The following guiding principles for redaction have been applied:

- Information will be excluded to protect the privacy of study participants, participating organizations, and all named persons associated with the study
- Individual patient-/subject-specific data will be completely removed to protect study participants privacy. De-identified patient/subject-level data may be available for approved research proposals
- Aggregate data will be included; any direct reference to individual patients/subjects will be excluded
- Some appendices will not be provided if the relevant information is supplied in the main body of the CSR.

sanofi pasteur

The vaccines business of sanofi-aventis Group

Safety and Immunogenicity of an Intramuscular, Inactivated, Split-Virion, Pandemic Influenza A/H5N1 Vaccine in Adults and the Elderly

A Phase II, open, randomized, multicenter trial in 600 subjects divided into two groups according to the vaccine formulation used (7.5 µg HA without adjuvant and 30 µg HA with adjuvant of an A/Vietnam H5N1 vaccine strain). Each subject will receive two administrations 21 days apart as primary vaccination series. Among these 600 subjects, a subset of 160 subjects will receive a booster vaccination at 6 months (with 7.5 µg HA without adjuvant of the same vaccine strain as the primary vaccination series). A subset of subjects receiving the 30 µg HA with adjuvant formulation as primary vaccination series (N=140) will receive a booster vaccination at 22 months (with 30 µg HA with adjuvant of an A/Indonesia H5N1 vaccine strain). The remaining subjects will receive no booster. *(Amendments 1, 2 and 3).*

In addition, a further 50 subjects will be vaccinated with 30 µg HA with adjuvant of an A/Indonesia H5N1 vaccine strain, and will receive no booster *(Amendments 1 and 3).*

Clinical Trial Protocol, Amendment 3

IND Number: Not applicable

EUDRACT Number: 2006-000477-29

Trial Code: **GPA02**

Development Phase: II

Sponsor: Sanofi Pasteur SA

2, avenue Pont Pasteur, 69367 Lyon cedex 07, France

Investigational Product: A/H5N1 inactivated, split-virion influenza virus

Form/Route: Liquid/Intramuscular

Manufacturer: [redacted]

Coordinating Investigators: Coordinating Investigator: [redacted] MD ([redacted] Belgium), [redacted] MD ([redacted] UK)

Sponsor's Responsible Medical Officer (Sponsor's signatory): [redacted] MB BS – Clinical Franchise Leader [redacted]

Clinical Scientist: [redacted] PhD [redacted]

Clinical Research Associate (CRA): [redacted]

[redacted] [redacted]

Version and Date of the Protocol: Version 5.0, dated 30 November 2007

This protocol (Version 5.0) is the third amendment to the initial trial protocol (Version 2.0, dated 16 February 2006). The first and second amendments were Version 3.0, dated 06 September 2006, and Version 4.0 dated 20 April 2007, respectively.

Synopsis

**Company:**	sanofi pasteur
**Investigational Product/ Drug Product:**	A/H5N1 inactivated, split-virion influenza vaccine made in embryonated eggs
**Active Substance(s):**	Inactivated influenza virus split with octoxinol 9

**Title of the trial:**	Safety and Immunogenicity of an Intramuscular, Inactivated, Split-Virion Pandemic Influenza A/H5N1 Vaccine in Adults and the Elderly
**Development phase:**	II
**Coordinating Investigators:**	Coordinating Investigator: [redacted] MD ([redacted] Belgium), [redacted] MD ([redacted] UK)
**Investigators and trial centers:**	This is a multicenter, multinational trial.<br>[redacted] MD - [redacted] (Center 1).<br>[redacted] MD - [redacted] Belgium. (Center 2).<br>[redacted] MD - [redacted] UK. (Center 3).<br>[redacted] MD - [redacted] Belgium. (Center 4).<br>[redacted]
**Planned trial period:**	May 2006 (First Visit First Subject) to July 2007 (for 6-month booster) (Last Visit Last Subject) or October 2008 (for A/Indonesia booster and for the 50 additional subjects receiving the A/Indonesia H5N1 strain as primary series) (Last Visit Last Subject) *(Amendment 3)*
**Objectives:**	• To describe the injection site reactions and systemic safety profile during the 21 days following each of two primary series and one booster (as applicable) intramuscular (IM) injections in two age groups: subjects aged 18 to 60 years (adults) or >60 years (elderly) *(Amendments 1 and 2)*.<br>• To describe the immune response 21 days after each of two primary series IM injections in two age groups: subjects aged 18 to 60 years (adults) and >60 years (elderly) *(Amendment 2)*.<br>• To describe the antibody persistence at M6 (D180) (for all subjects), and at, M15 and M22 (in a subset of subjects) after the first vaccination in two age groups: subjects aged 18 to 60 years (adults) or >60 years (elderly) *(Amendments 2 and 3)*.<br>• To describe the immune response 21 days after a booster vaccination administered at 6 months (A/Vietnam booster) or 7 and 21 days after a booster vaccination administered at 22 months (A/Indonesia booster) after the first vaccination in two age groups: subjects aged 18 to 60 years or >60 years *(Amendments 1, 2 and 3)*.<br>• To describe any serious adverse events (SAEs) during the entire trial.
**Endpoints:**	Safety<br>• The occurrence, time to onset, number of days of occurrence, and severity of solicited (prelisted in the subject diary and Case Report Form [CRF]) injection site

**Company:**	sanofi pasteur
**Investigational Product/ Drug Product:**	A/H5N1 inactivated, split-virion influenza vaccine made in embryonated eggs
**Active Substance(s):**	Inactivated influenza virus split with octoxinol 9

reactions and systemic reactions occurring within 7 days following each injection will be reported.

- The occurrence, nature (Medical Dictionary for Regulatory Activities [MedDRA preferred term), time to onset, duration, severity, relationship to vaccination and seriousness of unsolicited (spontaneously reported) adverse events (AEs) within 21 days following each injection will be reported.
- The occurrence of the following reactions (MedDRA Preferred Terms given in parentheses) in the 3 days following each injection will be more especially reported (as defined by the European Medicines Agency (EMEA) Note for Guidance [CPMP/BWP/214/96]):
    - Injection site induration >5 cm observed for more than 3 days.
    - Injection site ecchymosis (injection site hemorrhage).
    - Rectal equivalent temperature >38°C for 24 hours or more.
    - Malaise.
    - Shivering (chills).
- The occurrence, nature, time to onset, and relationship to vaccination of SAEs during the whole trial (including the 6-month follow-up period) will be reported.

Immunogenicity

Primary series

Hemagglutination inhibition assays will be performed using:

1. Horse erythrocytes (hemagglutination inhibition using horse erythrocytes [HIH] method) for all subjects.
2. Turkey erythrocytes (hemagglutination inhibition using turkey erythrocytes [HIT] method) for a subset of 80 subjects in each formulation group (40 subjects per age sub-group) for one vaccine strain only - H5N1 A/Vietnam: A/Vietnam/1194/2004/NIBRG-14 (H5N1) (henceforth referred to as A/Vietnam) (*Amendment 1*).

Anti-Hemagglutinin (anti-HA) titers against the A/H5N1 strain will be expressed as described below:

- Anti-HA Ab titer obtained in duplicate on D0, D21, and D42, and summarized at the subject level by individual geometric means of duplicates at each timepoint. The following endpoints will be derived:
    - Individual titer ratios D21/D0, D42/D0, and D42/D21.
    - Proportion of subjects with titer ≥40 (turkey) or ≥32 (horse) 1/dilution (dil) on D0, D21 and D42.
    - Seroconversion (for subjects with a titer <10 [turkey] or <8 [horse] [1/dil] on D0: post-injection titer ≥40 [turkey] or ≥32 [horse] [1/dil])

    or

    - significant increase (for subjects with a titer ≥10 [turkey] or ≥8 [horse] [1/dil]: ≥4-fold increase of the titer) at D21 and D42.

**Company:**	sanofi pasteur
**Investigational Product/ Drug Product:**	A/H5N1 inactivated, split-virion influenza vaccine made in embryonated eggs
**Active Substance(s):**	Inactivated influenza virus split with octoxinol 9

	AND<br>Neutralizing Ab titers against the A/H5N1 strain will be expressed as described below<br>• Neutralizing Ab titer obtained in duplicate on D0, D21, and D42 and summarized at the subject level by individual geometric mean of duplicates at each timepoint. The following endpoints will be derived:<br>• Individual titer ratios D21/D0, D42/D0, and D42/D21.<br>• 2- and 4-fold increase from D0 to D21 and to D42.<br>**Antibody persistence (Amendment 3)**<br>Anti-HA Ab titer (HIH method) and neutralizing Ab titer (seroneutralization [SN] method) at M6 (D180) (for all subjects), and at M15 and M22 (for a subset subjects).<br>**Booster vaccination response (Amendment 3)**<br>The A/Vietnam H5N1 strain will be used for the 6-month booster, and an A/Indonesia/5/05-RG2 (H5N1) strain (henceforth referred to as A/Indonesia) will be used for the 22-month booster (see Methodology/Trial Design). The booster Ab response will be assessed against the homologous strain in the vaccine, with HI test using horse erythrocytes and SN method. In addition, the antibody response to heterologous strains may be tested.<br>• Anti-HA Ab titer obtained in duplicate at M6 (D180) and M6+21 days (D201) (if 6-month A/Vietnam booster) or M22 (D670), M22+7 days (D677) and M22 +21 days (D691) (if 22-month A/Indonesia booster), and summarized at the subject level by individual geometric means of duplicates at each timepoint. The following endpoints will be derived:<br>• Individual titer ratios M6+21D/M6 (if 6-month A/Vietnam booster) or, M22+7D/M22 and M22+21D/M22 (if 22-month A/Indonesia booster).<br>• Proportion of subjects with anti-HA Ab titer ≥32 (1/dil) at M6 and M6+21D (if 6-month A/Vietnam booster) or M22, M22+7D and M22+21D (if 22-month A/Indonesia booster).<br>• Seroconversion: for subjects with a titer <8 (1/dil) at M6 or M22: post-injection titer ≥32 [1/dil]) at M6+21D (if 6-month A/Vietnam booster), or M22+7D or M22+21D (if 22-month A/Indonesia booster), or a significant increase (for subjects with a titer ≥8 [1/dil]: ≥4-fold increase of the titer) at M6+21D (if 6-month A/Vietnam booster), or M22+7D and M22+21D (if 22-month A/Indonesia booster).<br>AND<br>• Neutralizing Ab titer obtained at M6 and M6+21D (if 6-month A/Vietnam booster) or M22, M22+7D and M22+21D (if 22-month A/Indonesia booster). The following endpoints will be derived:<br>• Individual titer ratios M6+21D/M6 (if 6-month A/Vietnam booster)or M22+7D/M22 and M22+21D/M22 (if 22-month A/Indonesia booster).<br>• 2- and 4-fold increase from M6 to M6+21D (if 6-month A/Vietnam booster) or from M22 to M22+7D and from M22 to M22+21D (if 22-month A/Indonesia booster).

**Company:**	sanofi pasteur
**Investigational Product/ Drug Product:**	A/H5N1 inactivated, split-virion influenza vaccine made in embryonated eggs
**Active Substance(s):**	Inactivated influenza virus split with octoxinol 9

**Statistical methods for the objectives:**	The safety analysis will describe the incidence of solicited and unsolicited AEs, during 21 days after each injection (including booster injections).<br>95% confidence intervals (CIs) of point estimates of predefined solicited reactions will be calculated using the exact binomial distribution (Clopper-Pearson method) for proportions.<br>Additionally, for each injection, the safety data will be presented according to the EMEA safety criteria.
**Observational Objectives:**	• To describe the anti-HA antibody response against A/Vietnam and potential other clade using an enzyme linked immunosorbent assay (ELISA) test in a subset of 80 subjects in each formulation group (40 subjects per age sub-group) for subjects who receive A/Vietnam in the primary vaccination series only, before the first vaccination and 21 days after the second vaccination (*Amendment 1*).<br>• To describe in a subset of 80 subjects in each formulation group (40 subjects per age sub-group) for subjects who receive A/Vietnam in the primary vaccination series and only at Center 4 the cellular mediated immune (CMI) response against A/Vietnam and potential other Clades before the first vaccination and 21 days after the second vaccination.*(Amendments 1, 2 and 3).*<br>• To describe in a subset of 80 subjects in each formulation group (40 subjects per age sub-group) for subjects who receive A/Vietnam in the primary vaccination series only, the anti-neuraminidase antibody response against A/Vietnam and potential other clades before the first vaccination (D0) and 21 days after the second vaccination *(Amendment 1).*
**Observational Endpoints:**	**Cellular mediated immune response**<br>Secretion of a panel of Th1 (IFN-gamma, TNF-alpha, IL-2), and Th2 (IL-5, IL-4, IL-13) cytokines by peripheral blood mononuclear cells (PBMCs), upon *in vitro* re-stimulation with vaccine antigens will be quantified before the first vaccination and 21 days after the second vaccination *(Amendment 3).*<br>**Neuraminidase inhibition test**<br>Anti-neuraminidase Ab titer on D0 and D42 in a subset of 80 subjects in each formulation group (for subjects who receive A/Vietnam in the primary vaccination series only) *(Amendment 1).*<br>• Anti-neuraminidase titer obtained on D0 and D42; and summarized at the subject level by individual geometric mean of duplicate at each timepoint. The following endpoints will be derived:<br>• Individual titer ratios D42/D0.<br>• 2- and 4-fold increase from D0 to D42.<br>**Anti HA response by ELISA**<br>Anti- HA antibody titers against the A/H5N1 strain will be expressed in duplicate at least on D0 and D42 in a subset of 80 subjects in each formulation group (for subjects who receive A/Vietnam in the primary vaccination series only) *(Amendment 1).*
**Methodology/Trial Design (*Amendment 1*):**	Open, randomized, multicenter trial. A total of 650 subjects will be included in the trial.<br>Of these, 600 subjects (300 subjects aged 18 to 60 years and 300 subjects aged

**Company:**	sanofi pasteur
**Investigational Product/ Drug Product:**	A/H5N1 inactivated, split-virion influenza vaccine made in embryonated eggs
**Active Substance(s):**	Inactivated influenza virus split with octoxinol 9

>60 years) will receive two vaccinations (at D0 and D21) of A/Vietnam as a primary series of either 30 µg HA with adjuvant or 7.5 µg HA without adjuvant.

A subset of these subjects (N=160, i.e. subjects included in Center 2) receiving A/Vietnam as the primary series will receive a booster vaccination of 7.5 µg HA (A/Vietnam) without adjuvant at M6.

A subset of 140 subjects, included in Center 1 and 3 and receiving the 30 µg HA with adjuvant formulation as primary series, will receive a booster vaccination at 22 months (with 30 µg HA with adjuvant of an A/Indonesia H5N1 vaccine strain).

The remaining subjects, i.e. subjects receiving the 7.5 µg HA without adjuvant as primary series in centers 1 and 3 and all subjects in center 4, regardless of the vaccine received in primary series, will receive no booster and stop the trial at M22

This schema is for subjects receiving the 7.5µg HA without adjuvant as primary series, *(Amendment 3).*

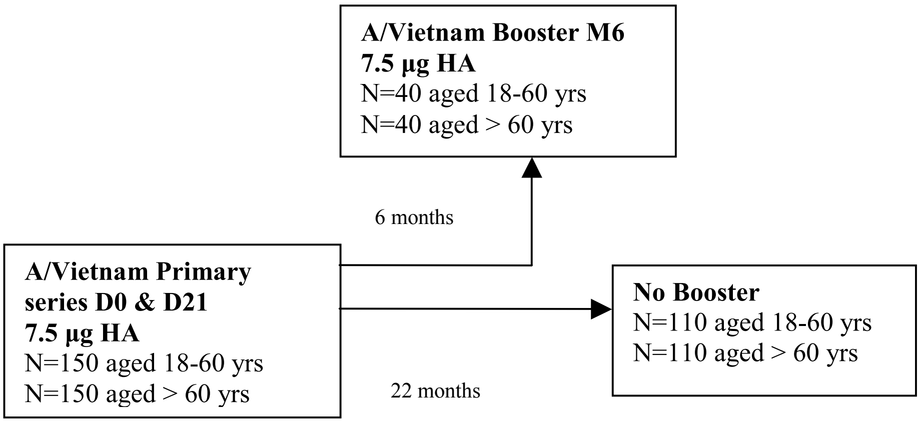

This schema is for subjects receiving the 30 µg HA with adjuvant as primary series *(Amendment 3)*

**Company:**	sanofi pasteur
**Investigational Product/ Drug Product:**	A/H5N1 inactivated, split-virion influenza vaccine made in embryonated eggs
**Active Substance(s):**	Inactivated influenza virus split with octoxinol 9

<table>
<tr><td></td><td>
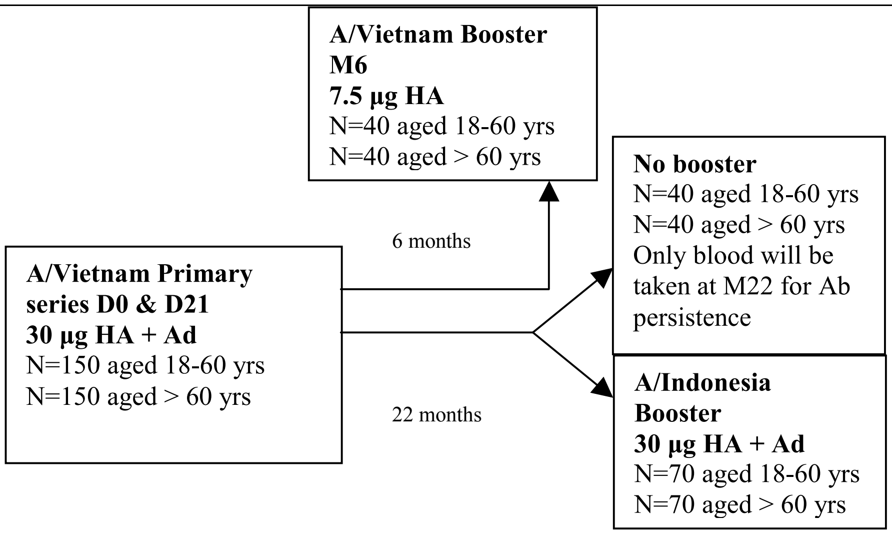

In addition, 50 subjects aged 18 to 60 years will be included in Center 2 at the time of the A/Indonesia booster to receive two vaccinations (at D0 and D21) of A/Indonesia as a primary series of 30 µg HA with adjuvant (Amendment 3). These vaccinations will be performed in parallel with the A/Indonesia (Amendment 2) booster. The subjects receiving A/Indonesia as a primary series will receive no booster vaccination.</td></tr>
<tr><td>Planned Sample Size:</td><td>Total: 650 subjects. (Amendment 3).</td></tr>
<tr><td>Vaccination and Specimen Collection Schedules and Duration of Follow-up:</td><td>Subjects will receive two vaccinations of the investigational vaccine separated by 21 days and a booster at 6 or 22 months after primary vaccination series, as applicable (Amendment 3)

For all subjects, a 30 mL blood sample will be taken immediately prior to the first vaccination and at each subsequent visit for serology analyses. For subjects recruited at Center 4 only, an additional 20 mL blood sample will be taken in a subset of 80 subjects in each formulation group for CMI analysis at baseline, 21 days after the second vaccination (Amendment 3).</td></tr>
</table>

**Company:**	sanofi pasteur
**Investigational Product/ Drug Product:**	A/H5N1 inactivated, split-virion influenza vaccine made in embryonated eggs
**Active Substance(s):**	Inactivated influenza virus split with octoxinol 9

**Investigational Product:**	A/H5N1 inactivated, split-virion influenza vaccine made in embryonated eggs
**Form:**	Ready-to-use multidose vials containing 10 x 0.5 mL doses.
**Composition (*Amendment 1*):**	**Primary series:**<br>Two strains will be used *(Amendment 3)*:<br>H5N1 A/Vietnam: A/Vietnam/1194/2004/NIBRG-14 (H5N1)<br>H5N1 A/Indonesia: A/Indonesia/5/05-RG2 (H5N1)<br>For the A/Vietnam/1194/2004/NIBRG-14 (H5N1) strain, two formulations will be used:<br>- 30 µg HA with aluminum hydroxide adjuvant (600 µg Al^{3+}) per dose of 0.5 mL.<br>- 7.5 µg HA without adjuvant per dose of 0.5 mL<br>For the A/Indonesia/5/05-RG2 (H5N1) strain, one formulation will be used:<br>- 30 µg HA with aluminum hydroxide as adjuvant (600 µg Al^{3+}) per dose of 0.5 mL.<br>*Note: vaccines contain 45 µg of Thiomersal per dose*<br>**Booster vaccination at 6 months** (*added in Amendment 1*):<br>One strain will be used:<br>H5N1 A/Vietnam: A/Vietnam/1194/2004/NIBRG-14 (H5N1)<br>One formulation will be used:<br>- 7.5 µg HA without adjuvant per dose of 0.5 mL<br>*Note: vaccines contain 45 µg of Thiomersal per dose*<br>**A/Indonesia (*Amendments 2 and 3*) booster vaccination** (*added in Amendment 1*):<br>One strain will be used:<br>A/Indonesia: A/Indonesia/5/05-RG2 (H5N1) per dose of 0.5 mL<br>One formulation will be used:<br>- 30 µg HA with aluminum hydroxide as adjuvant (600µg Al^{3+}) per dose of 0.5 mL<br><br>*Note: vaccines contain 45 µg of Thiomersal per dose*
**Route:**	IM injection into the deltoid muscle
**Batch Numbers (*Amendment 1*):**	A/Vietnam: [redacted] (30 µg HA with aluminum hydroxide as adjuvant)<br>[redacted] (7.5 µg HA without adjuvant)<br>A/Indonesia: [redacted] (30 µg HA with aluminum hydroxide as adjuvant).
**Control Product:**	None.
**Inclusion criteria:**	1) Aged over 18 years on the day of inclusion, and 18 to 60 years for the 50 additional subjects for A/Indonesia primary series (Amendment 3).<br>2) Informed consent form signed.<br>3) Able to attend all scheduled visits and to comply with all trial procedures.<br>4) For a woman, inability to bear a child or negative urine pregnancy test.<br>5) For a woman of child-bearing potential, use of an effective method of

**Company:**	sanofi pasteur
**Investigational Product/ Drug Product:**	A/H5N1 inactivated, split-virion influenza vaccine made in embryonated eggs
**Active Substance(s):**	Inactivated influenza virus split with octoxinol 9

	contraception or abstinence for at least 4 weeks prior and at least 4 weeks after to each vaccination.<br>6) Addendum 1 of the informed consent form signed and dated by the subject (this inclusion criteria has to be checked only at V04 for subjects who are to receive a booster at M6 or later) (*Amendment 1*).<br>7) Addendum 2 of the informed consent form signed and dated by the subject (this inclusion criteria has to be checked only at V04_Add for subjects who are to receive a A/Indonesia booster (Amendment 2).<br>8) Addendum 3 of the Informed Consent Form signed and dated by the subject (this inclusion criterion has to be checked only at V05 for subjects who are to receive a A/Indonesia booster) *(Amendment 3)*.
**Exclusion criteria:**	1) Systemic hypersensitivity to any component of the vaccine or a life-threatening reaction after previous administration of a vaccine containing the same substances (egg proteins, chick proteins, thimerosal, aluminum, neomycin, formaldehyde, and octoxinol 9).<br>2) Febrile illness (oral temperature ≥37.5°C) on the day of inclusion.<br>3) Breast-feeding.<br>4) Previous vaccination with an avian flu vaccine.<br>5) Participation in a clinical trial (drug, device, or medical procedure) within 4 weeks prior to the first vaccination.<br>6) Planned participation in another clinical trial during the present trial period.<br>7) Congenital or acquired immunodeficiency, or receipt of immunosuppressive therapy such as anti-cancer chemotherapy or radiation therapy within the preceding 6 months or long-term systemic corticosteroid therapy.<br>8) Chronic illness that could interfere with trial conduct or completion (e.g. cardiac, renal, diabetes, or auto-immune disorders).<br>9) Current alcohol or drug abuse that may interfere with the subject's ability to comply with trial procedures.<br>10) Receipt of blood or blood-derived products within the past 3 months.<br>11) Any vaccination within 4 weeks prior to the first trial vaccination.<br>12) Vaccination planned within 4 weeks after any trial vaccination.<br>13) Thrombocytopenia or bleeding disorder contraindicating intramuscular vaccination.<br>14) Subject deprived of freedom by an administrative or court order, or in an emergency setting, or hospitalized without his/her consent.
**Statistical methods (*Amendments 1 and 2*):**	A first statistical analysis will be performed on all results obtained 21 days after the second vaccination, addressing the safety and immunogenicity objectives.<br>A second statistical analysis will be performed when all the data obtained 21 days after the M6 booster have been locked (second partial database lock) in order to address the

**Company:**	sanofi pasteur
**Investigational Product/ Drug Product:**	A/H5N1 inactivated, split-virion influenza vaccine made in embryonated eggs
**Active Substance(s):**	Inactivated influenza virus split with octoxinol 9

	immunogenicity and safety objectives for the A/Vietnam booster vaccination and the antibody persistence objective.<br>A third statistical analysis will be performed 21 days after the A/Indonesia booster vaccination to address the immunogenicity and safety endpoints for the A/Indonesia booster , the Ab persistence endpoints, and the primary series A/Indonesia vaccination for the additional 50 subjects included in Protocol Amendment 3.<br>A final statistical analysis will be produced after each 6 month safety follow-up of both booster vaccinations and of the primary series vaccinations of the additional subjects.<br>All four main analyses will be descriptive; for the main parameters, 95% CIs of point estimates will be calculated using normal approximation for quantitative data and exact binomial distribution (Clopper-Pearson method) for proportions.<br>Additionally, for each injection, the safety data will be presented according to the EMEA safety criteria.<br>**Sample size**<br>A total sample size of 300 subjects per primary series formulation group for the A/Vietnam strain will provide a probability of 95% to observe a 1% incidence for any adverse event (AE) in any sub-group; and a probability of 78% to observe a 1% incidence of any AE in each sub-group.<br>For the additional subjects receiving a primary vaccination series of A/Indonesia, the sample size was arbitrarily set to 50 subjects (Amendment 3).

Figure 1: Trial Flow-chart for Subjects Receiving the Six-month Booster (Center 2)

Visit	V01	V02	V03	V04	V05	6-month follow-up*
**Visit interval**	VAC1	VAC2 = VAC1+21D	VAC2+21D	VAC3 = VAC1+180D	VAC3+21D	Last VAC+180D
**Indicative Days (D)**	**D0**	**D21±3D**	**VAC2+ 21D±3D**	**D180±8D**	**VAC3+ 21D±3D**	**Last VAC+ D180+15D**
**Indicative Months (M)**				**M6**		
**Informed consent signed**	√					
**Addendum 1 to informed consent form (*added in Amendment 1*)**				√		
**Demography**	√					
**Urine pregnancy test†**	√	√		√		
**Physical examination**	√	√	√	√	√	√‡
**Influenza-like illness and flu vaccination history (*Amendment 1*)**				√		
**Past and current significant medical history**	√					
**Inclusion & exclusion criteria**	√			√		
**Randomization**	√					
**Temporary and definite contraindications**		√		√		
**Blood sampling for serology (30 mL)**	√<br>BL1	√<br>BL2	√<br>BL3	√<br>BL4	√<br>BL5	
**Vaccine injection**	√<br>VAC1§	√<br>VAC2§		√<br>VAC3§		
**30-minute observation period**	√	√		√		
**Diary Card (DC)/Memory Aid (MA):**<br>Provided:<br>Collected:	DC1	DC2<br>DC1	MA1<br>DC2	DC3<br>MA1	MA2<br>DC3	MA2**
**Collection of injection site reactions**	√	√	√		√	
**Collection of systemic events/reactions**	√	√	√		√	
**Concomitant medications**	√	√	√	√	√	
**Termination record**			√		√	
**Serious Adverse Events**	**Collected through the whole study period**					

* A telephone call or a visit at the site is to be arranged at least 6 months after the last vaccination to collect information on SAEs. In the event of an SAE, the Investigator may arrange a visit with the subject to obtain further information.

† For women of child-bearing potential only.

‡ Limited to medical interview if visit replaced by phone call.

§ Using A/Vietnam: A/Vietnam/1194/2004/NIBRG 14 (H5N1). (*Amendment 1*).

** MA2 to be returned by mail if visit replaced by 'phone call.

Figure 2: Trial Flow-Chart for Subjects Receiving the 22-month Booster (Centers 1 and 3)

**Visit**	**V01**	**V02**	**V03**	**V04**	**V04_ Add**	**V05**	**V05_ Add**	**V06**	**6-month follow-up***
**Visit interval**	**VAC1**	**VAC2 = VAC1+ 21D**	**VAC2 +21D**	**VAC1 +180D**	**VAC1 +450D**	**VAC3 = VAC1+ 670D**	**VAC3 +7D**	**VAC3 +21D**	**VAC3 +180D**
**Indicative Days (D)**	**D0**	**D21±3D**	**VAC2+ 21D±3D**	**D180 ±8D**	**D450 +15D**	**D670± 15D**	**VAC3+ 7D±1D**	**VAC3+ 21D±3D**	**VAC3+ 180D+15D**
**Indicative Months (M)**				**M6**	**M15**	**M22**			**M28**
**Informed consent signed**	√								
**Addendum 1 to informed consent form (*added in Amendment 1*)**				√					
**Addendum 2 to informed consent form (*added in Amendment 2*)**					√				
**Addendum 3 to informed consent form (*added in Amendment 3*)**						√			
**Demography**	√								
**Urine pregnancy test†**	√	√				√			
**Physical examination**	√	√	√	√	√	√	√	√	√‡
**Influenza-like illness and flu vaccination history (*Amendment 1*)**				√		√			
**Past and current significant medical history**	√								
**Inclusion & exclusion criteria**	√			√	√	√			
**Randomization**	√								
**Temporary and definite contraindications**		√				√			
**Blood sampling for serology (30 mL)**	√ BL1	√ BL2	√ BL3	√ BL4	√ BL5	√ BL6	√ BL7	√ BL8	
**Vaccine injection**	√ VAC1§	√ VAC2§				√ VAC3 **			
**30-minute observation period**	√	√				√			
**Diary Card (DC)/ Memory Aid (MA): Provided: Collected:**	DC1	DC2 DC1	MA1 DC2	MA2 MA1	MA3 MA2	DC3 MA3		MA4 DC3	MA4††
**Collection of injection site reactions**	√	√	√			√		√	
**Collection of systemic events/reactions**	√	√	√			√		√	
**Concomitant medications**	√	√	√			√		√	

Visit	V01	V02	V03	V04	V04_ Add	V05	V05_ Add	V06	6-month follow-up*
**Visit interval**	**VAC1**	**VAC2 = VAC1+ 21D**	**VAC2 +21D**	**VAC1 +180D**	**VAC1 +450D**	**VAC3 = VAC1+ 670D**	**VAC3 +7D**	**VAC3 +21D**	**VAC3 +180D**
**Indicative Days (D)**	**D0**	**D21±3D**	**VAC2+ 21D±3D**	**D180 ±8D**	**D450 +15D**	**D670± 15D**	**VAC3+ 7D±1D**	**VAC3+ 21D±3D**	**VAC3+ 180D+15D**
**Indicative Months (M)**				**M6**	**M15**	**M22**			**M28**
**Termination record**			√	√	√			√	
**Serious Adverse Events**	**Collected through the entire study period**								

* A telephone call or a visit at the site is to be arranged at least 6 months after the last vaccination to collect information on SAEs. In the event of an SAE, the Investigator may arrange a visit with the subject to obtain further information.

† For women of child-bearing potential only.

‡ Limited to medical interview if visit replaced by phone call.

§ Using A/Vietnam: A/Vietnam/1194/2004/NIBRG-14 (H5N1). (*Amendment 1*).

** Using A/Indonesia: A/Indonesia/5/05-RG2 (H5N1) (*Amendmenst 1 and 3*).

†† MA4 (*Amendment 2*) to be returned by mail if visit replaced by ‘phone call.

Figure 3: Trial Flow-Chart for Subjects Receiving A/Vietnam as Primary Vaccination and No Booster (Subjects Primed with the 7.5 µg HA without Adjuvant in Centers 1 and 3 and All Subjects in Center 4)

Visit	V01	V02	V03	V04	V04_ Add	V05*
Visit interval	VAC1	VAC2 = VAC1+ 21D	VAC2 +21D	VAC1 +180D	VAC1 +450D	VAC1+ 670D
Indicative Days (D)	D0	D21±3D	VAC2+ 21D±3D	D180 ±8D	D450 +15D	D670± 3D
Indicative Months (M)				M6	M15	M22
Informed consent signed	√					
Addendum 1 to informed consent form (*added in Amendment 1*)				√		
Addendum 2 to informed consent form (*added in Amendment 2*)					√	
Demography	√					
Urine pregnancy test†	√	√				
Physical examination	√	√	√	√	√	√
Influenza-like illness and flu vaccination history (*Amendment 1*)				√		√
Past and current significant medical history	√					
Inclusion & exclusion criteria	√			√		
Randomization	√					
Temporary and definite contraindications		√				
Blood sampling for serology (30 mL)	√ BL1	√ BL2	√ BL3	√ BL4	√ BL5	√ BL6‡
Blood sampling for CMI analysis (20 mL)§	√ BL1		√ BL3			
Vaccine injection	√ VAC1**	√ VAC2**				
30-minute observation period	√	√				
Diary Card (DC)/ Memory Aid (MA): Provided:	DC1	DC2	MA1	MA2	MA3	
Collected:		DC1	DC2	MA1	MA2	MA3
Collection of injection site reactions	√	√	√			
Collection of systemic events/reactions	√	√	√			
Concomitant medications	√	√	√			

**Visit**	**V01**	**V02**	**V03**	**V04**	**V04_ Add**	**V05***
**Visit interval**	**VAC1**	**VAC2 = VAC1+ 21D**	**VAC2 +21D**	**VAC1 +180D**	**VAC1 +450D**	**VAC1+ 670D**
**Indicative Days (D)**	**D0**	**D21±3D**	**VAC2+ 21D±3D**	**D180 ±8D**	**D450 +15D**	**D670± 3D**
**Indicative Months (M)**				**M6**	**M15**	**M22**
**Termination record**			√	√		√
**Serious Adverse Events**	**Collected throughout the entire study period**					

* For subjects receiving the 7.5 µg HA without adjuvant for primary vaccination series a telephone call or a visit at the site is to be arranged to collect information on SAEs. In the event of an SAE, the Investigator may arrange a visit with the subject to obtain further information.

† For women of child-bearing potential only.

‡ Only for subjects receiving the 30 µg HA with adjuvant for primary vaccination series

§ In a sub-group of 80 subjects per formulation group. Only applicable for Center 4 [redacted] Belgium).

** Using A/Vietnam: A/Vietnam/1194/2004/NIBRG-14 (H5N1). (*Amendment 1*).

Figure 4: Trial Flow-chart for Additional Subjects Receiving A/Indonesia as Primary Vaccinations (Center 2)

Visit	V01	V02	V03	6-month follow-up*
Visit interval	VAC1	VAC2 = VAC1+21D	VAC2+21D	Last VAC+180D
Indicative Days (D)	D0	D21±3D	VAC2+ 21D±3D	Last VAC + 180D+15D
Indicative Months (M)				
Informed consent signed	√			
Demography	√			
Urine pregnancy test†	√	√		
Physical examination	√	√	√	√‡
Influenza-like illness and flu vaccination history	√			
Past and current significant medical history	√			
Inclusion & exclusion criteria	√			
Temporary and definite contraindications		√		
Blood sampling for serology (30 mL)	√ BL1	√ BL2	√ BL3	
Vaccine injection	√ VAC1§	√ VAC2§		
30-minute observation period	√	√		
Diary Card (DC)/Memory Aid (MA): Provided: Collected:	DC1	DC2 DC1	MA1 DC2	MA1
Collection of injection site reactions	√	√	√	
Collection of systemic events/reactions	√	√	√	
Concomitant medications	√	√	√	
Termination record			√	
Serious Adverse Events	Collected through the whole study period			

* A telephone call or a visit at the site is to be arranged at least 6 months after the last vaccination to collect information on SAEs. In the event of an SAE, the Investigator may arrange a visit with the subject to obtain further information.

† For women of child-bearing potential only.

‡ Limited to medical interview if visit replaced by phone call.

§ Using A/Indonesia: A/Indonesia/5/05-RG2 (H5N1) *(Amendment 3)*

Table of Contents

Synopsis **2**

List of Abbreviations **22**

1 Introduction **24**

1.1 Background 24

1.2 Subject Benefits / Potential Risks 26

1.3 Rationale 27

2 Trial Objectives **32**

2.1 Objectives 32
2.1.1 Endpoints 32

2.2 Observational Objectives 34

3 Investigators and Trial Organization **35**

4 Independent Ethics Committee (IEC)/Institutional Review Board (IRB) **35**

5 Investigational Plan **35**

5.1 Description of the Overall Trial Design and Plan 35
5.1.1 Trial Design (*Amendments 1, 2 and 3*) 35
5.1.2 Trial Plan 38
5.1.3 Visit Procedures 40
5.1.4 Planned Trial Calendar 49

5.2 Discussion of Trial Design 50

5.3 Selection of Trial Population 51
5.3.1 Recruitment Procedures 51
5.3.1.1 Belgian Sites 51
5.3.1.2 UK Site 51
5.3.2 Participant Information and Consent 51
5.3.3 Inclusion Criteria 52
5.3.4 Exclusion Criteria 52
5.3.5 Temporary Contraindications 53
5.3.6 Removal of Participants from Treatment or Assessment 54
5.3.6.1 Conditions for Withdrawal 54
5.3.6.2 Lost to Follow-up Procedures 54
5.3.6.3 Termination Classification 55
5.3.6.4 Follow-up of Discontinuations 55

5.3.6.5 Follow-up and Reporting of Pregnancies 55
5.3.7 Medical History 55
5.4 Modification of the Trial and Protocol 56
5.5 Interruption of the Trial 56
6 Treatments 56
6.1 Vaccines Administered (*Amendments 1, 2 and 3*) 56
6.2 Identity of Investigational Product 57
6.2.1 Composition 57
6.2.2 Preparation and Administration 57
6.2.3 Precautions for Use 57
6.2.4 Dose Selection and Timing 58
6.3 Identity of Control Product 58
6.4 Identity of Other Products 58
6.5 Product Logistics 58
6.5.1 Labeling and Packaging 58
6.5.2 Storage and Shipment Conditions 58
6.5.3 Product Accountability 59
6.5.4 Replacement Doses 59
6.5.5 Return of Unused Products 59
6.6 Randomization/Allocation Procedures (*Amendments 1 and 3*) 59
6.7 Blinding and Code Breaking Procedures 60
6.8 Concomitant Therapy 60
6.9 Treatment Compliance 61
7 Specimens and Clinical Supplies 61
7.1 Management of Samples for Humoral Immune Response 61
7.1.1 Sample Collection for Serological Assessments 61
7.1.2 Sera Preparation of Blood Samples for Serological Assays 62
7.1.3 Sera Storage and Shipment of Samples for Serological Assays (*Amendment 1*) 62
7.1.4 Retention of Unused Serum Samples for Repository (Humoral) (*Amendment 1*) 63
7.2 Management of Samples for Cellular Mediated Immune Response 63
7.2.1 Sample Collection for Cellular Mediated Immune Response Assessments 63
7.2.2 Peripheral Blood Mononuclear Cell Purification for Cellular Mediated Immune Response Assessment 64
7.2.3 Peripheral Blood Mononuclear Cell Storage and Shipment of Samples for Cellular Mediated Immune Response Assessment 64
7.3 Clinical Supplies 64

8 Assessments Methods and Endpoints 65
8.1 Immunogenicity Endpoints and Assessments Methods 65
8.1.1 Endpoints 65
8.1.2 Observational Endpoints 66
8.1.3 Assessment Methods 67
8.1.3.1 Hemagglutination Inhibition Test 67
8.1.3.2 Seroneutralization 68
8.1.3.3 Cellular Mediated Immune Response 68
8.1.3.4 Neuraminidase Inhibition Test 68
8.1.3.5 ELISA Tests 68
8.1.4 Handling of Missing Data and Outliers 69
8.2 Efficacy Endpoints and Assessments Methods 69
8.3 Safety Endpoints and Assessments Methods 69
8.3.1 Definitions 69
8.3.2 Endpoints 71
8.3.3 Safety Assessment Methods 72
8.3.3.1 30-minute Observation Period 72
8.3.3.2 Reactogenicity (Solicited Reactions from D0 to D7 after each Vaccination) . 72
8.3.3.3 Adverse Events from D0 to D21 after each Vaccination 76
8.3.3.4 Transcription of Safety Information to the CRF; Causality Assessment 76
8.3.4 Handling of Missing Data and Outliers 77
9 Serious Adverse Events 78
9.1 Reporting of Serious Adverse Events 78
9.1.1 Initial Reporting by the Investigator (*Amendment 1*) 78
9.1.2 Follow-up Reporting by the Investigator (*Amendment 1*) 78
9.1.3 Reporting of SAEs Occurring After Subject Trial Termination 79
9.1.4 Causal Relationship 79
9.1.5 Reporting SAEs to Health Authorities and IECs/IRBs 79
9.1.6 Additional Information 79
10 Data Collection and Management 80
10.1 Data Collection, CRF Completion 80
10.2 Data Management 80
11 Statistical Methods and Determination of Sample Size 81
11.1 Statistical Methods 82
11.1.1 Hypotheses and Statistical Methods for the Safety Endpoints 82
11.1.1.1 Hypotheses 82
11.1.1.2 Safety Analysis 82
11.1.2 Hypotheses and Statistical Methods for the Immunogenicity Endpoints 83

11.1.2.1 Hypotheses ... 83
11.1.2.2 Statistical Analysis ... 83
11.1.2.2.1 EMEA Immunogenecity Criteria for Analysis ... 83
11.1.2.2.2 Methods for Statistical Analysis ... 84
11.1.2.2.3 Exploratory Statistical Analyses ... 85
11.1.2.2.4 Immunogenicity Observational Endpoints ... 85
11.2 Population to be Analyzed ... 86
11.2.1 Definition of Populations ... 86
11.2.1.1 Full Analysis Set ... 86
11.2.1.2 Per-Protocol Analysis Set ... 87
11.2.1.3 Safety Analysis Set ... 87
11.2.1.3.1 Cellular Mediated Immune Response and Additional Serological Tests ... 88
11.2.2 Populations Used in Analyses ... 88
11.3 Determination of Sample Size and Power Calculation ... 89
11.4 Interim Analysis ... 89
12 Ethical and Legal Issues and Investigator/Sponsor Responsibilities ... 89
12.1 Ethical Conduct of the Trial / Good Clinical Practice ... 89
12.2 Source Documents and Source Data ... 90
12.3 Confidentiality of Data and Access to Subject Records ... 90
12.4 Monitoring, Auditing, and Archiving ... 90
12.4.1 Monitoring ... 90
12.4.2 Audits and Inspections ... 91
12.4.3 Archiving ... 91
12.5 Financial Contract and Insurance Coverage ... 92
12.6 Stipends for Participation ... 92
12.7 Publication Policy ... 92
13 Reference List ... 93

List of Abbreviations

Ab	Antibody
Ad	Adjuvant
ADR	Adverse Drug Reaction
AE	Adverse Event
AR	Adverse Reaction
CI	Confidence Interval
CIF	Complementary Information Form
CMI	Cellular Mediated Immune
CHMP former CPMP	Committee for Medicinal Products for Human Use
CRA	Clinical Research Associate
CRF	Case Report Form
CTM	Clinical Trial Manager
D	Day
DC	Diary Card
DCF	Data Clarification Form
°C	Degrees Celsius
dil	dilution
ELISA	Enzyme Linked Immunosorbent Assay
EMEA	European Medicines Agency
FAS	Full Analysis Set
FVFS	First Visit First Subject
FVLS	First Visit Last Subject
GCI	Global Clinical Immunology
GCP	Good Clinical Practice
GM	Geometric Mean
GMT	Geometric Mean Titer
GMTR	Geometric mean of individual titer ratio
HA	Hemagglutinin
HI	Hemagglutination inhibition
ICH	International Conference on Harmonization
IEC	Independent Ethics Committee
IL	Interleukin
IM	Intramuscular
INFα	Interferon-alpha
IRB	Institutional Review Board

LLOQ	Lower limit of quantification
LVLS	Last Visit Last subject
M	Month
MA	Memory Aid
MedDRA	Medical Dictionary for Regulatory Activities
MDCK	Madin-Darby canine kidney
NIBSC	National Institute for Biological Standards and Control
NSAID	Non Steroidal Anti-Inflammatory Drugs
OD	Optical Density
PBMC	Peripheral blood mononuclear cell
PPAS	Per Protocol Analysis Set
QA	Quality Assurance
RBC	Red blood cell
SAE	Serious Adverse Event
SafAS	Safety Analysis Set
SN	Seroneutralization
SRH	Single Radial Hemolysis
TNFα	Tumor Necrosis Factor-alpha
UK	United Kingdom
ULOQ	Upper limit of quantification
USA	United States of America
V	Visit
VAC	Vaccination
WHO	World Health Organization

1 Introduction

1.1 Background

An influenza pandemic occurs when a novel influenza virus emerges against which the vast majority of the world's population has no immunity. This has been observed only with influenza A viruses and is due to the emergence of a new antigenic variant (antigenic shift) caused by substitution of the hemagglutinin antigen on the surface of the virus, with or without a concomitant change in neuraminidase, the other surface antigen. If such a virus demonstrates the ability to transmit efficiently from person to person, the result is a global outbreak of the disease that affects a high percentage of individuals in a short period of time and is likely to cause substantially increased morbidity and mortality in all countries of the world.

The first identifiable influenza pandemic in more than 300 years of detailed records of human influenza occurred in 1847 (1). Since 1847, three influenza pandemics have caused millions of deaths worldwide, social disruption and profound economic losses (2):

- The "Spanish influenza", between 1918-1919, was due to an A/H1N1 virus related to porcine influenza.
- The "Asian influenza", between 1957-1958, was due to an A/H2N2 virus.
- The "Hong Kong influenza", between 1968-1969, was due to an A/H3N2 virus.

The impact of pandemic influenza is better appreciated when compared with the more familiar patterns associated with inter-pandemic disease. Between pandemics, influenza is characterized by extremely low viral transmission in the summer (3) followed by an annual increase in winter seasonal activity (4). The winter epidemic is variable in intensity and duration, but usually produces clinically recognizable disease in the population for 8 to 12 weeks.

In contrast, influenza pandemics are not limited to the winter season, and are characterized by several waves of infection following the emergence of the virus (2). In the 1918-19 pandemic, the first wave occurred in Spring 1918 in the United States of America (USA). The second began in August 1918 and had a higher mortality rate. The third appeared in Spring 1919. The reason for these waves of infection is unclear. During the successive waves, virus virulence increased (2). These characteristics have important implications for planning against the next pandemic.

The pandemics of the 20th century occurred at intervals ranging from 11 to 39 years. It is now 39 years since the last pandemic in 1968. Pandemic influenza can occur at any time of year and may spread rapidly through the world. The three influenza pandemics of the 20th century indicate what can be expected when the next one occurs.

The estimated clinical attack rate was remarkably similar in the last three pandemics, being about 25%. The 1918-19 pandemic killed 50 to 100 million people versus around 1 million people in 1957-58 and 800 000 people in 1968-69.

Between pandemics, the vast majority of influenza-related deaths occur in the elderly, although infants and young children are also affected. A similar pattern of age-specific mortality occurred

in the first wave of 1918 pandemic influenza, however, during the second wave, this pattern changed radically. Mortality among 0 to 4 year-olds increased considerably, but in all other age groups less than 40 years mortality increased more dramatically, peaking at almost 15% in those aged 25 to 29 years. In contrast, in those over 50 years, death rates were lower in the second wave than in the first, and were especially low in those over 80 years (2).

Pandemic influenza is characterized by the sudden onset of severe typical influenza symptoms, such as high fever, headache, myalgia, arthralgia, anorexia, nausea, vomiting and cough, lasting 2 to 4 days. Although most patients recover, some die rapidly due to tracheo-bronchitis associated with dyspnoea. After initial recovery, some patients subsequently develop pneumonia.

It is likely that the next pandemic virus could emerge in China and could include surface antigens or virulence factors derived from animal influenza viruses, much like the avian A/H5N1 virus that emerged in Hong Kong in 1997.

Avian influenza is a contagious disease caused by viruses that normally infect only birds and less commonly, pigs. An outbreak of avian influenza, especially of the highly pathogenic form can be devastating for the poultry and farming industry. The disease can spread from country to country through migratory birds, including wild waterfowl, sea birds, and shore birds.

Since mid-December 2003, a growing number of Asian countries have reported outbreaks of highly pathogenic avian influenza in chickens and ducks resulting in the death or culling of more than 100 million poultry. Infections in several species of wild birds and in pigs have also been reported. A highly pathogenic strain A/H5N1 was the cause of most of these outbreaks.

In addition, A/H5N1 viruses have repeatedly “jumped the species barrier”, and caused at least 230 confirmed cases (including 132 deaths) in human adults and children in Vietnam, Thailand, Indonesia, China, Cambodia, Turkey, Iraq Azerbaijan, Djibouti and Egypt between December 2003 and mid-July.

Until recently, people who have been infected with the A/H5N1 virus have been in direct contact with infected and/or sick birds. However, a bird-flu outbreak in an Indonesian village where seven family members have now died, combined with the fact that the virus may have spread directly from a woman to her nephew and then from the nephew to his father, has raised the level of concern that the virus may be able to pass directly between people. With no animal identified as yet as the source of infection, the family cluster in Indonesia raises the suspicion of human-to-human transmission. This would be the first known three-person chain of human-to-human transmission. The virus has not spread beyond the members of this single extended family.

There is a possibility that in this current situation, avian and human influenza viruses could exchange genes if an individual was simultaneously infected with viruses from both species. This could give rise to a new subtype of the influenza virus to which humans would not have natural immunity, and could result in another influenza pandemic in humans.

Although antiviral drugs exist, vaccines will form the main prophylactic measure against pandemic influenza and will play an important role in the plans to prepare for a pandemic. The World Health Organization (WHO) Influenza Surveillance Program provides representative influenza viruses for antigenic and genetic analysis and from this information, the WHO is able to make recommendations on vaccine composition (5). The WHO also has a key-role to play in

detecting new influenza viruses that are likely to cause pandemics, and in advising on suitable vaccines strains and their use.

Conventional inactivated influenza vaccines will be unsuitable against pandemic influenza. Poor immunogenicity is expected in naïve adult populations, and recent studies of "pandemic like" vaccines have shown that vaccine-adjuvanation and a two-dose schedule can improve the immune response, especially in unprimed individuals (6) (7) (8) (9) (10) (11) (12).

In order to accelerate the development of the pandemic vaccine, the European Medicines Agency (EMEA) has developed guidelines for licensing pandemic influenza vaccines (13) (14). The guidelines recommend the development of a "mock-up" pandemic vaccine or "prototype pandemic influenza vaccine" which should be produced in the same way, have the same antigen content and same adjuvant system (if used) and use the same administration route as the intended pandemic vaccine during the inter-pandemic period. The licensing procedure of this "mock up" vaccine involves the submission and approval of a core pandemic dossier.

In the event of a pandemic with a new viral strain, a fast-track procedure for registration of the final pandemic vaccine will be implemented, based on the submission of a dossier for the pandemic variation.

1.2 Subject Benefits / Potential Risks

There are no definite benefits following vaccination in this trial. Subjects may develop an antibody response to the A/Vietnam/1194/2004/NIBRG-14 (H5N1) vaccine and the new vaccine strain (A/Indonesia/5/2005/RG2 [H5N1]), although this can not be guaranteed. Furthermore, in the event that an H5 strain is found in birds or circulates as a pandemic, cross-protection of such an antibody response cannot be guaranteed.

The potential risks of receiving the investigational vaccine are listed in the current version of the Investigator's Brochure. No safety signal was detected based on a consideration of all adverse events from the GPA01 and GPA02 studies by system organ class after primary and booster vaccinations.

In both GPA01 and GPA02, the incidence of adverse events following the second vaccination was lower than the incidence following the first vaccination, regardless of the formulation of the vaccine. This is an effect that is commonly observed in clinical studies involving more than one vaccination. In addition, in GPA02, the overall incidence of adverse events was slightly lower in the elderly population compared to adult subjects aged 18 to 60 years; such an observation is common in seasonal influenza clinical trials. These effects were not considered to be clinically important and overall, in all age groups and dosage/formulation groups, the vaccine was considered to be well tolerated.

There were no findings of clinical concern in the solicited injection site and solicited systemic reactions within 7 days of any vaccination. According to results observed with the 30 µg HA with adjuvant vaccine, the most frequently observed local reactions in a previous study, GPA01 (12) and in the present study, were pain, erythema and induration with an incidence >10%and the most frequently reported solicited systemic reactions in all groups and for each injection were malaise, headache and myalgia which were very common, and shivering and fever (oral temperature ≥37.5°C) which were common. Most local and systemic reactions generally appeared

on the day following vaccination and spontaneously disappeared within 3 days. Additionally, blood sampling can cause pain and bruising at the puncture site.
Data from interpandemic influenza vaccines suggest that other effects may include: neuralgia, paresthesia, convulsions, transient thrombocytopenia, or transient lymphadenopathy. In rare cases, urticaria, pruritus, erythematous rash, dyspnea, leading to shock and angioedema have been reported. Vasculitis with transient renal involvement has been reported in very rare cases. Rarely, neurological disorders, such as encephalomyelitis, neuritis and Guillain-Barré syndrome, have been reported.

Aluminum in the form of aluminum hydroxide has been used as an adjuvant in many licensed vaccines. Aluminum has a demonstrated safety profile (over six decades), but has been associated with local reactions such as erythema, subcutaneous nodules and contact hypersensitivity.

Due to the multidose presentation of the vaccine, thiomersal as preservative is also contained in the vaccines used in this study. Although there is no evidence of harm due to the presence thiomersal in the vaccines, there is a risk of hypersensitivity (allergic) reactions (EMEA public statement of thiomersal in vaccines for human use EMEA/CPMP/VEG/1194/04).

The safety profile obtained after the 6- and the 12-month booster vaccination, regardless of the booster dose, was similar to the one obtained after primary series, with no safety signals being raised.

The data from the first 6 months of the present study do not indicate any unexpected risks following administration of the investigational vaccine.

For subjects receiving the booster vaccination at 6 months, a seasonal flu vaccination could be received at least 4 weeks before. In this case, the Sponsor will provide the vaccine.

If the A/Indonesia booster is administered during seasonal flu period, a seasonal flu vaccine could be provided by the Sponsor in order to ensure that subjects could receive it in the time window allowed by the protocol.

1.3 Rationale

For the purpose of the core pandemic dossier (as described earlier and defined in the EMEA guidelines [13] [14]), clinical data must be obtained solely from healthy adults of various age groups. A two-study approach is suggested for the EMEA approval. According to the EMEA guidelines, a first, formulation/dosage-finding study, is to be conducted to determine the preferred dose of antigen, the necessity for an adjuvant and the schedule required to meet the agreed acceptance criteria for existing vaccines (1). Having established the dose, adjuvant and schedule in the first study, a second, larger study should be conducted primarily to establish a sufficiently large safety database to be able to detect an adverse event (AE) occurring at a frequency of 1% with a probability equal to 95%.

In view of the occurrence of human H5 virus infection with the highly pathogenic A/Vietnam/1194/2004 strain in recent years, this has been identified as a potential pandemic strain. Therefore, an H5N1 reassortant derived from the A/Vietnam/1194/2004 strain has been produced by reverse genetic and chosen as the “mock up” pandemic vaccine. The license

manufacturing process for the "mock up" pandemic vaccine is the same as that used for the inter-pandemic vaccine (Vaxigrip®).

Based on the EMEA guideline, a dose/formulation finding study of different formulations of an intramuscular (IM) pandemic A/H5N1 inactivated, split virion influenza vaccine in adults was initiated, as described below.

In May 2005, a Phase I, multicenter, randomized, open study (GPA01 [12]) was initiated to test several adjuvanted and non-adjuvanted A/H5N1 vaccine formulations (7.5, 15 and 30 µg with or without aluminum hydroxide as an adjuvant) as a two-dose schedule in immunologically naïve adults aged 18 to 40 years. In this study, 300 subjects received two injections (on D0 and D21) of one of the six investigational products and are currently being followed-up for a total of one year. Immunogenicity 21 days after the first and the second vaccinations has been determined by hemagglutination inhibition (HI) using turkey (HIT) and horse (HIH) erythrocytes, single radial haemolysis (SRH) and seroneutralization (SN). Antibody (Ab) persistence was also evaluated 6 and 12 months after the first vaccination.

The vaccine appeared to be generally safe and well tolerated (see Section 1.2 for further details) following two administrations of each formulation/dose with solicited local and systemic reactions occurring at a similar frequency to that seen using the interpandemic trivalent inactivated influenza vaccine in terms of the EMEA safety criteria within 3 days post-vaccination.

In terms of the immune response, Ab to the vaccine strain (A/Vietnam/1194/2004/NIBRG14 [H5N1]) was most reliably detected by SN and HI (using horse erythrocytes) assays. Antibodies to the vaccine strain were not detected in baseline samples. For all groups in the study, benefit was seen in terms of immunogenicity with a two-dose regimen. In subjects administered with two doses of 30 µg HA with adjuvant, the seroconversion rate (pre-injection antibody titer <8 [1/dil] and post-injection antibody titer of ≥32 [1/dil] [Hemaglutination Inhibition assay (HI) using horse erythrocytes]) was 62.7%. In the other dose/formulation groups, an immune response was also observed with a minimum seroconversion rate of 20.0% in the subjects who received two injections of the 7.5 µg HA with adjuvant formulation. In subjects receiving 7.5 µg HA without adjuvant and 15 µg HA both with and without adjuvant, the seroconversion rate was approximately 40% after two injections. At least one of the three criteria required in the EMEA Note for Guidance (i.e. percentage of seroconversion or significant increase in anti-HA antibodies >40%, percentage of seroprotection >70%, and GMT>2.5) was met for three of the six formulations (GMT >2.5 observed in groups 30 µg HA with adjuvant, 30 µg HA and 7.5 µg HA) 21 days after the first injection and for four of six formulations (GMT >2.5 and percentage of seroconversion or significant increase in anti-HA antibodies >40% observed in groups 30 µg HA with adjuvant, 30 µg HA, 15 µg HA with adjuvant and 15 µg HA) 21 days after the second injection.

In conclusion, this previous clinical trial (12) showed that there is a positive immunologic response for a potential pandemic vaccine, in particular with the 30 µg HA with adjuvant formulation. In addition, as an immune response was observed in a number of subjects receiving the lowest dose, it is of interest from a dose sparing perspective to further investigate the 7.5 µg HA without adjuvant dose/formulation.

The aim of the present clinical trial is therefore to gather information regarding the safety and immunogenicity of the selected formulation/doses (7.5 µg HA without adjuvant and 30 µg HA

with adjuvant) in adults (18 to 60 years) and the elderly (>60 years) based on the results of the previous formulation/dose finding trial.

In the present Phase II, multicenter, open trial, a total of 600 subjects (in two vaccine formulation groups [30 µg HA with adjuvant and 7.5 µg HA without adjuvant] and with two age groups [18 to 60 years and >60 years] per vaccine formulation group) will be enrolled in order to be able to detect an AE occurring at a frequency of 1% with a probability equal to 95% in each vaccine formulation group. According to the schedule defined by the results from the formulation/dose finding study, each subject will receive two vaccinations of either 30 µg HA with adjuvant or 7.5 µg HA without adjuvant administered 21 days apart.

The immunogenicity response 21 days after each vaccination and Ab persistence until the booster vaccination at 6 or 12 months will be evaluated by HI, and SN as required by the EMEA guideline.

Booster vaccination at 6 or 12 months after the first vaccination will be performed. Previous influenza pandemics have spread in two to three waves over a period of 13 to 23 months, with the first wave being less intense than the second, which may occur 3 to 9 months later. A booster vaccination may be required to induce a sufficient quantity of Ab to ensure protection throughout the pandemic period (11).

Additionally, the induced cellular mediated immunity (CMI) profile and neuraminidase inhibition test after vaccination will be documented in a subset of subjects, as suggested by the EMEA guideline.

Rationale for Amendment 1:

The original approved protocol (Version 2.0, dated 16 February 2006) for the present trial stated that a booster vaccination could be performed at either 6 or 12 months after the first vaccination according to the antibody persistence results observed in the Phase I study (GPA01).

Antibody persistence data at 6 months are now available from Study GPA01 to facilitate the assessment of the requirement for, and timing of, a booster vaccination in the present trial.

These results show:

- Antibody levels decrease at 6 months after the first vaccination to a level similar to that seen 21 days after the first vaccination irrespective of the group (i.e. 7.5, 15 and 30 µg HA with or without aluminum hydroxide as an adjuvant).
- The highest percentage (18%) of subjects with an anti-HA antibody titer ≥32[a] (1/dil) is observed for the 7.5 µg and 30 µg + adjuvant groups.
- 30% of subjects have detectable antibody (anti-HA antibody titer >8 [1/dil]) at 6 months following the first vaccination of 30 µg + adjuvant compared with 38% and 68% 21 days after the first and second vaccinations, respectively.
- There is no marked adjuvant effect regarding the persistence of antibodies at 6 months.

[a] Seroprotective titre for the inter-pandemic vaccine using the HI method.

Because no correlate of protection currently exists for a pandemic vaccine, it is impossible to say if the antibody level at 6 months in subjects having received two administrations of pandemic vaccine 21 days apart would be clinically protective.

In this context, and according to Ab persistence results from Study GPA01, the Sponsor has decided to investigate the booster effect in subjects primed with one of two dose regimens (7.5 µg HA without adjuvant or 30 µg HA with adjuvant) to assess the priming effect and immune memory regarding two objectives:

Effect of the timing of the booster

A first subset of subjects (N=160, 40 subjects per age group and per formulation received in the priming vaccination) will receive a booster vaccination with low dosage (7.5 µg HA without adjuvant) of the same vaccine strain as those used in the priming vaccination (H5N1 A/Vietnam: A/Vietnam/1194/2004/NIBRG-14 (H5N1) – henceforth referred to as A/Vietnam) at 6 months after the first vaccination.

Effect of the vaccine strain

A second subset of subjects (N=440, 110 subjects per age group and per formulation received in the priming vaccination) will receive a booster vaccination of either 7.5 µg HA without adjuvant or 30 µg HA with adjuvant of the A/Indonesia strain (referred to as the A/Indonesia booster) (Amendment 2)

In parallel, 100 additional subjects aged from 18 to 60 years will be enrolled to receive two administrations of the new vaccine strain (A/Indonesia) 21 days apart in order to assess the priming immunogenicity response to this strain.

Rationale for Amendment 2

The original approved protocol (Version 2.0, dated 16 February 2006) for the present trial stated that a booster vaccination could be performed at either 6 or 12 months after the first vaccination.

Protocol Amendment 1 (version 3.0, dated 06 September 2006), described the administration of the booster at 12 months with a new vaccine clade (A/Indonesia).

Due to difficulties in obtaining the reagents to quantify the HA in the vaccine, the release of the A/Indonesia vaccine has been delayed. As a result, the timing of the 12 month booster has been delayed and it is not possible currently to indicate an exact time for this booster vaccination as the release date of the vaccine is not yet defined. The time of administration of the booster vaccination is therefore referred to as 'to be determined' (TBD) throughout this protocol amendment.

Because of the unplanned delay in the booster vaccination, the study design has been modified so that a visit will take place at M15 (an 'additional V04' or 'V04_Add') in order to obtain a blood sample for the assessment of antibody persistence (in accordance with EMEA guidance (13)) although no booster will be administered at this visit. The original V05 therefore remains as V05, but the timing of this visit is TBD, with the remaining visits (V06 and the 6-month safety follow-

up visit[a]) being maintained at the original timing after the booster visit (21 days and 6 months, respectively).

In order to clarify the terms 'adult' and 'elderly', the study objectives have been slightly re-worded.

Rationale for Amendment 3:

The aim of this amendment is to specify the timing of the A/Indonesia booster, which will now be at 22 months after the first vaccination, and to describe some other changes in the design.

In GPA01, the administration of a 12 month A/Vietnam booster with 7.5 µg HA without adjuvant or 30 µg HA with adjuvant induced an immune response, which was higher in subjects vaccinated with an adjuvanted vaccine in the primary series than in the other subjects. In addition, the 30µg HA with adjuvant booster formulation induced a higher antibody response than the 7.5 µg HA without adjuvant booster.

In the present study, the 6-month A/Vietnam booster, containing 7.5 µg of HA without adjuvant and administered in a subset of primed subjects with either 7.5 µg HA without adjuvant or 30 µg HA with adjuvant, induced a limited increase in antibody titers and in the proportion of subjects with a detectable antibody titer.

Further results are provided in the current version of the Investigator's Brochure (version 8.0, dated December 2007).

Based on these results, the Sponsor has decided A/Indonesia booster will be performed only in subjects having received the 30 µg HA with adjuvant formulation for the primary series and only the 30 µg HA with adjuvant formulation for A/Indonesia booster and primary vaccination series for additional subjects.

In addition the A/Indonesia booster will be performed only in subjects having received the 30 µg HA with adjuvant formulation for the primary series.

As a subset of 140 subjects is sufficient to assess the booster effect of the A/Indonesia 30 µg HA with adjuvant formulation, only subjects included in Center 1 and in Center 3, primed with the 30 µg HA with adjuvant vaccine, will receive the A/Indonesia booster.

As a consequence, all subjects included in Center 4, regardless of the vaccines received at D0 and D21, and subjects having received the 7.5 µg HA without adjuvant vaccine at D0 and D21 will stop the study after M22.

In order to better assess the kinetics of the immune response induced following A/Indonesia booster, an additional visit will be planned 7 days after booster vaccination (V05_Add).

As only the 30 µg HA with adjuvant formulation will be used for the A/Indonesia primary vaccination series, the subset of additional subjects has been changed to 50 instead of 100.

In addition, as for the primary vaccination series the CMI analysis showed a similar Th1/Th2 profile to that of unadjuvanted vaccine and that the theoretical safety issue was not demonstrated, the Sponsor has decided not to evaluate CMI after booster with the A/Indonesia strain.

---

[a] 6-month safety follow-up visit could be replaced by a telephone call.

2 Trial Objectives

2.1 Objectives

- To describe the injection site reactions and systemic safety profile during the 21 days following each of two primary series and one booster (as applicable) intramuscular (IM) injections in two age groups: subjects aged 18 to 60 years (adults) or >60 years (elderly) *(Amendments 1 and 2)*.
- To describe the immune response 21 days after each of two primary series IM injections in two age groups: subjects aged 18 to 60 years (adults) and >60 years (elderly) *(Amendment 2)*.
- To describe the antibody persistence at M6 (D180) (for all subjects), and at M15 and M22 (in a subset of subjects) after the first vaccination in two age groups: subjects aged 18 to 60 years (adults) or >60 years (elderly) *(Amendments 2 and 3)*.
- To describe the immune response 21 days after a booster vaccination administered at 6 months (A/Vietnam booster) or 7 and 21 days after a booster vaccination administered at 22 months (A/Indonesia booster) after the first vaccination in two age groups: subjects aged 18 to 60 years or >60 years *(Amendments 1, 2 and 3)*.
- To describe any serious adverse events (SAEs) during the entire trial.

2.1.1 Endpoints

Safety

- The occurrence, time to onset, number of days of occurrence, and severity of solicited (prelisted in the subject diary and Case Report Form [CRF]) injection site reactions and systemic reactions occurring within 7 days following each injection will be reported.
- The occurrence, nature (Medical Dictionary for Regulatory Activities [MedDRA preferred term), time to onset, duration, severity, relationship to vaccination and seriousness of unsolicited (spontaneously reported) adverse events (AEs) within 21 days following each injection will be reported.
- The occurrence of the following reactions (MedDRA Preferred Terms given in parentheses) in the 3 days following each injection will be more especially reported (as defined by the European Medicines Agency (EMEA) Note for Guidance [CPMP/BWP/214/96]):
  - Injection site induration >5 cm observed for more than 3 days.
  - Injection site ecchymosis (injection site hemorrhage).
  - Rectal equivalent temperature >38°C for 24 hours or more.
  - Malaise.
  - Shivering (chills).
- The occurrence, nature, time to onset, and relationship to vaccination of SAEs during the whole trial (including the 6-month follow-up period) will be reported.

Immunogenicity

Primary series

Hemagglutination inhibition assays will be performed using:

1. Horse erythrocytes for all subjects.
2. Turkey erythrocytes for a subset of 80 subjects in each formulation group (40 subjects per age sub-group) for one vaccine strain only - A/Vietnam (*Amendment 1*).

Anti-Hemagglutinin (anti-HA) Ab titers against the A/H5N1 strain will be expressed as described below:

- Anti-HA Ab titer obtained in duplicate on D0, D21, and D42, and summarized at the subject level by individual geometric means of duplicates at each timepoint. The following endpoints will be derived:
  - Individual titer ratios D21/D0, D42/D0, and D42/D21.
  - Proportion of subjects with anti-HA Ab titer ≥40 (turkey) or ≥32 (horse) 1/dilution (dil) on D0, D21 and D42.
  - Seroconversion (for subjects with a titer <10 [turkey] or <8 [horse] [1/dil] on D0: post-injection titer ≥40 [turkey] or ≥32 [horse] [1/dil])

  or

  - significant increase (for subjects with a titer ≥10 [1/dil] [turkey] or ≥8 [horse]: ≥4-fold increase of the titer) at D21 and D42.

AND

Neutralizing Ab titers against the A/H5N1 strain will be expressed as described below:

- Neutralizing Ab titer obtained in duplicate on D0, D21, and D42 and summarized at the subject level by individual geometric mean of duplicates at each timepoint. The following endpoints will be derived:
  - Individual titer ratios D21/D0, D42/D0, and D42/D21.
  - 2- and 4-fold increase from D0 to D21 and to D42.

Antibody persistence *(Amendment 3)*

Anti-HA Ab titer (HIH method) and neutralizing Ab titer (SN method) at M6 (D180) (for all subjects), and at M15 and M22 (for a subset of subjects).

Booster vaccination response *(Amendment 3)*

An A/Vietnam H5N1 strain will be used for for the 6-month booster, and an A/Indonesia/5/05-RG2 (H5N1) strain (henceforth referred to as A/Indonesia) will be used for the 22-month (see Section 5.1.1). The booster Ab response will be assessed against the homologous strain in the vaccine, with HI test using horse erythrocytes and SN method. In addition, antibody response to heterologous strains used in the booster vaccination may be tested.

- Anti-HA Ab titer obtained in duplicate at M6 (D180) and M6+21D (D201) (if 6-month A/Vietnam booster) or M22 (D670), M22+7D (D677) and M22+21D (D691) (if 22-month

A/Indonesia booster), and summarized at the subject level by individual geometric means of duplicates at each timepoint. The following endpoints will be derived:

- Individual titer ratios M6+21D/M6 (if 6-month A/Vietnam booster) or M22+7D/M22 and M22+21D/M22 (if 22-month A/Indonesia booster).
- Proportion of subjects with anti-HA Ab titer ≥32 (1/dil) at M6 and M6+21D (if 6-month A/Vietnam booster) or M22, M22+7D and M22+21D (if 22-month A/Indonesia booster).
- Seroconversion (for subjects with a titer <8 [1/dil] at M6 or M22: post-injection titer ≥32 [1/dil]) at M6+21D (if 6-month A/Vietnam booster)or M22+7D and M22+21D (if 22-month A/Indonesia booster),

or

- significant increase (for subjects with a titer ≥8 [1/dil] at M6 or D670: ≥4-fold increase of the titer) at M6+21D (if 6-month A/Vietnam booster) or M22+7D and M22+21D (if 22-month A/Indonesia booster).

AND

- Neutralizing Ab titer obtained at M6 and M6+21D (if 6-month A/Vietnam booster) or M22, M22+7D and M22+21D (if 22-month A/Indonesia booster). The following endpoints will be derived:
  - Individual titer ratios M6+21D/M6 (if 6-month A/Vietnam booster) or M22+7D/M22 and M22+21D /M22 (if 22-month A/Indonesia booster).
  - 2- and 4-fold increase from M6 to M6+21D (if 6-month A/Vietnam booster) or from M22 to M22+7D and from M22 to M22+21D (if 22-month A/Indonesia booster).

2.2 Observational Objectives

- To describe the anti-HA antibody response against A/Vietnam and potential other clades using an ELISA test in a subset of 80 subjects in each formulation group (40 subjects per age sub-group) for subjects who receive A/Vietnam in the primary vaccination series only, before the first vaccination and 21 days after the second vaccination (*Amendment 1*).
- To describe in a subset of 80 subjects in each formulation group (40 subjects per age sub-group) for subjects who receive A/Vietnam in the primary vaccination series and only at Center 4 the cellular mediated immune (CMI) response against A/Vietnam and potential other Clade before the first vaccination and 21 days after the second vaccination. *(Amendments 1, 2 and 3).*
- To describe in a subset of 80 subjects in each formulation group (40 subjects per age sub-group) for subjects who receive A/Vietnam in the primary vaccination series only, the anti-neuraminidase antibody response against A/Vietnam and potential other clades before the first vaccination (D0) and 21 days after the second vaccination *(Amendment 1)*

3 Investigators and Trial Organization

The trial will be conducted at three sites in Belgium and at one site in the United Kingdom (UK) under the responsibility of [redacted] MD (Coordinating Investigator, Belgium), [redacted] MD (Belgium), [redacted] MD (Belgium) and [redacted] (Coordinating Investigator, UK).

[redacted]

The two coordinating investigators [redacted] will be signatory Principal Investigators for the Final Clinical Trial Report, and the Sponsor's Responsible Medical Officer (person authorized to sign this protocol and any amendments on behalf of the Sponsor) [redacted] MB BS, Clinical Franchise Leader.

4 Independent Ethics Committee (IEC)/Institutional Review Board (IRB)

Before the investigational product can be shipped to the investigational site and before the inclusion of the first subject in the center, this protocol, the written informed consent forms, consent form updates, subject recruitment procedure (e.g., advertisement) and any other written information to be provided to subjects, must receive favorable opinion from, the appropriate IEC(s)/IRB(s).

Each investigator is responsible for obtaining this approval or favorable opinion before the start of the trial and any subsequent amendments in compliance with Good Clinical Practice (GCP) and local regulations. Copies of these opinions, along with information on the type, version number and date of document, and the date of opinion, must be forwarded by the investigator to the Sponsor together with the composition (names and qualifications of the members attending and voting at the meetings) of the IEC/IRB.

The Coordinating Investigators will submit written summaries of the status of the trial to the IEC/IRB annually, or more frequently if requested. All Serious Adverse Reactions occurring during the trial will be reported to the IEC/IRB, according to IRB policy.

5 Investigational Plan

5.1 Description of the Overall Trial Design and Plan

5.1.1 Trial Design (*Amendments 1, 2 and 3*)

The trial is a Phase II open randomized multi-center trial. A total of 650 subjects will be included in the trial.

Of these, 600 subjects (300 subjects aged 18 to 60 years and 300 subjects aged >60 years) will receive two vaccinations (at D0 and D21) of A/Vietnam as a primary series of either 30 µg HA with adjuvant or 7.5 µg HA without adjuvant.

A subset of these 600 subjects (N=160, i.e. subjects included in Center 2) receiving A/Vietnam as the primary series will receive a booster vaccination of 7.5 µg HA without adjuvant at M6.

A subset of 140 subjects, i.e. subjects included in Center 1 and 3, receiving the 30 µg HA with adjuvant formulation as primary series will receive a booster vaccination at 22 months (with 30 µg HA with adjuvant of an A/IndonesiaA/H5N1 vaccine strain).

The remaining of subjects, i.e. subjects receiving the 7.5 µg HA without adjuvant as primary series in centers 1 and 3 and all subjects in center 4, regardless of the vaccine received in primary series, will receive no booster and stop the trial at M22

Figure 5 is for subjects receiving the 7.5µg HA without adjuvant as primary series *(Amendment 3)*.

Figure 5: Trial Design Showing Booster Vaccinations Following a Primary Series of 7.5 µg HA of A/Vietnam/1194/2004/NIBRG-14 (H5N1)

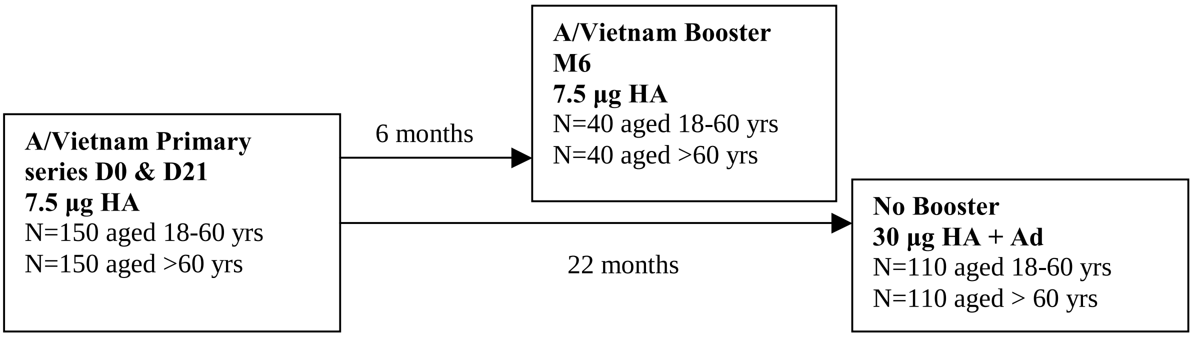

Figure 6 is for subjects receiving the 30 µg HA with adjuvant as primary series *(Amendment 3)*

Figure 6: Trial Design Showing Booster Vaccinations Following a Primary Series of 30 µg or A/Vietnam H5N1 in Centers 1, 2 and 3

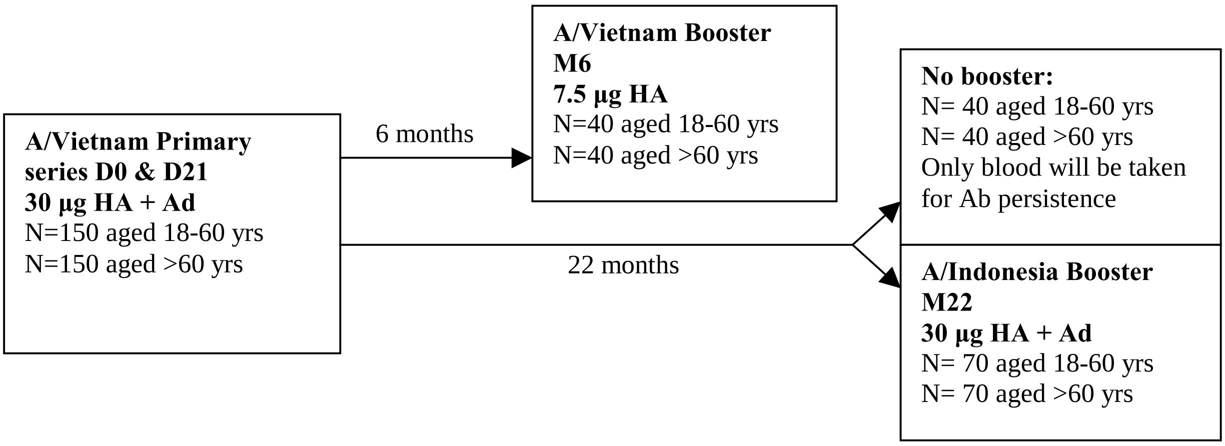

In addition, 50 subjects (aged 18 to 60 years) will be included in Center 2 at the time of the A/Indonesia booster to receive two vaccinations (at D0 and D21) of A/Indonesia as a primary series of 30 HA µg with adjuvant. These vaccinations will be performed in parallel with the A/Indonesia booster. The subjects receiving A/Indonesia as a primary series will receive no booster vaccination.

There will therefore be a total of 11 subsets of subjects, depending on primary vaccination (dose and strain), booster vaccination (schedule, dose and strain), age, and center, as summarized below:

Table 1: Summary of Primary Vaccination Series Type and Dose and Booster Vaccination Type, Dose and Timing

Subset of subjects	N	Age (years)	Primary Series (D0 and D21)	Booster	
				6 Months	22 Months
1	40	18 to 60	30 µg + Ad; A/Vietnam	7.5 µg; A/Vietnam	-
2	70	18 to 60	30 µg + Ad; A/Vietnam	-	30 µg + Ad; A/Indonesia
3	40	18 to 60	30 µg + Ad; A/Vietnam	-	-
4	40	>60	30 µg + Ad; A/Vietnam	7.5 µg; A/Vietnam	-
5	70	>60	30 µg + Ad; A/Vietnam	-	30 µg + Ad; A/Indonesia
6	40	>60	30 µg + Ad; A/Vietnam	-	-
7	40	18 to 60	7.5 µg; A/Vietnam	7.5 µg; A/Vietnam	-
8	110	18 to 60	7.5 µg; A/Vietnam	-	-
9	40	>60	7.5 µg; A/Vietnam	7.5 µg; A/Vietnam	-
10	110	>60	7.5 µg; A/Vietnam	-	-
11	50	18 to 60	30 µg + Ad; A/Indonesia	-	-

N = number of subjects
Ad = aluminum hydroxide adjuvant (600 µg Al^{3+})
A/Vietnam: A/Vietnam/1194/2004/NIBRG14 (H5N1)
A/Indonesia: A/Indonesia/5/05-RG2 (H5N1)

5.1.2 Trial Plan

The trial plan is summarized in the Trial Flow-Chart (Figure 1, Figure 2, Figure 3 and Figure 4 in the protocol synopsis) and in Sections 5.1.1 and 5.1.3.

Recruitment and information of subjects:

Before the inclusion, the investigator may orally inform potentially eligible subjects about the trial. They may be given an oral description of the trial design, presenting the general benefits and discomforts related to the trial. They may be informed that they may return and receive further information and sign the full informed consent during the recruitment period. The process of subject recruitment and any oral or written information that will be provided to the subjects must be documented. This will be available in the investigator's file and the Trial Master File. It is

highly recommended to document in the subject's medical file (source documents) the transmission of oral information to the subject at the time of a medical visit to the investigator.

Trial description: (*Amendments 1, 2 and 3****)***

After having signed the informed consent form, eligible subjects will be included in the study and will be randomized to receive primary vaccinations of either 7.5 µg HA without adjuvant or 30 µg HA with adjuvant. Subjects will also be required to sign the informed consent form addendum (*added in Amendment 1*). Subjects who received primary vaccinations of the A/Vietnam strain will receive:

- a booster vaccination at 6 months for a subset of 160 subjects (80 adults and 80 elderly subjects in each formulation group included in Center 2

or

- a booster vaccination at 22 months after the second vaccination for a subset of 140 subjects (70 adults and 70 elderly subjects) primed with the 30 µg HA with adjuvant formulation and included in Centers 1 and 3

or

- no booster for the remainder subjects (N=300, 150 adults and 150 elderly subjects), i.e subjects receiving the 7.5 µg HA without adjuvant as primary series in centers 1 and 3 and all subjects in center 4, whatever the vaccine received for primary series

Subjects included in Center 2 to receive primary vaccination series of the A/Indonesia strain will receive no booster vaccination (as described in Section 5.1.1). Each subject will be followed up for 6 months after the last vaccination and will provide three, five, six or eight blood samples (see trial flow-charts).

Immunogenicity will be evaluated 21 days after the first and second vaccinations (for all subjects), at M6, M15, M22 (if applicable) and before the booster vaccination (if applicable), and 21 days after the booster vaccination (if applicable). In addition, immunogenicity will be evaluated 7 days after the A/Indonesia booster, as applicable. Subjects will be observed for safety by the investigator for 30 minutes after each vaccination, and any AE/reaction occurring during the 21 days after each vaccination will be recorded using a Diary Card (DC). In addition, all SAEs will be recorded during the entire trial (including the 6 month follow-up period).

Center-specific populations:

- CMI will be described in 160 subjects that are to be recruited at Center 4 [redacted].
- HI using turkey erythrocytes, neuraminidase, and ELISA will be described in a subset of 80 subjects in each formulation group who received A/Vietnam as the primary series recruited across the four centers.
- Subjects receiving the booster vaccination at 6 months will be those included at Center 2, [redacted]

- Subjects receiving the A/Indonesia booster will be those included in Center 1[redacted] and Center 3[redacted]
- Subjects receiving the A/Indonesia strain as the primary vaccination series (and no booster vaccination) will be recruited in parallel with the administration of the A/Indonesia booster in Center 2.

5.1.3 Visit Procedures

V01: First vaccination (D0) and blood sampling:

- The investigator or delegate will:

1) obtain informed consent by presenting the trial to the subject, answer any of his/her questions, and ensure that the subject has been informed of all aspects of the trial that are relevant to his/her decision to participate.
2) date and sign the informed consent form, after it has been dated and signed by the subject (the investigator will retain the original of this document and will give the copy to the subject).
3) perform a urine pregnancy test for women of child-bearing age and potential.
4) check all inclusion and exclusion criteria through physical examination and medical interview.
5) allocate a trial inclusion number to the subject (number of center and order of inclusion).
6) record the subject's initials, subject number, date of birth, height, weight, sex, ethic origin (Caucasian, Asian, Black, or other) (specifically for additional subjects) (*Amendment 1*), past and current significant medical history, and all information related to points 3 to 5 (above) in the source document and then in the CRF.
7) record details of any influenza-like illness (ILI) and inter-pandemic influenza vaccination (including dates of occurrence) in the CRF.(specifically for additional subjects) (*Amendment 1*)
8) take a 30 mL blood sample in a dry tube for serology evaluation in all subjects, and a further 20 mL sample for CMI response evaluation from subjects included at Center 4 only. He/she will update the source document and appropriate designated forms.

1) If blood sampling for serology cannot be performed, the subject will not be vaccinated. In this case, his/her subject number will not be reassigned.

9) scratch the randomization list to define the vaccine dose corresponding to the subject's number, if applicable.
10) administer the vaccine dose corresponding to the treatment group.
11) affix the corresponding detachable label in the source document and CRF.
12) ensure the 30-minute observation period following vaccination is observed.
13) complete the source document and CRF.

14) provide the subject with the Diary Card 1 (DC1) to be filled in until V02, a digital thermometer and a ruler, and instruct the subject how to use these tools and how to complete the first diary card.
15) make an appointment 21 days ± 3 days after vaccination.

V02: Second vaccination (D21) and blood sampling:

- The investigator or delegate will:

1) interview the subject, requesting information concerning any SAE that may have occurred since V01. The occurrence of any SAE will be reported in the CRF and the Initial and Follow-up reporting forms will be completed and sent to the Sponsor immediately.
2) collect and review DC1 filled in by the subject since D0 and record any additional information and/or discrepancies in the source documents.
3) examine and interview the subject and record new findings in the source documents.
4) record any injection site reactions, systemic reactions/events, and concomitant medications taken (see Section 6.8) in the CRF.
5) take a 30 mL blood sample in a dry tube for serology evaluation. He/she will update the source document and appropriate designated forms.
6) perform a urine pregnancy test for women of child-bearing age and potential.
7) check all temporary and definite contraindications through physical examination and medical interview (in case of temporary contraindication, the vaccination will be delayed; in case of definite contraindication, the vaccination will not be performed).
8) administer the vaccine dose corresponding to the subject's number (the vaccine dose will be prepared by the pharmacist/nurse/technician).
9) affix the corresponding detachable label in the source document and CRF.
10) ensure the 30-minute observation period following vaccination is observed.
11) complete the source document and CRF.
12) provide the subject with DC2 to be filled in until V03.
13) remind the subject to contact the investigator in case of any SAE between V02 and V03.
14) make an appointment 21 days ± 3 days after second vaccination.

V03: Safety follow-up visit and blood sampling (D42):

- The investigator or delegate will:

1) interview the subject, requesting information concerning any SAE that may have occurred since V02. The occurrence of any SAE will be reported in the CRF and the Initial and Follow-up reporting forms will be completed and sent to the Sponsor immediately.
2) collect and review DC2 filled in by the subject since D21 and record any additional information and/or discrepancies in source documents.

3) perform physical examination and medical interview based on the information contained in the DC2 and complete the source document.
4) record any injection site reactions, systemic reactions/events, and concomitant therapies in the CRF.
5) take a 30 mL blood sample in a dry tube for serology evaluation in all subjects, and a further 20 mL sample for CMI response evaluation from subjects included at Center 4 only. He/she will update the source document and appropriate designated forms.
6) complete the source document and CRF.
7) provide the subject with Memory Aid 1 (MA1) to be used as necessary until V04.
8) complete and sign the primary series termination record in the CRF.
9) make an appointment for the next visit (D180 days ± 8 days) or for the next contact (D180 + 15 days) (for the additional subjects).

----IF BOOSTER AT 6 MONTHS (Center 2)----

V04: Booster vaccination and blood sampling (D180, M6):

- The investigator or delegate will:

1) present the trial to the subject again, answer any of his/her questions and ensure that the subject has been informed of all aspects of the trial that are relevant to his/her decision to continue his/her participation *(added in Amendment 1)*.
2) date and sign the informed consent form addendum 1, once the subject has signed and dated it (the investigator will retain the original of this document and give the copy to the subject) *(added in Amendment 1)*.
3) interview the subject, requesting information concerning any SAE that may have occurred since V03. The occurrence of any SAE will be reported in the CRF and the Initial and Follow-up reporting forms will be completed and sent to the Sponsor immediately.
4) collect and review MA1, used by the subject since D42 and record any additional information and/or discrepancies in source documents.
5) perform physical examination and complete the source document.
6) record the ethnic origin (Caucasian, Asian, Black, or other) in the source document and then in the CRF (*Amendment 1*).
7) record details of any influenza-like illness (ILI) and inter-pandemic influenza vaccination (including dates of occurrence) that occurred in the year prior to V01 and between V01 and V04 in the CRF (*Amendment 1*).
8) take a 30 mL blood sample in a dry tube for serology evaluation in all subjects. He/she will update the source document and appropriate designated forms.
9) perform a urine pregnancy test for women of child-bearing age and potential.

10) check all temporary and definite contraindications through physical examination and medical interview (in case of temporary contraindication, the vaccination will be delayed; in case of definite contraindication, the vaccination will not be performed).
11) administer the vaccine dose corresponding to the subject's number (the vaccine dose will be prepared by the pharmacist /nurse/technician).
12) affix the corresponding detachable label in the source document and CRF.
13) ensure the 30-minute observation period following vaccination is observed.
14) complete the source document and CRF.
15) provide the subject with DC3 to be filled in until V05.
16) remind the subject to contact the investigator in case of any SAE between V04 and V05.
17) make an appointment 21 days ± 3 days after the booster vaccination.

V05: Blood sampling (M6 + 21 days):

- The investigator or delegate will:

1) interview the subject, requesting information concerning any SAE that may have occurred since V04. The occurrence of any SAE will be reported in the CRF and the Initial and Follow-up reporting forms will be completed and sent to the Sponsor immediately.
2) collect and review DC3 filled in by the subject since V04 and record any additional information and/or discrepancies in source documents.
3) perform physical examination and medical interview based on the information contained in the DC3 and complete the source document.
4) record any injection site reactions, systemic reactions/events, and concomitant therapies in the CRF.
5) take a 30 mL blood sample in a dry tube for serology evaluation in all subjects. He/she will update the source document and appropriate designated forms.
6) complete the source document and CRF.
7) provide the subject with MA2 to be used as necessary until the 6-month follow-up.
8) make an appointment for the 6 month follow-up contact (VAC3 + 180D+15 days).
9) complete and sign the booster series second termination record in the CRF.

----IF NO BOOSTER (Amendment 3) (Centers 1 and 3 for subjects receiving the 7.5 µg HA formulation at D0 and D21 and Center 4 for all subjects)---

V04: Blood sampling (D180, M6):

- The investigator or delegate will:

1) present the trial to the subject again, answer any of his/her questions and ensure that the subject has been informed of all aspects of the trial that are relevant to his/her decision to continue his/her participation *(added in Amendment 1)*

2) date and sign the addendum #1 to the informed consent form, once the subject has signed and dated it (the investigator will retain the original of this document and give the copy to the subject) *(added in Amendment 1)*
3) interview the subject, requesting information concerning any SAE that may have occurred since V03. The occurrence of any SAE will be reported in the CRF and the Initial and Follow-up reporting forms will be completed and sent to the Sponsor immediately.
4) collect and review MA1, used by the subject since D42 and record any additional information and/or discrepancies in source documents.
5) perform physical examination and complete the source document.
6) record the ethnic origin (Caucasian, Asian, Black, or other) in the source document and then in the CRF (*Amendment 1*).
7) record details of any influenza-like illness (ILI) and inter-pandemic influenza vaccination (including dates of occurrence) that occurred in the year prior to V01 and between V01 and V04 in the CRF (*Amendment 1*).
8) take a 30 mL blood sample in a dry tube for serology evaluation. He/she will update the source document and appropriate designated forms.
9) complete the source document and CRF.
10) provide the subject with MA2 to be used as necessary until V04_Add.
11) make an appointment for the next visit (M15 + 15 days).
12) complete and sign the second termination record in the CRF.

V04_Add: Blood sampling (D450, M15):

- The investigator or delegate will:

1) present the trial to the subject again, answer any of his/her questions and ensure that the subject has been informed of all aspects of the trial that are relevant to his/her decision to continue his/her participation
2) date and sign the addendum 2 to the informed consent form, once the subject has signed and dated it (the investigator will retain the original of this document and give the copy to the subject)
3) check all inclusion criteria
4) interview the subject, requesting information concerning any SAE that may have occurred since V04. The occurrence of any SAE will be reported in the CRF and the Initial and Follow-up reporting forms will be completed and sent to the Sponsor immediately.
5) collect and review MA2, used by the subject since V04 and record any additional information and/or discrepancies in source documents.
6) perform physical examination and complete the source document.
7) take a 30 mL blood sample in a dry tube for serology evaluation. He/she will update the source document and appropriate designated forms.

8) provide the subject with MA3 to be used as necessary until V05.
9) make an appointment for the next visit (M22 + 15 days)

V05: Blood sampling for subjects receiving the 30 µg HA with adjuvant for primary series (D670, M22):

- The investigator or delegate will:

1) interview the subject, requesting information concerning any SAE that may have occurred since V04_Add. The occurrence of any SAE will be reported in the CRF and the Initial and Follow-up reporting forms will be completed and sent to the Sponsor immediately.
2) collect and review MA3, used by the subject since V04_Add and record any additional information and/or discrepancies in source documents.
3) perform physical examination and complete the source document.
4) take a 30 mL blood sample in a dry tube for serology evaluation in all subjects. He/she will update the source document and appropriate designated forms.
5) complete the source document and CRF.
6) complete and sign the termination record in the CRF and document the end of the participation in the study in the source documents.

V05: Safety follow-up for subjects receiving the 7.5 µg HA without adjuvant for primary series (D670, M22):

- The investigator or delegate will:

1) interview the subject, requesting information concerning any SAE that may have occurred since V04_Add. The occurrence of any SAE will be reported in the CRF and the Initial and Follow-up reporting forms will be completed and sent to the Sponsor immediately.
2) collect and review MA3, used by the subject since V04_Add and record any additional information and/or discrepancies in source documents.
3) complete the source document and CRF.
4) complete and sign the termination record in the CRF and document the end of the participation in the study in the source documents.

----IF A/INDONESIA (Amendment 2) BOOSTER (Centers 1 and 3 for subjects receiving the 30 µg HA with adjuvant formulation at D0 and D21)---

V04: Blood sampling (D180, M6):

- The investigator or delegate will:

1) present the trial to the subject again, answer any of his/her questions and ensure that the subject has been informed of all aspects of the trial that are relevant to his/her decision to continue his/her participation *(added in Amendment 1)*

2) date and sign the addendum #1 to the informed consent form, once the subject has signed and dated it (the investigator will retain the original of this document and give the copy to the subject) *(added in Amendment 1)*
3) interview the subject, requesting information concerning any SAE that may have occurred since V03. The occurrence of any SAE will be reported in the CRF and the Initial and Follow-up reporting forms will be completed and sent to the Sponsor immediately.
4) collect and review MA1, used by the subject since D42 and record any additional information and/or discrepancies in source documents.
5) perform physical examination and complete the source document.
6) record the ethnic origin (Caucasian, Asian, Black, or other) in the source document and then in the CRF (*Amendment 1*).
7) record details of any influenza-like illness (ILI) and inter-pandemic influenza vaccination (including dates of occurrence) that occurred in the year prior to V01 and between V01 and V04 in the CRF (*Amendment 1*).
8) take a 30 mL blood sample in a dry tube for serology evaluation. He/she will update the source document and appropriate designated forms.
9) complete the source document and CRF.
10) provide the subject with MA2 to be used as necessary until V04_Add.
11) make an appointment for the next visit (M15 + 15 days).
12) complete and sign the second termination record in the CRF.

V04_Add: Blood sampling (D450, M15) (Amendment 2):

- The investigator or delegate will:

1) present the trial to the subject again, answer any of his/her questions and ensure that the subject has been informed of all aspects of the trial that are relevant to his/her decision to continue his/her participation
2) date and sign the addendum 2 to the informed consent form, once the subject has signed and dated it (the investigator will retain the original of this document and give the copy to the subject)
3) check all inclusion criteria
4) interview the subject, requesting information concerning any SAE that may have occurred since V04. The occurrence of any SAE will be reported in the CRF and the Initial and Follow-up reporting forms will be completed and sent to the Sponsor immediately.
5) collect and review MA2, used by the subject since V04 and record any additional information and/or discrepancies in source documents.
6) perform physical examination and complete the source document.
7) take a 30 mL blood sample in a dry tube for serology evaluation. He/she will update the source document and appropriate designated forms.

8) provide the subject with MA3 to be used as necessary until V05.
9) complete and sign the third termination record in the CRF.
10) make an appointment for the next visit (M22 + 15 days)..

V05: Booster vaccination and blood sampling (D670, M22) (Amendment 3):

- The investigator or delegate will:

1) present the trial to the subject again, answer any of his/her questions and ensure that the subject has been informed of all aspects of the trial that are relevant to his/her decision to continue his/her participation *(added in Amendment 3)*
2) date and sign the addendum 3 to the informed consent form, once the subject has signed and dated it (the investigator will retain the original of this document and give the copy to the subject) *(added in Amendment 3)*
3) interview the subject, requesting information concerning any SAE that may have occurred since V04_Add. The occurrence of any SAE will be reported in the CRF and the Initial and Follow-up reporting forms will be completed and sent to the Sponsor immediately.
4) collect and review MA3, used by the subject since V04_Add and record any additional information and/or discrepancies in source documents.
5) perform physical examination and complete the source document.
6) take a 30 mL blood sample in a dry tube for serology evaluation in all subjects. He/she will update the source document and appropriate designated forms.
7) perform a urine pregnancy test for women of child-bearing age and potential.
8) perform a urine pregnancy test for women of child-bearing age and potential
9) check all temporary and definite contraindications through physical examination and medical interview (in case of temporary contraindication, the vaccination will be delayed; in case of definite contraindication, the vaccination will not be performed).
10) administer the vaccine dose
11) affix the corresponding detachable label in the source document and CRF.
12) ensure the 30-minute observation period following vaccination is observed.
13) complete the source document and CRF.
14) provide the subject with DC3 to be filled in until V06.
15) remind the subject to contact the investigator in case of any SAE between V05 and V05_Add.
16) make an appointment 7 days ± 1 days after the booster vaccination.

V05_Add: Blood sampling (V05 + 7 days) (Amendment 3):

- The investigator or delegate will:

1) interview the subject, requesting information concerning any SAE that may have occurred since V05. The occurrence of any SAE will be reported in the CRF and the Initial and Follow-up reporting forms will be completed and sent to the Sponsor immediately.
2) take a 30 mL blood sample in a dry tube for serology evaluation in all subjects. He/she will update the source document and appropriate designated forms.
3) check the DC3 filled in by the subject since the third vaccination and record any additional information and/or discrepancies in source documents.
4) perform a physical examination and medical interview based on the information contained in the DC and complete the source documents
5) give back to the subject the DC3 to be continued to be filled in until V06
6) remind the subject to contact the investigator in case of any SAE between V05_Add and V06.
7) make an appointment for V06 (14 ± 3 days after V05_Add).

V06: Blood sampling (V05 + 21 days):

- The investigator or delegate will:

1) interview the subject, requesting information concerning any SAE that may have occurred since V05_Add. The occurrence of any SAE will be reported in the CRF and the Initial and Follow-up reporting forms will be completed and sent to the Sponsor immediately.
2) collect and review DC3 filled in by the subject since V05 and record any additional information and/or discrepancies in source documents.
3) perform physical examination and medical interview based on the information contained in the DC and complete the source document.
4) record any injection site reactions, systemic reactions/events, and concomitant therapies in the CRF.
5) take a 30 mL blood sample in a dry tube for serology evaluation in all subjects. He/she will update the source document and appropriate designated forms.
6) complete the source document and CRF.
7) provide the subject with MA4 to be used as necessary until the 6-month follow-up.
8) complete and sign the booster series termination record in the CRF.
9) make an appointment for the 6 month follow-up contact (VAC3 + 180D+15 days).

----For all subjects--

6-month follow-up (Amendment 1)

- The investigator or delegate will:

1) make at least three attempts to call the subject by telephone (or arrange for a visit at the investigational site if it is not possible to contact the subject by telephone), documented in the source documents; at least 6 months (+15 days) after the last injection, to collect information on SAEs.

2) in the event of any SAE, arrange a visit with the subject to obtain further information.
3) collect and/or review appropriate Memory Aid, used by the subject since the previous visit and record any additional information and/or discrepancies in source documents.
4) perform physical examination (or medical interview if visit is replaced by telephone call) and complete the source document.

5.1.4 Planned Trial Calendar

The calendar presented in the following table is indicative. The real dates may differ as, for example, the trial will not start until all the appropriate regulatory and ethical approvals have been obtained.

Table 2: Planned Trial Calendar (*Amendments 1, 2 and 3*)

**Planned trial period (FVFS* to LVLS*)**	16 May 06 to October 08
**Planned Inclusion period (FVFS* to FVLS*)**	
**A/Vietnam primary series**	16 May 06 to 12 June 2006
**A/Indonesia primary series**	03 Mar 08 to 05 May 08
**Planned end of trial†**	October 08
**Planned date of final clinical trial report**	February 09

* FVFS: First visit of first subject; LVLS: Last visit of last subject; FVLS: First visit of last subject (last first visit)

† End of trial is defined as the date of the last contact with a trial subject within the scope of the trial. In case of multicenter trials in multiple countries, it is the date of the last contact with a trial subject in the last country

5.2 Discussion of Trial Design

In preparation for an influenza pandemic, the EMEA has developed licensing guidelines for pandemic influenza vaccines (13) (14).

Inclusion of subjects in a first, formulation/dose-finding study (GPA01) (12) was completed in June 2005 and the results of the first two vaccinations (D0 to D42) of this trial have been used to identify the formulations, doses, and administration schedule used in the present trial.

Based on these data, two dose levels have been chosen for the present trial (30 µg with adjuvant and 7.5 µg without adjuvant) and a two-dose schedule (separated by 21 days) with a 6 month or 22-month booster. In terms of immunogenicity the optimal formulation/dose used in the previous—GPA01 (12)—study was 30 µg with adjuvant. Additionally, the 7.5 µg dose without adjuvant was also immunogenic and is of interest from a dose sparing perspective.

The chosen adjuvant is aluminum hydroxide, which is commonly used and can induce a strong and sustained humoral response (15).

The H5N1 viruses chosen for development of pre-pandemic candidate vaccine viruses are representative of antigenically and genetically distinct groups of viruses that have infected humans primarily through contact with ill or dead H5N1-infected birds.

The HA sequences of the majority of H5N1 viruses circulating in avian species during the past 3 years can be separated into two phylogenic clades. A/Vietnam viruses circulate in Cambodia, Thailand and Vietnam and were responsible for human infections during 2004 and 2005 in these countries. A/Indonesia viruses circulated in birds in China and Indonesia during 2003-2004 and spread to the Middle East, Europe and Africa during 2005-2006.

This latter genetic group of viruses (A/Indonesia/05/2005/RG2) has been principally responsible for human infections during late 2005 and in 2006.

As it is not known if the next influenza pandemic will be caused by H5N1 viruses or which of the clades or subclades of H5N1 would be responsible, clinical trials using A/Vietnam viruses continue as an essential element in pandemic preparedness to maximize the available data on priming, cross-reactivity and cross-protection by vaccine viruses from different clades.

In this context, the Sponsor has decided to administer a new strain (A/Indonesia) as the booster vaccination that is slightly different from that used in the vaccine which was received in primary series (*Amendments 1 and 2*).

Following booster results of GPA01 and GPA02, which showed that the 30 µg HA adjuvanted booster induced a higher immune response than the 7.5 µg HA without adjuvant booster, and that the response was more important in subjects having received an adjuvanted vaccine for primary series than in the other subjects, the Sponsor has decided not to assess the effect of A/Indonesia booster of 7.5 µg HA without adjuvant formulation. Indeed, the 7.5 µg HA formulation administered as a booster did not a strong immune response compared to the 30 HA with adjuvant formulation (Amendment3).

5.3 Selection of Trial Population

5.3.1 Recruitment Procedures

5.3.1.1 Belgian Sites

The Belgian sites have their own volunteer databases. Potential subjects already entered in the database will be contacted by telephone or by mail. If necessary, advertisements will be posted in different locations (e.g. University) to recruit the targeted number of volunteers. In this case, volunteers will contact the site directly.

5.3.1.2 UK Site

At the [redacted] site, recruitment will be conducted through information sheets mailed out to all residents within specified postal area. Individuals interested in participating in the study will contact the [redacted] and discuss the study with research staff who will screen the potential participant. If the individual is interested in participating and meet all the inclusion criteria and none of the exclusion criteria then an appointment will be made to see the potential participant in the clinical rooms of the [redacted] during the recruitment period. At this appointment, formal informed consent will be obtained.

Posters outlining the study and advertisements may also be used in order to maximize recruitment.

5.3.2 Participant Information and Consent

Informed consent is the process by which a subject voluntarily confirms his or her willingness to participate in a particular trial, after being informed of all relevant aspects of the trial. Informed consent is documented by means of a written, signed and dated informed consent form.

The subject must give written informed consent before being included in the trial. Prior to signing the consent form, the subject must be informed by the appropriate study personnel about the nature and purpose of the trial in accordance with Good Clinical Practice and must have sufficient time and opportunity to ask any questions.

In addition, if new information becomes available that may be relevant to the subject's willingness to continue participation in the trial, this will be communicated in a timely manner to the subject. The communication of this information will be provided and documented via a revised consent form or addendum to the original consent form.

Such addendum will be provided at V04, V04_Add and V05 to inform the subjects of the modifications to the protocol. These addenda to the informed consent form should be signed by subjects at V04 (Addendum 1 – for subjects who were to receive a booster at M6 or later) and V04_Add and V05 (Addendum 2 and 3 – for subjects who were to receive the A/Indonesia booster). (*Added in Amendments 1, 2 and 3*).

In addition, an explanation letter will be provided to all subjects to explain the changes of the design.

A sample informed consent form and the addenda to the informed consent form are provided in Appendix 2.

Informed consent forms and the addenda will be provided in duplicate (the original will be kept by the investigator and the copy kept by the subject or the subject's legally acceptable representative).

5.3.3 Inclusion Criteria

The following inclusion criteria will be checked at V01 before inclusion:

1) Aged over 18 years on the day of inclusion, and 18 to 60 years for the 50 additional subjects for A/Indonesia primary series *(Amendment 3)*.
2) Informed consent form signed.
3) Able to attend all scheduled visits and to comply with all trial procedures.
4) For a woman, inability to bear a child or negative urine pregnancy test.
5) For a woman of child-bearing potential, use of an effective method of contraception or abstinence for at least 4 weeks prior and at least 4 weeks after to each vaccination.

The following inclusion criterion will be checked at V04 for subjects who are to receive a booster at M6 or M22 (*Amendments 1 and 3*).

6) Addendum 1 of the informed consent form signed and dated by the subject

The following inclusion criterion will be checked at V04 Add for subjects who are to receive a A/Indonesia booster (*Amendment 2*).

7) Addendum 2 of the informed consent form signed and dated by the subject

The following inclusion criterion will be checked at V05 for subjects who are to receive a A/Indonesia booster (*Amendment 3*).

8) Addendum 3 of the informed consent form signed and dated by the subject

5.3.4 Exclusion Criteria

The following exclusion criteria will be checked at V01 before inclusion:

1) Systemic hypersensitivity to any component of the vaccine or a life-threatening reaction after previous administration of a vaccine containing the same substances (egg proteins, chick proteins, thimerosal, aluminum, neomycin, formaldehyde, and octoxinol 9[a]).
2) Febrile illness (oral temperature ≥37.5°C) on the day of inclusion.
3) Breast-feeding.
4) Previous vaccination with an avian flu vaccine.

[a] The list of vaccine components is included in the Investigator’s Brochure.

5) Participation in a clinical trial (drug, device, or medical procedure) within 4 weeks prior to the first vaccination[a].
6) Planned participation in another clinical trial during the present trial period.
7) Congenital or acquired immunodeficiency, or receipt of immunosuppressive therapy such as anti-cancer chemotherapy or radiation therapy within the preceding 6 months or long-term systemic corticosteroid therapy[b].
8) Chronic illness that could interfere with trial conduct or completion (e.g. cardiac, renal, diabetes, or auto-immune disorders[c]).
9) Current alcohol or drug abuse that may interfere with the subject's ability to comply with trial procedures.
10) Receipt of blood or blood-derived products within the past 3 months.
11) Any vaccination within 4 weeks prior to the first trial vaccination.
12) Vaccination planned within 4 weeks after any trial vaccination.
13) Thrombocytopenia or bleeding disorder contraindicating IM vaccination.
14) Subject deprived of freedom by an administrative or court order, or in an emergency setting, or hospitalized without his/her consent.

5.3.5 Temporary Contraindications

Should the following condition occur, the investigator will postpone the second or booster vaccination until the condition is resolved.

- Acute febrile illness within the 72 hours preceding the vaccination or oral temperature ≥37.5°C on the day of the vaccination.

Vaccination should be postponed within the timeframe for vaccination indicated in the trial flow-chart (see Figure 1, Figure 2,

[a] Participation in another clinical trial investigating a vaccine, a drug, a medical device, or a medical procedure.

[b] Systemic corticosteroid therapy (prednisolone or equivalent) for more than two consecutive weeks within the past 3 months.

[c] Chronic illness may include, but is not limited to, cardiac, renal or auto-immune disorders or diabetes.

Figure 3 and Figure 4).

5.3.6 Removal of Participants from Treatment or Assessment

Withdrawn subjects will not be replaced.

5.3.6.1 Conditions for Withdrawal

A subject may decide to stop participating in the trial for any reason and at any time. In that case, the investigator must determine that the reason for withdrawal is not related to an AE. The reason for a subject withdrawing during the trial will be specified in the source document and in the relevant section of the CRF.

Subjects will be informed that they have the right to withdraw from the trial at any time.

The investigator will discontinue the second or booster vaccination in the case of an event considered to be a definite contraindication (see following list). The vaccination will be delayed in the event of a temporary contraindication (see Section 5.3.5).

Definite Contraindications

The following contraindications will be verified before the second and booster vaccinations:

1) Subject allergic to one of the constituents of the vaccine (particularly egg and chicken protein).
2) Blood or blood derived products received in the past 3 months or ongoing at the visit.
3) Immunosuppressive therapy such as anti-cancer chemotherapy or radiation therapy in the past 6 months or ongoing at the visit, or long-term systemic corticosteroid therapy (for more than two consecutive weeks in the past 3 months before the visit).
4) Pregnancy (positive urine test).
5) Vaccination other than the trial vaccine 4 weeks prior to and 4 weeks after any administration of the trial vaccine.
6) A SAE related to the trial vaccine following a vaccination.

5.3.6.2 Lost to Follow-up Procedures

In the case of subjects who fail to attend a follow-up visit, documented reasonable effort (e.g., documented telephone calls and certified mail) should be undertaken to locate or recall him/her or at least to determine their health status while fully respecting the subject's rights. These efforts (e.g., letter, several phone calls, home-visit) should be documented in the subject’s source document and CRF.

5.3.6.3 Termination Classification

The investigator will classify the termination status of each subject at the end of the trial in the termination page of the CRF according to the following:

- SAE.
- Other AE.
- Non compliance with the protocol.
- Lost to follow up.
- Voluntary withdrawal not due to an AE.

5.3.6.4 Follow-up of Discontinuations

Any subject who has been discontinued from the trial because of an AE considered to be related to the vaccine will be followed as deemed appropriate by the investigator until resolution of the event or until chronicity of the event has been established. This will be documented in the source document and in the CRF.

Any subject who has been withdrawn from the trial because of an AE considered to be not related to the vaccine will be followed as deemed appropriate by the investigator.

5.3.6.5 Follow-up and Reporting of Pregnancies

Pregnancy is an exclusion criterion for enrolment in this trial, but subjects could potentially become pregnant during their period of participation. Although pregnancy is a normal state, pregnancy occurring during the trial is to be recorded as an AE (but not as a SAE). Any complications during pregnancy (e.g., gestational diabetes or eclampsia) are also to be considered as AEs, however, these complications could result in the event being a SAE.

The investigator is required to complete Part 1 of the "Initial Pregnancy Report Form" as soon as he/she is aware that the subject is pregnant. Part 1 contains information that includes the date of the last menstrual period, expected date of delivery, and the dates of vaccination. Part 2 contains follow-up information about delivery and newborn or pregnancy termination and is required to be completed at a follow-up after the due date.

5.3.7 Medical History

Significant medical history (past and ongoing conditions) will be documented in the CRF. For each condition, the following data will be collected:

- Diagnosis.
- Presence of the condition at enrollment.

The reporting of diagnosis (rather than signs or symptoms) will be encouraged.

Routine health care visits for pre-existing conditions, routine check-ups, medication prescription renewals, and stable pre-existing conditions as well as planned hospitalizations for elective

surgery will not be recorded as AEs. Worsening of any documented pre-existing condition will become a reportable AE.

5.4 Modification of the Trial and Protocol

No amendments to this trial plan and protocol will be made without consultation with, and agreement of, the Sponsor. Any amendment to the trial that appears necessary during the course of the trial must be discussed by the investigator and Sponsor concurrently. If agreement is reached concerning the need for an amendment, it will be produced in writing by the Sponsor and/or the investigator and will become a formal part of the protocol. An amendment requires IEC/IRB approval. All amendments must also be forwarded to any applicable Regulatory Authorities.

An administrative change to the protocol is one that modifies administrative and logistical aspects of a protocol but does not affect the subjects' safety, the objectives or the progress of the trial. An administrative change does not require IEC/IRB approval. However, the IEC/IRB and the relevant Health Authorities must be notified whenever an administrative change is made.

The investigator is responsible for ensuring that changes to an approved trial, during the period for which IEC/IRB approval has already been given, is not initiated without IEC/IRB review and approval except to eliminate apparent immediate hazards to the subject.

5.5 Interruption of the Trial

The trial may be discontinued for administrative reasons or if new data about the investigational product resulting from this or any other trials become available, and/or on advice of the sponsor, the investigators, and/or the IEC/IRBs. If a trial is prematurely terminated or suspended, the sponsor shall promptly inform the investigators, the Regulatory Authorities and the IEC/IRBs of the reason for termination or suspension.

If the trial is prematurely terminated for any reason, the investigator should promptly inform the trial subjects and should ensure appropriate therapy and follow-up for subjects.

6 Treatments

6.1 Vaccines Administered (*Amendments 1, 2 and 3*)

Each subject will receive two doses of vaccine in the primary vaccination series (D0 [V01] and D21 [V02]) and a booster vaccination at either 6 months or 22 months (*Amendments 2 and 3*) after the first vaccination, or no booster (see Trial Flow-Chart [Figure 1, Figure 2,

Figure 3 and Figure 4 in synopsis]).

6.2 Identity of Investigational Product

6.2.1 Composition

The vaccines for primary and booster vaccinations (7.5 µg HA without adjuvant for A/Vietnam or 30 µg HA with aluminum hydroxide adjuvant [600 µg aluminum] for both A/Vietnam and A/Indonesia) will be presented in ready-to-use multidose vials containing 10 x 0.5 mL doses.

Two vaccine strains will be used as described in Section 5.1.1.

All vaccines contain 45 µg of Thiomersal per dose.

6.2.2 Preparation and Administration

Preparation

Vial must be gently shaken before use to obtain homogeneous suspensions. Before injection, the products will be placed at room temperature for few minutes and must be gently shaken before use to obtain homogeneous suspensions. A 0.5 mL volume of the product should be withdrawn from the multidose vial with a sterile syringe with a needle of 25 G. A new 25 G needle will be used for each injection.

A partially used vial will not be re-used for the next vaccination day. (*Amendment 1*).

Administration

The vaccines (0.5 mL per dose) will be injected intramuscularly into the deltoid region (it is recommended to use the opposite side to that of the blood sampling) using a sterile syringe with a needle of 25 G.

The IM injection is made at the center of the deltoid muscle between the shoulder and axilla and between the back and front of the arm. Before injection, the syringe plunger will be drawn back in order to ensure that the injection is not administered intravascularly.

The site and side of injection will be recorded in the source document and in the CRF.

6.2.3 Precautions for Use

Vaccination must not be performed in subjects allergic to one of the constituents of the vaccine (particularly egg and chicken proteins).

As after any vaccination, subjects must be kept under observation for 30 minutes after each injection to ensure their safety. Appropriate equipment (e.g. adrenaline, corticosteroids) must be available on site in case of immediate allergic reactions.

All study products should be inspected visually for cracks, broken seals, correct label content (see Section 6.5.1) and extraneous particulate matter and/or discoloration prior to administration whenever solution and container permit. If any of these conditions exists, the vaccine should not be administered and a replacement dose may be used.

6.2.4 Dose Selection and Timing

The selection of formulation, dose and schedule of administration are based on the results of GPA01 (12), as described in Section 1.3 and Section 5.2.

6.3 Identity of Control Product

Not applicable.

6.4 Identity of Other Products

Not applicable.

6.5 Product Logistics

6.5.1 Labeling and Packaging

Ready-to-use multidose vials will be presented in an individual box. The vial and the box will bear the following information: study name, product name and dosage, storage temperature, injection route, Sponsor's name, expiry date and legal information. Additional detachable labels bearing the same information will be provided. One label will be affixed to the source document and two labels will be affixed in the CRF of each subject vaccinated with the vial.

6.5.2 Storage and Shipment Conditions

Storage:

Products must be kept in a secure place with restricted access. Vaccines will be stored at a temperature ranging from +2°C to +8°C (in a refrigerator). The temperature must be monitored and documented on the appropriate form (see the Operating Guidelines) during the entire duration of the trial. In the event of accidental deep freezing or disruption of the cold chain, vaccines must not be administered; and the investigator or the responsible person should contact the Clinical Trial Manager (CTM) for further instructions.

Shipment:

The CTM will determine with the investigator or the designee in charge of product management, the dates and times of delivery of products and forward the information to the Sponsor's Packaging Department for Clinical Trial Lots. Products will then be shipped to the center according to the predetermined schedule.

The person in charge of the Packaging Department for Clinical Trial Lots will issue a dispatch note with acknowledgement of receipt attached to the package.

On delivery of the products to the site, the investigator or designee in charge of product management will check that the cold chain was maintained during shipment (verification of TESTO temperature recorder, freeze watch, and cold chain monitoring card). In the event of a problem, he/she should alert the CTM immediately.

The acknowledgement of receipt will be signed and dated by the person in charge of product management and will be sent according to the instructions given in the operating guidelines, together with the TESTO recorder.

6.5.3 Product Accountability

The investigator will be personally responsible for product management or will designate a person who will be responsible for product management.

The Sponsor's monitoring staff will verify each trial site's product accountability records versus the record of administered doses in the CRFs.

The investigator or the designee in charge of product management will maintain records of product delivery to the trial site, product inventory at the site, doses given to each subject, and the return of unused doses to the Sponsor.

The Investigator should alert the Sponsor as soon as possible of any expected or potential shortage of product doses during the trial so that the Sponsor can organize the shipment of extra doses.

6.5.4 Replacement Doses

Not applicable.

6.5.5 Return of Unused Products

Unused products will be returned to the Sponsor at the end of the vaccination period together with the form ‘Return slip of investigational products from the investigator site’, in accordance with the CTM's instructions. Empty boxes and partially used products will be destroyed on site after monitoring by the CTM. The destruction will be documented on site.

If a certificate of destruction on site is not available, the empty boxes and partially used products will be returned to the Sponsor at the end of the vaccination period.

6.6 Randomization/Allocation Procedures (*Amendments 1 and 3*)

At V01 (D0), after verification of inclusion and exclusion criteria, each subject will be assigned an inclusion number consisting of 8 digits. The first 3 digits will correspond to the center number, and the last 5 digits will correspond to the chronological order of enrolment **within each age group** (18 to 60 years and >60 years); the two groups of digits will be separated by a hyphen.

For example, the inclusion number of the first subject in Center 1, in the 18 to 60 year age group will be 001-00001. For subjects in the >60 year age group, the order of enrolment will commence at 201, and so, for example, the inclusion number of the first subject in Center 1 in the >60 year age group will be 001-00201. This convention will allow the randomization of more than 100 subjects in each age group.

A randomization list will be prepared by the Sponsor’s Biostatistics platform for the primary vaccination (for subjects enrolled prior to Amendment 1), and the following two factors will be randomized simultaneously:

- The two vaccine formulation groups.
- Blood sample aliquots for additional tests (neuraminidase, HI turkey, and ELISA).

The list will be created using the block permutation method, stratified by age group within centers. This guarantees, at any time, a similar number of subjects between the two vaccine formulation groups inside each center and each age group.

Additionally the size of the blocks will be adapted to also handle the randomization of blood sample aliquots for additional serological tests for 160 subjects (HI assay using turkey erythrocytes, neuraminidase, ELISA method). This will guarantee a similar 25% proportion of subjects with additional serological results within each vaccine formulation group, each center and each age group.

One scratchable randomization list will be provided for each age group and each center.

Once assigned, the subject number cannot be reassigned to another subject, even if the subject discontinues the trial before vaccination.

For the A/Indonesia booster, no randomization will be performed, as only subjects included in Centers 1 and 3 and receiving the 30 µg HA with adjuvant formulation at D0 and D21 will receive the A/Indonesia booster.

6.7 Blinding and Code Breaking Procedures

NA.

6.8 Concomitant Therapy

Ongoing medication, i.e., medication started before enrolment and continuing at the time of enrolment, will not be recorded in the CRF at V01, with the exception of the two categories described below and taken before the enrolment on the day of enrolment.

At V02 and V03 (including additional subjects) and at V04 and V05 for booster at 6 months or V05, V05_Add and V06 for A/Indonesia booster, the following two categories of medication (non-allowed therapies and antipyretics/analgesics/non steroidal anti-inflammatory drugs [NSAIDs]) taken since the previous visit will be recorded in the CRF, in the concomitant medication module.

- Non-allowed therapies:
    - Immunosuppressive therapy such as anti-cancer chemotherapy or radiation therapy or long-term systemic corticosteroid therapy.
    - Blood or blood derived products.
    - Vaccination other than with the trial vaccine.
- Antipyretics/analgesics/NSAIDs (considered as a single category).

For each reported medication, the following will be documented:

- Trade name.
- Medication category (one of the above categories will be ticked).
- Given as treatment or as prophylaxis.
- Start and stop dates.

Dosage, indication, and administration route will not be recorded in the CRF. Homeopathic medication will not be recorded in the CRF.

Medication given in response to an AE will be captured in the "Action Taken" column of the AE. No details will be recorded in the concomitant medication module of the CRF unless the medication received belongs to one of the two prelisted categories.

In the event of a SAE, all therapies taken should be reported in the initial and follow up SAE reporting forms, including the dosage, indication and route of administration information.

All therapies taken will be collected in the source document.

6.9 Treatment Compliance

The following measures will ensure that the treatment administered complies with the treatments planned, or that any non-compliance is documented so that it can be accounted for in the data analyses. All treatments will be administered by trial personnel. The investigator or the person in charge of product management will maintain records of product delivery to the trial site, product inventory at the site, dose given to each subject, and the return of unused doses to the Sponsor. Detachable labels will be affixed in the source document, in the CRF and on the product dispensing list if needed after administration of each dose of vaccine. If not possible, the dose number must be written by hand in the space provided for the label. The injection group will be recorded in the CRF.

7 Specimens and Clinical Supplies

See Section 5.1 and the Trial Flowchart (Figure 1, Figure 2,

Figure 3 and Figure 4) for details of the sampling schedule.

Subjects will have the opportunity to authorize the use of their blood samples for tests related to this study but not for future use for any other research in the Informed Consent Form.

7.1 Management of Samples for Humoral Immune Response

7.1.1 Sample Collection for Serological Assessments

For serology assessments, 30 mL of blood will be collected in dry tubes at each visit (except for the safety follow-up visit/telephone call).

If blood is not drawn by the investigator, then immediately prior to drawing blood, the person in charge of the procedure will verify the subject's identity. Each tube of blood will be clearly labeled with the subject identification number and the sampling stage using a self-adhesive label that will be stuck onto the tube immediately before blood sampling.

7.1.2 Sera Preparation of Blood Samples for Serological Assays

Sample preparation procedures will be described in detail in the Operating Guideline instruction manual provided to the investigator's team.

The sampling tube should be stored at room temperature for a minimum of 60 minutes and a maximum of 2 hours after sampling and before centrifugation. The tube must be stored vertically and must not be shaken.

Beyond 2 hours, the sampling tube is to be placed at a temperature between 2°C and 8°C and must be centrifuged within a maximum of 24 hours.

After being allowed to clot at room temperature, blood samples will be centrifuged before being divided into appropriate aliquots of serum. Samples will then be handled one subject at a time to avoid the mix-up of the subjects' blood tubes. Serum will be transferred to the appropriate number of tubes after the tubes have been labeled with self-adhesive labels that clearly identify the subject inclusion number and sampling stage or visit number.

The subject inclusion number, the date of sampling, and the number of primary tubes and retention tubes obtained will be specified on a sample identification list. Comments may be made on the quality of samples in the space provided on this list.

Serum aliquots will be frozen immediately at ≤-17°C until testing.

7.1.3 Sera Storage and Shipment of Samples for Serological Assays (*Amendment 1*)

Serum tubes will be stored frozen at ≤-17°C and shipped frozen to the appropriate laboratory. The storage temperature will be monitored and documented on the appropriate form (the operating guidelines will provide further details) during the entire trial.

Testing of samples for the A/Vietnam primary vaccinations will be performed in the following laboratories:

- For HIT in a subset of 25% of subjects: [redacted] UK.
- HIH and SN: [redacted] UK.
- Neuraminidase assay in a subset of 25% of subjects: [redacted] France.
- ELISA: [redacted] France.

- CMI[redacted] France.

Testing of all samples since V04_Add will be performed in the following laboratory (*Amendments 2 and 3*):

- HIH and SN[redacted] USA.

7.1.4 Retention of Unused Serum Samples for Repository (Humoral) (*Amendment 1*)

For each blood sample drawn for serum Ab response assessment, a maximum of 15 serum aliquots of 1 mL will be obtained.

For samples from the A/Vietnam primary vaccinations:

- one will be used for HI testing[redacted] for a subset of subjects.
- one will be used for HI and seroneutralization[redacted]
- two will be used for the neuraminidase inhibition test[redacted] for a subset of subjects.

For all samples since V04_Add (*Amendments 2 and 3*):

- one will be used for HI (Horse erythrocytes) and seroneutralization[redacted].

Any remaining serum for all samples will be kept in the serum bank at the[redacted] laboratory. This additional serum sample may be used in the event of insufficient volume of serum in the tubes planned for the analyses. If not used for replacement, these stored samples may be used for other evaluation studies, e.g. studies of the immune response to influenza vaccines, improvement of knowledge and documentation of the safety of this vaccine by using new developed laboratory methods or applying newly discovered concepts. In such cases, samples will be anonymized. No genetic studies will be performed. Subjects will have an option in the informed consent form to accept or refuse such future use of their samples.

7.2 Management of Samples for Cellular Mediated Immune Response

7.2.1 Sample Collection for Cellular Mediated Immune Response Assessments

For CMI assessments, 20 mL of blood will be collected in sodium heparine tubes from a subset of 80 subjects per formulation group (40 subjects per age group at Center 4 only).

If blood is not drawn by the investigator, then immediately prior to drawing blood, the person in charge of the procedure will verify the subject's identity. Each tube of blood will be clearly labeled with the subject identification number and the sampling stage using a self-adhesive label that will be stuck onto the tube immediately before blood sampling.

7.2.2 Peripheral Blood Mononuclear Cell Purification for Cellular Mediated Immune Response Assessment

Sample preparation procedures will be described in detail in the Operating Guideline instruction manual provided to the investigator's team.

The sampling tube should be stored at room temperature under smooth agitation for a maximum of 4 hours before processing.

Purification will be performed according to a standard (non-validated) protocol as follows:

Ficoll (15 mL) will be added to a 50 mL Leucosep tube and centrifuged for 5 minutes at 700 g to transfer the Ficoll below the membrane. 20 mL of heparinized blood will be added to the tube and the tube will be centrifuged at room temperature at 700 g for 20 minutes with no brake. Rings of mononuclear cells floating at the interface between plasma and Ficoll will be collected in 50 mL tubes. Mononuclear cells will be washed once for 10 minutes at 400g with physiological saline (NaCl 0.9%) between 2°C (35.6°F) and 8°C (46.4°F), and twice for 10 minutes at 400g with 50 mL pre-refrigerated medium. Cell pellets will finally be re-suspended in 2 mL of cold freezing medium (90% heat inactivated FCS + 10% dimethyl sulfoxide, Sigma) and transferred into two cryostat Nunc tubes (Merck/Polylabo) kept on ice and labeled in advance. Tubes will then be transferred in a cold Nalgene box and placed at -80°C (-112°F) for at least 16 hours before being moved to liquid nitrogen tanks.

7.2.3 Peripheral Blood Mononuclear Cell Storage and Shipment of Samples for Cellular Mediated Immune Response Assessment

For each blood sample for CMI assessment two aliquots of peripheral blood mononuclear cells (PBMC) will be obtained. Tubes will be kept in liquid nitrogen tanks. They will be transferred [redacted] in nitrogen containers provided by sanofi pasteur [redacted]

[redacted]
France

The shipment will be organized in accordance with the requirements applicable for the air transport of infectious substances (International Air Transport Association 6.2 regulations).

7.3 Clinical Supplies

The Sponsor will provide the protocol, informed consent forms, addenda to ICFs, CRFs, DCs, MAs, operating guidelines, and trial material such as temperature recorder TESTO (which will be retrieved at the end of the trial), rulers, thermometers, and syringes/needles for vaccine administration. Blood drawing material (except for that used for taking the samples for CMI analysis at Center 4) (e.g. dry silicon tubes, needles) will be provided by the investigator.

8 Assessments Methods and Endpoints

8.1 Immunogenicity Endpoints and Assessments Methods

8.1.1 Endpoints

Primary series

Hemagglutination inhibition assays will be performed using:

1. Horse erythrocytes for all subjects.
2. Turkey erythrocytes for a subset of 80 subjects in each formulation group (40 subjects per age sub-group) for one vaccine strain only - A/Vietnam (*Amendment 1*).

Anti-hemagglutinin (anti-HA) titers against the A/H5N1 strain will be expressed as described below:

- Anti-HA titer obtained in duplicate on D0, D21, and D42, and summarized at the subject level by individual geometric means of duplicates at each timepoint. The following endpoints will be derived:
  - Individual titer ratios D21/D0, D42/D0, and D42/D21.
  - Proportion of subjects with anti-HA Ab titer ≥40 (turkey) or ≥32 (horse) 1/dilution (dil) on D0, D21 and D42.
  - Seroconversion (for subjects with a titer <10 [1/dil] [turkey] or <8 [horse] on D0: post-injection titer ≥40 [turkey] or ≥32 [horse] [1/dil])

  or

  - significant increase (for subjects with a titer ≥10 [turkey] or ≥8 [horse] [1/dil]: ≥4-fold increase of the titer) at D21 and D42.

AND

Neutralizing Ab titers against the A/H5N1 strain will be expressed as described below.

- Neutralizing Ab titer obtained in duplicate on D0, D21, and D42 and summarized at the subject level by individual geometric mean of duplicates at each timepoint. The following endpoints will be derived:
  - Individual titer ratios D21/D0, D42/D0, and D42/D21.
  - 2- and 4-fold increase from D0 to D21 and to D42.

Antibody persistence

Anti-HA Ab titer (HIH method) and neutralizing Ab titer (SN method) at M6 (D180) (for all subjects), and at M15 and M22 (for a subset of subjects).

Booster vaccination response ***(Amendment 3)***

An A/Vietnam will be used as the booster strain for 6-month booster, and an A/H5N1 A/Indonesia will be used as the booster strain at 22 months (see Section 5.1.1). The booster Ab

response will be assessed against the homologous strain in the vaccine. In addition, antibody response to heterologous strains used in the booster vaccination may be tested.

- Anti-HA antibody titer obtained in duplicate at M6 (D180) and M6+21D (D201) (if 6-month A/Vietnam booster) or M22 (D670), M22+7D (D677) and M22+21D (D691) (if 22-month A/Indonesia booster), and summarized at the subject level by individual geometric means of duplicates at each timepoint. The following endpoints will be derived:
  - Individual titer ratios M6+21D/M6 if 6-month A/Vietnam booster or M22+7D/M22 and M22+21D/M22 if 22-month A/Indonesia booster.
  - Proportion of subjects with anti-HA Ab titer ≥32 (1/dil) at M6 and M6+21D if 6-month A/Vietnam booster, or M22, M22+7D and M22+21D if 22-month A/Indonesia booster.
  - Seroconversion (for subjects with a titer <8 [1/dil] at M6 or M22: post-injection titer ≥32 [horse] [1/dil]) at M6+21 days if 6-month A/Vietnam boosteror M22+7 days and M22+21 days if 22-month A/Indonesia booster,

  or

  - significant increase (for subjects with a titer ≥8 [1/dil]: ≥4-fold increase of the titer) at M6+21D if 6-month A/Vietnam booster, or M22+7D and M22+21D if 22-month A/Indonesia booster.

AND

Neutralizing Ab titers against the A/H5N1 strain will be expressed as described below.

- Neutralizing Ab titer obtained at M6 and M6+21D if 6-month A/Vietnam booster, or M22, M22+7D and M22+21D if 22-month A/Indonesia booster,. The following endpoints will be derived:
  - Individual titer ratios M6+21D/M6 if 6-month A/Vietnam booster, or M22+7D/M22 and M22+21D/M22 if 22-month A/Indonesia booster.
  - 2- and 4-fold increase from M6 to M6+21D if 6-month A/Vietnam booster, or M22 to M22+7D and from M22 to M22+21D for 22-month A/Indonesia booster

8.1.2 Observational Endpoints

Cellular Mediated Immune Response

Secretion of a panel of Th1 (IFN-gamma, TNF-alpha, IL-2), and Th2 (IL-5, IL-4, IL-13) cytokines by peripheral blood mononuclear cells (PBMCs), upon *in vitro* re-stimulation with vaccine antigens will be quantified before the first vaccination and 21 days after the second vaccination.(Amendment 3).

Neuraminidase Inhibition Test

Anti-neuraminidase Ab titer on D0 and D42.

- Anti-neuraminidase titer obtained on D0 and D42 in a subset of 80 subjects in each formulation group (for subjects who receive A/Vietnam in the primary vaccination series only) (*Amendment 1*); and summarized at the subject level by individual geometric mean of duplicate at each timepoint. The following endpoints will be derived:

- Individual titer ratios D42/D0.
- 2- and 4-fold increase from D0 to D42.

Anti HA Response by ELISA

- Anti- HA antibody titers against the A/H5N1 strain will be expressed in duplicate at least on D0 and D42 in a subset of 80 subjects in each formulation group (for subjects who receive A/Vietnam in the primary vaccination series only) (*Amendment 1*) (depending on GPA01 results).
    - Individual titer ratios D42/D0.
    - 4-fold increase from D0 to D42.

8.1.3 Assessment Methods

8.1.3.1 Hemagglutination Inhibition Test

The HI test adapted to the avian strain will be performed. The principle of the HI test is based on the ability of specific anti-influenza antibodies to inhibit hemagglutination of horse or turkey red blood cells (RBC) by influenza virus HA. The sera to be tested have to be previously treated to eliminate the non-specific inhibitors and the anti-species HAs.

Serum Pre-treatment

- Elimination of non-specific inhibitors by incubation of the unknown serum samples and quality control sera (serum of ferret or human immunized with influenza virus) with neuraminidase (18 hours at +36°C to +38°C in an incubator and 1 hour at +56°C in a water-bath).
- Absorption of spontaneous anti-species agglutinins by incubation of the serum samples and quality control sera from which non-specific inhibitors have been already removed, with the RBC suspension (2 hours at +2°C to +8°C). After these steps, the mixtures are centrifuged (10 minutes at 700g) and the supernatants are submitted to the HI method.

HI Test

For HI [turkey] 12 two-fold dilutions starting at 1:10 dilution of serum sample (or quality control sera) are performed and incubated with the HA antigen suspension (previously titrated to adjust the dilution at 4 HAU [HA units]/25 µL). The HA antigen is not added to the well dedicated to each serum quality control. For HI [horse] the starting dilution is 1:8.

The mixture is incubated for 1 hour at room temperature and 25 µL of the 0.4% RBC suspension are added. The reaction is left for 1 hour at room temperature before reading.

Reading

The serum titer is equal to the highest reciprocal dilution, which induces a complete inhibition of hemagglutination. The titer of each quality control serum is close to the previously assigned value (within one serial two-fold dilution limits).

The RBC control (RBC suspension without antigen) and the serum control (for each sample tested) do not produce any agglutination.

Each serum sample is titrated in duplicate. The final titer is equal to the geometric mean of the two results, which should not differ by more than a two-fold serial dilution.

8.1.3.2 Seroneutralization

The influenza virus microneutralization test is a specific assay for antibodies to the avian influenza A (H5N1) virus in human serum and could potentially be used to detect antibodies to other avian subtypes. This microneutralization test is more sensitive than the HI assay (the traditional assay for antibodies to human influenza A and B viruses), as this assay can detect H5-specific Ab in human serum at titers that can not be detected by the HI assay.

Inactivated human serum samples are pre-inoculated with a standardized amount of virus prior to the addition of Madin-Darby canine kidney (MDCK) cells. After overnight incubation, Enzyme Linked Immunosorbent Assay (ELISA) is used to measure the viral NP protein in infected MDCK cells. Since serum antibodies to the influenza virus HA inhibit the viral infection of MDCK cells, the optical density results of the ELISA are inversely proportional to the serum Ab concentration.

8.1.3.3 Cellular Mediated Immune Response

The CMI will be performed by [redacted] The technology used is under development and therefore is not validated.

Thawed PBMC will be stimulated for 6 days with vaccine antigen. At D6, supernatants of each timepoint of each subject will be collected and tested for their cytokine (interleukin-2 [IL-2], IL-4, IL-5, IL-13, interferon-γ [IFN-γ], tumor necrosis factor-α [TNF-α]) contents using standard commercially available kits based on Luminex technology, according to the manufacturer's protocol. Results will be expressed in pg/mL.

8.1.3.4 Neuraminidase Inhibition Test

The methods to be used for the neuraminidase inhibition test are described by Aymard-Henry et al (16).

8.1.3.5 ELISA Tests

The ELISA assay will be performed using purified HA from H5N1 strain obtained from Protein Science (whole HA protein produced in baculovirus).

Overnight, 96-well microplates will be coated at 4°C with purified HA. Plates will then be blocked for 1 hour at 37°C with 150 µL/well of PBS pH 7.1, 0.05 % Tween 20, 1 % (w/v) powdered skimmed milk (DIFCO) (PBS-Tween milk). All following incubations will be carried out in a final volume of 100 µL, followed by 4 washings with PBS pH 7.1, 0.05 % Tween 20.

Serial two-fold dilutions of serum samples will be performed in PBS-Tween-milk, starting from 1/100 added to the wells and incubated for 90 min at 37°C. After washing, anti-human IgG peroxydase conjugate will be added and the plates will be incubated for 90 min at 37°C. Plates are

further washed and incubated in the dark for 20 min at room temperature with 100 µL per well of a ready-to-use TMB substrate solution. The reaction is stopped with 100 µL per well of 1M HCl.

The optical density (OD) is measured at 450 nm to 650 nm with a plate reader (Spectra Max). The IgG antibody titers are calculated using the Soft max pro software, for the OD value range of 0.2 to 2.0, from the titration curve (standard serum included in each plate). All final titers were expressed in $\log_{10}$ (Log).

8.1.4 Handling of Missing Data and Outliers

Missing or incomplete data will not be replaced, with the exception of the following:

- All values strictly under the lower limit of quantification (LLOQ) will be treated as LLOQ/2.
- All values above or equal to the upper limit of quantification (ULOQ) will be truncated as ULOQ.

These above replacements of data may have an impact on most parameters.

No search for outliers will be done.

8.2 Efficacy Endpoints and Assessments Methods

No efficacy data will be obtained in the trial.

8.3 Safety Endpoints and Assessments Methods

8.3.1 Definitions

The following definitions are extracted from the ICH E2A Guideline for Clinical Safety Data Management: Definitions and Standards for Expedited Reporting.

Adverse Event:

An AE is any untoward medical occurrence in a patient or clinical investigation subject administered a pharmaceutical product and which does not necessarily have to have a causal relationship with this treatment. An AE can therefore be any unfavorable and unintended sign (including an abnormal laboratory finding, for example), symptom or disease temporally associated with the use of a medicinal product, whether or not considered related to the medicinal product.

The worsening of an existing sign of symptom is also considered as an AE. Therefore an AE may be:

- A new illness.
- The worsening of a concomitant illness.
- An effect of vaccination, including the comparator.
- A combination of the above.

Surgical procedures are not AEs; they are the action taken to treat a medical condition. It is the condition leading to the action taken that is the AE (if it occurs during the trial period).

Medical conditions leading to surgery that started prior to the trial but did not worsen during the trial are not to be reported as AEs.

Serious Adverse Event:

Serious and *severe* are not synonymous. The term *severe* is often used to describe the intensity (severity) of a specific event (as in mild, moderate, or severe myocardial infarction); the event itself, however, may be of relatively minor medical significance (such as severe headache). This is not the same as *serious* which is based on patient/event outcome or action criteria usually associated with events that pose a threat to a patient's life or functioning. Seriousness, not severity, serves as a guide for defining regulatory reporting obligations.

An SAE is any untoward medical occurrence that at any dose (including overdose):

- Results in death.
- Is life-threatening[a].
- Requires inpatient hospitalization or prolongation of existing hospitalization[b].
- Results in persistent or significant disability/incapacity[c].
- Is a congenital anomaly/birth defect.
- Is an important medical event[d].

Adverse Reaction (AR):

All noxious and unintended responses to a medicinal product related to any dose should be considered adverse drug reactions.

(The phrase "responses to a medicinal product" means that a causal relationship between a medicinal product and an AE is at least a reasonable possibility).

---

[a] *The term "life-threatening" refers to an event in which the patient was at risk of death at the time of the event; it does not refer to an event which hypothetically might have cause death if it were more severe.*

[b] *All medical events leading to hospitalizations will be recorded and reported as Serious Adverse Events, with the exception of: hospitalization planned before inclusion into the trial or out-patient hospitalization with no overnight hospitalization*

[c] *"Persistent or significant disability or incapacity" means that there is a substantial disruption of a person's ability to carry our normal life functions*

[d] *Medical and scientific judgment should be exercised in deciding whether expedited reporting is appropriate in other situations, such as important medical events that may not be immediately life-threatening or result in death or hospitalization but may jeopardize the patient or may require intervention to prevent one of the other outcomes listed in the definition above. These should also usually be considered serious. Examples of such events include allergic bronchospasm requiring intensive treatment in an emergency room or at home, blood dyscrasias or convulsions that do not result in inpatient hospitalization, or the development of drug dependency or drug abuse, new onset diabetes or autoimmune disease.*

Unexpected Adverse Drug Reaction (ADR):

An adverse reaction, the nature or severity of which is not consistent with the applicable product information (e.g. Investigator's Brochure for an unapproved investigational medicinal product).

The following additional definitions are used by the Sponsor:

Solicited Reaction

A solicited reaction is a term prelisted in the CRF**.** The assessment of these events post-vaccination is mandatory. A solicited event is defined by a combination of:

- symptom.
- onset post-vaccination.

e.g., injection site pain between D0 and D3 post-vaccination, or headache between D0 and D7.

A solicited reaction is therefore an AR observed and reported under the conditions (nature and onset) prelisted (i.e., solicited) in the CRF.

Unsolicited AE / ADR

An unsolicited AE is an observed AE that does not fulfill the conditions prelisted in the CRF in terms of symptom and/or onset post-vaccination, e.g., if headache between day 0 and day 7 is a solicited event (i.e., prelisted in the CRF), then a headache starting on day 7 is a solicited event, whereas headache starting on day 8 post-vaccination is an unsolicited event.

Injection Site Reaction

An injection site reaction[a1] is an ADR of and around the injection site. Injection site reactions are commonly inflammatory reactions.

Systemic AE

Systemic AEs are all AEs that are not injection site reactions. They therefore include systemic manifestations such as headache, fever, as well as local or topical manifestations that are not associated with the vaccination site, e.g., rash that is localized but that is not at the injection site.

8.3.2 Endpoints

- The occurrence, time to onset, number of days of occurrence, and severity of solicited (prelisted in the subject diary and Case Report Form [CRF]) injection site reactions and systemic reactions occurring within 7 days following each injection will be reported.
- The occurrence, nature (Medical Dictionary for Regulatory Activities [MedDRA] preferred term), time to onset, duration, severity, relationship to vaccination and seriousness of unsolicited (spontaneously reported) adverse events (AEs) within 21 days following each injection will be reported.

[a] All injection site AEs are considered to be related to vaccination and are therefore all *injection site reactions.*

- The occurrence of the following reactions (MedDRA Preferred Terms given in parentheses) in the 3 days following each injection will be more especially reported (as defined by the European Medicines Agency (EMEA) Note for Guidance [CPMP/BWP/214/96]):
  - Injection site induration >5 cm observed for more than 3 days.
  - Injection site ecchymosis (injection site bruising).
  - Rectal equivalent temperature >38°C for 24 hours or more (pyrexia).
  - Malaise.
  - Shivering (rigors).
- The occurrence, nature, time to onset, and relationship to vaccination of SAEs during the whole trial (including the 6-month follow-up period) will be reported.

8.3.3 Safety Assessment Methods

8.3.3.1 30-minute Observation Period

Subjects will be kept under observation for 30 minutes after each vaccination to ensure their safety. Any AE (either injection site reaction or systemic AEs) occurring during this 30-minute period will be recorded in either the solicited or unsolicited AE tables of the CRF, as appropriate. Should any SAE, occur during the 30 minute period, then the SAE should be reported to the Sponsor according to the procedure described in Section 9.1.

8.3.3.2 Reactogenicity (Solicited Reactions from D0 to D7 after each Vaccination)

After vaccination, the subjectwill be provided with a safety DC, a digital thermometer and a flexible centimeter ruler and will be instructed how to record the following items in the safety DC on the day of vaccination and daily for the next 7 days (i.e., D0 to D7).

- Maximum daily oral temperature
- Maximum daily measurement or maximum severity grade of all other solicited injection site or systemic reactions
- Date of last day of presence of the reaction if it lasted beyond D7
- Action taken for each event, if any (medication etc)

Table 8.1 and Table 8.2 present, respectively, the injection site and systemic reactions that will be prelisted in the safety diary cards and CRFs, together with the severity scales.

Table 8.1: Solicited Injection Site Reactions: Definitions, Terminology and Severity Scales

	**Injection site pain**	**Injection site erythema**	**Injection site swelling**	**Injection Site Induration**	**Injection Site Ecchymosis**
**Diary Card term***	Pain	Redness	Swelling	Hardening	Bruising
**Definition**	See severity scale	Presence of a redness including the approximate point of needle entry	Swelling at or near the injection site<br>Swelling or oedema is caused by a fluid infiltration in tissue or cavity and, depending on the space available for the fluid to disperse, swelling may be either soft (typically) or firm (less typical) to touch and thus can be best described by looking at the size of the swelling	Hardening at or near the injection site<br>Hardening is caused by a slow diffusion of the product in the tissue leading to a thick or hard area to touch at or near the injection site and thus can be best described by looking at the size of the hardening	Ecchymosis is the result of the diffusion of blood in the skin from ruptured blood vessels that forms a purple or black and blue spot on the skin. It can be best described by looking at its size
**Severity scale†**	Mild = Easily tolerated<br>Moderate = Sufficiently discomforting to interfere with normal behavior or activities<br>Severe = Incapacitating, unable to perform usual activities, may have/or required medical care or absenteeism	Mild: < 2.5 cm<br>Moderate: ≥2.5 to <5<br>Severe ≥5 cm	Mild: < 2.5 cm<br>Moderate: ≥2.5 to <5<br>Severe ≥5 cm	Mild: < 2.5 cm<br>Moderate: ≥2.5 to <5<br>Severe ≥5 cm	Mild: < 2.5 cm<br>Moderate: ≥2.5 to <5<br>Severe ≥5 cm

* These terms are the ones that will appear in the safety DC

† For erythema, swelling, induration and ecchymosis, this information is provided for information only. The classification as mild moderate or severe will be done by the Sponsor's statistical department. The maximum daily measurement of the reaction will be reported in the safety DC and then in the CRF. The severity scale for the pain will be provided in the DC and CRF for ease of reference.

Table 8.2: Solicited Systemic Reactions: Definitions, Terminology and Severity Scales

	Fever	Headache	Malaise	Myalgia	Shivering
**Diary Card term***	Temperature	Headache	Feeling unwell	Muscle aches and pains	Shivering
**Definition**	Fever is defined by a oral temperature of ≥37.5°C	A headache is pain or discomfort in the head, or scalp. Does not include migraine.	General ill feeling<br>Malaise is a generalized feeling of discomfort, illness, or lack of well-being that can be associated with a disease state. It can be accompanied by a sensation of exhaustion or inadequate energy to accomplish usual activities	Muscle aches and pains are common and can involve more than one muscle at the same time. Muscle pain can also involve the soft tissues that surround muscles. These structures, which are often referred to as connective tissues, include ligaments, tendons, and fascia (thick bands of tendons).<br>Does not apply to muscle pain at the injection site which should be reported as injection site pain.	Cold feeling
**Severity scale†**	Mild<br>≥37.5°C – 38.0°C Oral<br>Moderate ≥38.1°C – 39.0°C Oral<br>Severe ≥39.1°C Oral	Mild<br>Noticeable but does not interfere with daily activities<br>Moderate<br>Interferes with daily activities<br>Severe<br>Prevents daily activities	Mild<br>Noticeable but does not interfere with daily activities<br>Moderate<br>Interferes with daily activities<br>Severe<br>Prevents daily activities	Mild<br>Noticeable but does not interfere with daily activities<br>Moderate<br>Interferes with daily activities<br>Severe<br>Prevents daily activities	Mild<br>Noticeable but does not interfere with daily activities<br>Moderate<br>Interferes with daily activities<br>Severe<br>Prevents daily activities

* These terms are the ones that will appear in the safety DC

† For fever, this information is provided for information only. The classification as mild moderate or severe will be done by the Sponsor's statistical department. The maximum daily body temperature will be recorded in the DC and then in the CRF. Severity scales for the other reactions will be provided in the DC and CRF for ease of reference.

Important notes for the accurate assessment of fever

Fever is defined, clinically, as a rectal temperature of 38.0°C or more. However for practical reasons, fever will be defined in this trial as oral temperature of 37.5°C or more. Tympanic thermometry must not be used.

Whatever the route used, the investigator will record in the CRF the temperature measured **without** applying any conversion factor. The CRF and the DC will provide the possibility to specify the temperature measurement route used in case it is not the requested route. Temperature should be measured once per day, at the same time each day preferably in the evening, and at the time of any apparent fever. The highest observed daily temperature should be recorded in the DC.

For the statistical analysis, results will be presented as rectal temperature.

8.3.3.3 Adverse Events from D0 to D21 after each Vaccination

Subjects will also be instructed to record any other medical events that may occur between D0 and D21 after each vaccination. Space will be provided in the DC for this purpose. For each event, the following will be recorded:

- Start and stop dates.
- Severity of the event according to the following scale:

  Mild: Noticeable but does not interfere with daily activities.

  Moderate: Interferes with daily activities.

  Severe: Prevents daily activities.
- Action taken for each event, if any (medication etc).

AEs likely to be related to the product (or to the experiment[a]), whether serious or not, which persist at the end of the trial will be followed up by the investigator until their complete disappearance or the stabilization of the subject's condition. The investigator will inform the Sponsor of the date of final disappearance of the AE and will document it on a Data Clarification Form (DCF).

8.3.3.4 Transcription of Safety Information to the CRF; Causality Assessment

At each visit, the investigator will perform a physical examination and will interview the subject for any solicited reactions and unsolicited AEs recorded in the DC, as well as regarding any other AEs that may have occurred. All relevant data will be transcribed into the CRF according to the instructions provided with the CRF.

[a] European Directive 2001/21/EC, for trials conducted in Europe only

The ***action taken*** to treat a solicited reaction or any AEs will be classified in the CRF using the following scale:

0 = No action.

1 = Medication[a].

2 = Health care contact (defined as: physician/nurse telephone contact; physician/nurse evaluation; emergency visit/outpatient hospitalization or tel. contact).

3 = Health care contact and prescription of a new medication.

4 = Hospitalization (inpatient).

The Investigator will assess the ***causal relationship*** between each unsolicited AE[b] and vaccination as either not related or related, based on the following definitions:

0 Not related / No relationship: The AE is clearly/most probably caused by other etiologies such as subject's underlying condition, therapeutic intervention or concomitant therapy, or the delay between vaccination and the onset of the AE is incompatible with a causal relation, or the AE onset is before vaccination.

1 Related*:* There is a reasonable possibility that the AE was caused by the vaccine.
The expression "reasonable possibility" is meant to convey in general that there are facts (evidence) or arguments to suggest a causal relationship (ICH Guidelines. Clinical Safety Data Management E2A).

Serious Adverse Events (see Section 9.1 for information on SAE reporting)

Information on SAEs will be collected and assessed throughout the trial.

For each solicited reaction and unsolicited AE reported, the investigator will indicate whether it was an SAE. Any SAE occurring throughout the trial will be reported by the investigator using SAE Alert and SAE Reporting forms (the procedure for reporting SAEs is described in Section 9.1). Any relevant information concerning the SAE is to be reported on these forms, either on the initial form or on a follow-up form if the relevant information becomes available later (e.g. outcome, medical history, results of investigations, copy of hospitalization reports). The investigator will assess the causal relationship between the SAE and the product using the scale provided above.

8.3.4 Handling of Missing Data and Outliers

Missing safety data will not be replaced. All vaccinated subjects with safety data, and all safety data recorded in the CRFs will be included in the safety analyses. No search for outliers will be performed.

---

[a] Self-medication, such as over the counter medication.

[b] By convention, all solicited events will be considered as related to vaccination and referred to as reactions. For these reactions, the investigator's opinion will not be requested in the CRF.

9 Serious Adverse Events

9.1 Reporting of Serious Adverse Events

In order to comply with current regulations on SAE reporting to Health Authorities and to allow the Sponsor to conduct a detailed analysis of the safety of the developed products, the investigator will accurately document any SAE, in accordance with the notification deadlines stated below, to provide the Sponsor with all necessary information and, if requested by the Sponsor, to give access to source documents.

9.1.1 Initial Reporting by the Investigator (*Amendment 1*)

The investigator must report every SAE occurring during a subject's participation in the trial (or to the experiment[a]) within 24 hours to the Sponsor's Global Pharmacovigilance Department. The SAEs must be reported even if the investigator considers that the SAE is not related to the vaccine.The investigator must complete the "SAE Initial Reporting Form" and send it by facsimile to the following number and to the local CRA:

[redacted]

If it is not possible to fax the form, the investigator may send the completed "SAE Initial Reporting Form" by express mail to the following address:

[redacted] France

[redacted]

The SAE Initial Reporting Form contains the minimum information requested by The Food and Drug Administration (and by ICH guidelines to define a SAE file).

9.1.2 Follow-up Reporting by the Investigator (*Amendment 1*)

The investigator must complete the "SAE Follow Up Reporting Form" and send it to the Sponsor's Global Pharmacovigilance Department as soon as possible and in any case NO LATER THAN 5 WORKING DAYS OR 7 CALENDAR DAYS after becoming aware of the SAE. The SAE Follow Up Reporting Form will be sent by facsimile as described above for the SAE Initial Reporting Form.

The SAE Follow Up Reporting Form includes essential data to perform the medical assessment of the case.

Any further relevant information concerning the SAE that becomes available after the SAE Follow Up Reporting Form has been sent (e.g., outcome, precise description of medical history,

[a] European Directive 2001/20/EC, for trials conducted in Europe only

results of the investigation, copy of hospitalization report) must also be sent to sanofi pasteur's Global Pharmacovigilance Department as soon as possible.

The anonymity of the subjects must always be respected when forwarding this information.

9.1.3 Reporting of SAEs Occurring After Subject Trial Termination

Any SAE occurring after subject trial termination but likely to be related to the product (or to the experiment[a]) must also be reported by the investigator as soon as he/she is alerted of it. In such a case, the procedure to be followed to report the SAE to the sponsor is identical to that described above.

9.1.4 Causal Relationship

The causal relationship between the SAE and the product (yes or no) will first be evaluated by the investigator using the following definitions:

> ***0 Not related / No relationship***: The AE is clearly/most probably caused by other etiologies such as patient's underlying condition, therapeutic intervention or concomitant therapy, or the delay between vaccination and the onset of the AE is incompatible with a causal relation, or the AE onset before vaccination.
>
> ***1 Related****:* There is a reasonable possibility that the AE was caused by the vaccine.
> The expression "reasonable possibility" is meant to convey in general that there are facts (evidence) or arguments to suggest a causal relationship (ICH Guidelines. Clinical Safety Data Management E2A).

Then, according to the available information and the current medical knowledge, the Sponsor's Product Safety Officer will also assess the causal relationship to the product in collaboration with the CTL.

The decision to modify or discontinue the trial, or to break individual or all trial codes may be made after mutual agreement between the Sponsor and the investigator(s).

9.1.5 Reporting SAEs to Health Authorities and IECs/IRBs

The Sponsor will inform health authorities of any reportable SAE according to the local regulatory requirements. Reporting to the Health Authorities will be according to the Sponsor's standard operating procedures.

The Sponsor's Responsible Medical Officer (the Clinical Team Leader) will notify the investigators in writing of the occurrence of a reportable SAE. The investigators will be responsible for informing the IECs or IRBs that reviewed the trial protocol.

9.1.6 Additional Information

If it can help to analyze the SAE , a blood sample (5 mL in a dry tube) can be taken.

---

[a] European Directive 2001/20/EC, for trials conducted in Europe only

For reported deaths, the investigator should supply the Sponsor and the IEC/IRB with any additional requested information such as autopsy reports or terminal medical reports.

10 Data Collection and Management

10.1 Data Collection, CRF Completion

All clinical trial information will be reported by the investigator or a designated person on a CRF designed specifically for this trial and provided by the Sponsor. The Sponsor will provide all necessary tools and instructions to complete the CRF. All CRFs must be signed by the investigator. The Sponsor requires explanations for all missing information. Incorrect data must be crossed-out with a single line, then initialed and dated. Correction fluid or similar correction methods that mask the original data are not to be used. These rules also apply to the completion of SAE Initial Reporting Forms, SAE Follow-Up Reporting Forms, Data Correction Forms, and Complementary Information Forms.

To ensure the correct and consistent completion of the CRFs and the respect of the visit procedures, the CRF will contain the following information:

- All necessary scales for the rating of severity and causality of events (these scales are presented in Section 8.3.3).
- The trial flow chart.
- A list of specific procedures to be followed at each visit.

In addition, detailed guidance for the completion of the CRF will be provided in a separate document.

Standard individual safety diaries, specifically designed for this trial and provided by the Sponsor, will be used by the subjects to record daily safety information between D0 and D21 after each vaccination. These diaries will include prelisted terms (see Section 8.3) and areas for free text. Subjects will be provided with diaries, rulers (for measuring the size of injection site reactions) and standard digital thermometers, and will be instructed how to use them. Severity or intensity scales will be provided in the diaries.

At each visit, the investigator or designated person will transcribe the appropriate post-vaccination safety information (for the period since the previous visit) from the DC to the CRFs after interviewing the subject to ensure that the information is complete and correct.

10.2 Data Management

Data generated during the trial will be managed following two different processes, one related to clinical data, defined as all data reported in the CRF and all laboratory data, and one related to the data pertaining to SAEs (i.e., data reported by the investigator on the SAE Initial Reporting Forms, SAE Follow-Up Reporting Forms and Complementary Information Forms).

Clinical Data Management

During the trial, through regular data collection and monitoring, clinical data reported in the CRFs will be integrated into the clinical database under the responsibility of sanofi pasteur Clinical Data Management platform. For each batch of data, double entry, quality control and triggers to computerized logic and/or consistency checks will be systematically applied in order to detect errors or omissions. Medical safety data reviews may be performed several times during the course of the trial. Queries will be generated and submitted through Data Clarification Forms to the investigator for resolution. Each step of this process will be monitored through the implementation of individual passwords and regular backups to maintain appropriate database access and to ensure database integrity.

The validation of the immunogenicity data will be performed at the laboratory level following the laboratory's procedures. Information from the laboratories such as subject identifiers and dates or sample numbers will be checked for consistency before the integration into the clinical database. All immunogenicity data, with the exception of those from the observational endpoints, will be provided by GCI, entered into GCI LIMS and quality control checked. These data will then be exported to Clinical Data Management for integration into the clinical database.

After integration of all corrections in the complete set of data, and after the SAE information available from Clinical Data Management and Pharmacovigilance has been reconciled, the database will be locked and saved before being released for statistical analysis.

SAE Data Management

During the trial, data pertaining to SAEs reported on SAE Initial Reporting Forms, SAE Follow Up Reporting Forms and Complementary Information Forms will be integrated into the Sponsor's centralized Global Pharmacovigilance Department database.

Upon receipt of an SAE Initial Reporting Form or an SAE Follow-Up Form, the data will be entered into the Global Pharmacovigilance Department database after a duplicate check. Each SAE is assigned a case identification number. Entered data will be independently verified against the original SAE forms. All SAEs are then reviewed by the Product Safety Officer and the Clinical Team Leader. Complementary Information Forms are issued and sent to the investigator as required. Each SAE is reviewed, locked and approved in the Global Pharmacovigilance Department database before being reported to the relevant authorities as necessary. Any follow-up information concerning a locked and approved SAE will be incorporated and a new version of the SAE will be created.

The data on SAEs in the Clinical Data Management database will be reconciled with those in the Pharmacovigilance database.

11 Statistical Methods and Determination of Sample Size

Data in the Clinical Data Management Database will be analyzed by the Biostatistics platform of sanofi pasteur, France, with the SAS software, Version 8.2 or above (SAS Institute, Cary, North Carolina, USA).

An initial statistical analysis will be performed when all the data obtained 21 days after the second vaccination have been locked (first partial database lock), addressing the safety and immunogenicity objectives of the D0 to D42 period.

----For A/Vietnam booster at 6 months----

A second statistical analysis will be performed when all the data obtained 21 days after the M6 booster have been locked (second partial database lock) in order to address the immunogenicity and safety objectives for the A/Vietnam booster vaccination and the antibody persistence objective. *(Amendment 2)*.

----For A/Indonesia booster---

A third statistical analysis will be performed 21 days after the A/Indonesia booster vaccination to address the immunogenicity endpoints for the A/Indonesia booster , the Ab persistence endpoints (M15 and M22), and the primary series A/Indonesia vaccination for the additional 50 subjects. *(Amendments 2 and 3)*

A final statistical analysis will be produced to incorporate the data after the final 6-month safety follow-up.

The statistical analysis plan, covering all analyses to be performed on primary series vaccination data, will be written before the first partial database lock.

11.1 Statistical Methods

11.1.1 Hypotheses and Statistical Methods for the Safety Endpoints

11.1.1.1 Hypotheses

No statistical hypotheses will be tested.

11.1.1.2 Safety Analysis

The safety analysis will report the occurrence of solicited reactions and the incidence of unsolicited events over the safety observation period by vaccine formulation and by age group. The safety observation period is 21 days after each vaccination, and 7 days after each vaccination for the solicited reactions.

In order to avoid any under-estimation of the incidences, the number of subjects with documented safety will be used as denominator of the frequencies. For solicited reactions, safety summary parameters and EMEA safety criteria, 95% CIs of point estimates of proportion will be calculated using the exact binomial distribution (Clopper-Pearson method) for proportions (17).

Safety Combined for the 21 Days after the First Two Vaccinations and after each Separate Vaccination (Including Booster):

The analyses of safety will address the number and percentage of subjects, by vaccine formulation and by age group, experiencing injection site or systemic AEs until 21 days after each injection (solicited reactions from 0 to 7 days and unsolicited AEs/reactions until 21 days).

The number and percentage of subjects experiencing the following will be described by vaccine formulation and by age group:

- Each solicited and unsolicited (MedDRA preferred term) reaction or event after each injection.
- Each solicited reaction (from 0 to 7 days) after each injection according to severity, time to onset, and number of days of occurrence.
- Each unsolicited adverse reaction (MedDRA preferred term) (until 21 days) after each injection according to severity, time to onset, and duration.

Additionally, the number and percentage of subjects experiencing the following reactions (MedDRA Preferred Terms) in the 3 days following each injection will be reported by vaccine and age group after each injection (as defined by the EMEA Note for Guidance [CPMP/BWP/214/96]):

- Injection site induration >5 cm observed for more than 3 days.
- Injection site ecchymosis.
- Rectal equivalent temperature >38°C for ≥24 hours.
- Malaise.
- Shivering.

The number and percentage of subjects in each group experiencing SAEs will be collected throughout the study.

11.1.2 Hypotheses and Statistical Methods for the Immunogenicity Endpoints

11.1.2.1 Hypotheses

No statistical hypotheses will be tested for the immunogenicity endpoints analysis.

11.1.2.2 Statistical Analysis

11.1.2.2.1 EMEA Immunogenecity Criteria for Analysis

The point estimates and their 95% CI of the parameters corresponding to the following endpoints will be presented for each vaccine formulation and age group:

Anti-HA titers[a] (1/dil) with Turkey or Horse Erythrocytes:

- Proportion of subjects with anti-HA Ab titer ≥10 (turkey) or ≥8 (horse) (1/dil) on D0[b], D21, D42.
- Geometric Mean (GM) of titers on D0, D21, and D42.

[a] Individual geometric means of duplicates

[b] In order to check the baseline naïve immunogenicity

- GM of individual titers ratio (GMTR) D21/D0, D42/D0, and D42/D21.
- Proportion of subjects with anti-HA Ab titer ≥40 (turkey) or ≥32 (horse) (1/dil) at D0, D21 and D42.
- Seroconversion (defined as initially subjects with anti-HA Ab titer <10 [turkey] or ≥8 [horse] [1/dil] pre-vaccination titer, with a post-vaccination titer ≥40 [turkey] or ≥32 [horse] [1/dil]) or significant increase rate (defined as initially subjects with anti-HA Ab titer ≥10 [turkey] or ≥8 [horse] [1/dil] pre-vaccination titer, with at least a 4-fold increase in post-vaccination titer) from D0 to D21, D0 to D42 and from D21 to D42.

Seroneutralization (1/dil):

- Proportion of subjects with a neutralizing Ab titer ≥20 (1/dil) on D0[a], D21, D42.
- GM of neutralizing Ab titer on D0, D21 and D42.
- GMTR: D21/D0, D42/D0, and D42/D21.
- 2- and 4-fold increase rate from D0 to D21 and from D0 to D42.

11.1.2.2.2 Methods for Statistical Analysis

For All Titration Methods

Geometric Mean computation: Assuming that log10 transformation of the measurements follows a normal distribution, at first, the mean and 95% CI will be calculated on log10 measurements using the usual calculation for normal distribution, then antilog transformations will be applied to the results of calculations, in order to provide geometric means and their 95% CIs.

95% CIs computation will be performed according to:

- The normal approximate method for CIs of geometric means (GM of Titers and GM of Ratios).

- The exact binomial distribution (Clopper-Pearson method) for CIs of proportions (17).

For HI Methods

This analysis will allow the investigation of the following requirements (as defined by the EMEA Note for Guidance [CPMP/BWP/214/96]) for each group:

[a] In order to check the baseline-naïve immunogenicity

Table 11.1: EMEA Recommended Immunogenicity Criteria - EMEA Note for Guidance [CPMP/BWP/214/96])

	**Age**	
**Defined from D0 to D21 and from D0 to D42**	**18 to 60 years**	**>60 years**
**Seroconversion or significant increase rate***	>40%	>30%
**Geometric mean of individual ratios**	>2.5	>2
**Percentage of seroprotected subjects†**	>70%	>60%

* Expected to be identical to the seroprotection rate after the first vaccination, since all subjects should be naïve (seroconversion and significant increase endpoints for HI are defined in Section 11.1.2.2.1)

† Seroprotection rate (titer ≥40 [turkey] considered to be equivalent to ≥32 [horse] 1/dil)

11.1.2.2.3 Exploratory Statistical Analyses

For each serological method, the following may be performed as exploratory analyses:

- Compare antibody responses to primary series vaccination with either A/Vietnam or A/Indonesia antigens
- Assess and quantify the second vaccination relative effect.
- Assess the vaccination formulation (30 µg with adjuvant versus 7.5 µg without adjuvant) effect.
- Check any age-group heterogeneity.
- Check any center heterogeneity.
- Confirm any gender effect on the immunogenicity parameters.

The exploratory analyses will be detailed in the Statistical Analysis Plan prior to the first database lock.

11.1.2.2.4 Immunogenicity Observational Endpoints

The point estimates and associated 95% CI of the parameters corresponding to the following endpoints will be presented by vaccine and age group.

95% CI computation will be performed according to:

- The normal approximate method for CIs of geometric means (GM of Titers and GM of Ratios).
- The exact binomial distribution (Clopper-Pearson method) for CIs of proportions (17).

Anti-neuraminidase Response

- Anti-neuraminidase titer obtained in duplicate on D0 and D42 and summarized at the subject level by individual geometric mean of duplicate at each timepoint. The following endpoints will be derived:
  - GM of individual titers ratios D42/D0.
  - 2- and 4-fold increase rate from D0 to D42.

Anti- HA Antibody Titers using ELISA Assay

HIA using ELISA method will be implemented for at least a subset of 80 subjects in each formulation group; the timepoints are not yet defined but will include at least pre- and post-vaccination titers.

Anti-HA antibody titers using an ELISA assay against the HA of the A/H5N1 strain will be calculated for each timepoint and summarized at the subject level by individual geometric means of duplicates at each timepoint.

- GM of individual titers ratios between timepoints.
- GM of individual titers ratios between timepoints.

Pre- or Post-booster Ab Persistence:

- The kinetics of the Ab titers assessed by HIH method and SN will be studied and summarized graphically: GM anti-HA titers and GM of Ab titers, respectively at M6, M15 and M22, as applicable.

Booster Vaccination Immunogenicity:

For each serological method (HIH and SN) the analysis will be similar to, and simplified from, the main statistical analysis.

In addition, for the A/Indonesia booster, immunogenicity endpoints will be assessed 7 days after booster vaccination.

11.2 Population to be Analyzed

11.2.1 Definition of Populations

11.2.1.1 Full Analysis Set

The full analysis set (FAS) is defined for the immunogenicity analyses of the primo-vaccination, the Ab persistence, and the booster vaccination.

The FAS is defined as the subset of subjects who received the first injection.

The analysis of the primo-vaccination (see Section 11.1.2.2.1) addresses endpoints involving pre- and post-injection titers at D0, D21 and D42 for HI and SN methods. The analysis will include all available data.

Data from subjects excluded from this analysis set will be listed separately and the reasons for exclusion, as well as the circumstances of the missing data, will be examined.

Additionally, for the booster analysis, the FAS is defined as subjects having also received the booster injection.

The analysis will be performed according to the randomized vaccine formulation group.

11.2.1.2 Per-Protocol Analysis Set

The per-protocol analysis set (PPAS) is defined for the main immunogenicity endpoints.

Subjects presenting with at least one of the following conditions will be excluded from the PPAS:

- Inclusion and exclusion criteria not met or exclusion criteria met which may have an impact on immunogenicity (inclusion criterion 1, and exclusion criteria 6, 9, 10, and 11).
- Detected randomization error/wrong quantity injected or detected schedule inconsistent with the study design (e.g. two primary injections of different dosages).
- Definitive contraindication met which may have an impact on immunogenicity (contraindications 2, 3, and 5).
- Any injection not performed.
- Deviation from the planned dose schedule.
- Any blood sample not drawn.
- Deviation from blood sampling schedule (21±3 days after each injection).
- Any prohibited therapy received until third blood sample.

Conditions leading to exclusion from the per protocol analysis set will be detailed in the statistical analysis plan.

The PPAS is defined at D21 and D42 for the primo-vaccination. To be per protocol at D42, the subject must also be per protocol at D21. For each serological method, the D42/D0 and D42/D21 analyses will require all the D0, D21 and D42 serological results.

For the booster vaccination, the PPAS is similarly defined from the day of booster vaccination to 21 days after the booster vaccination.

Data from subjects excluded from this population will be listed separately.

11.2.1.3 Safety Analysis Set

The safety analysis set (SafAS) is defined as those subjects who received at least one dose of study vaccine. A safety analysis set is defined for each dose as the subset of subjects who received the dose.

Safety results will be presented according to the schedule received.

Subjects who received an inconsistent vaccination schedule (e.g. two primary injections of different dosages) will be assigned to the group determined by the first injection received. Their

safety results after the second injection will be excluded from all safety analyses, and will be listed separately.

The SafAS for the booster vaccination is defined as those subjects who received at least one injection of the primo vaccination.

11.2.1.3.1 Cellular Mediated Immune Response and Additional Serological Tests

The CMI subset will comprise all the subjects enrolled at Center 4 only. It is expected that a total of 80 subjects per vaccine formulation at this center (40 subjects per age sub-group) will provide blood samples for CMI analysis.

For additional serological assays (including HI assay using turkey erythrocytes, neuraminidase, and the ELISA test) a common random subset of 80 subjects in each formulation group (40 subjects per age sub-group) (*Amendment 1*) will be determined by the randomization.

Each analysis subset will be a sub-population of the FAS that has both the D0 or the D42 results available for the primo vaccination. This will guarantee a similar 25% proportion of subjects with additional serological results for each vaccine formulation group, each center and each age group.

11.2.2 Populations Used in Analyses

The populations to be used in the analyses are presented in the table below.

Table 11.2: Populations for Analyses

<table>
<tr><th>Objectives</th><th colspan="2">Description and Endpoints</th><th>Included and randomized subjects</th></tr>
<tr><td rowspan="2">Safety objectives</td><td colspan="2">Primo vaccination safety</td><td>SafAS</td></tr>
<tr><td colspan="2">Booster vaccination safety</td><td>SafAS</td></tr>
<tr><td rowspan="3">Immunogenicity objectives</td><td>Primo vaccinations
(A/Vietnam and A/Indonesia)</td><td rowspan="3">- HI (horse erythrocytes)
-Seroneutralisation</td><td>FAS and additionally PPAS</td></tr>
<tr><td>- Ab persistence</td><td>FAS (available titers at pre- and post-booster timepoint)</td></tr>
<tr><td>- Booster vaccinations
A/Vietnam (M6)
A/Indonesia (M22)</td><td>FAS and additionally PPAS
A/Vietnam (Center 2)
A/Vietnam (Center 1 and 3)</td></tr>
<tr><td rowspan="2">Immunogenicity observational objectives</td><td colspan="2">Neuraminidase
HI assay using turkey erythrocytes
HI assay using ELISA test (to be confirmed following GPA01 results)</td><td>160 subjects randomized subset of FAS subjects</td></tr>
<tr><td colspan="2">CMI response</td><td>CMI subset of FAS (Center 4)</td></tr>
</table>

11.3 Determination of Sample Size and Power Calculation

A total sample size of 300 subjects per formulation group (including the two sub-groups of 150 subjects by age in each formulation group) will provide a probability of 95% to observe a 1% incidence for any AE in any formulation group, and a probability of 78% to observe a 1% incidence of any AE in each formulation group.

For the A/Indonesia primary vaccination series, a sample size of 50 subjects was set arbitrary.

11.4 Interim Analysis

No interim analysis is planned.

12 Ethical and Legal Issues and Investigator/Sponsor Responsibilities

12.1 Ethical Conduct of the Trial / Good Clinical Practice

This trial will be conducted in accordance with the Edinburgh revision of the Declaration of Helsinki as far as adopted by the concerned regulatory authorities, as well as ICH Good Clinical

Practice, the applicable national and local requirements regarding ethical committee review, informed consent, and other statutes or regulations regarding the protection of the rights and welfare of human subjects participating in biomedical research.

12.2 Source Documents and Source Data

The purpose of source documents is to document the existence of the subject and substantiate the integrity of the trial data collected. The investigator must maintain the trial source documents accurate, complete, legible and up to date.

Examples of source documents are: Subject Contact, Screening, and Enrolment Log, Clinical and Office Charts, Subject's diaries, hospital records, informed consent forms, subject's file and records kept at the pharmacy or at the laboratories, mail, certified letters.

The Subject Contact, Screening, and Enrolment Log should list all subjects who contacted the investigator or were contacted by the investigators to participate in the trial, regardless of the outcome.

Source data are the data contained in source documents (originals or certified copies).

12.3 Confidentiality of Data and Access to Subject Records

Prior to initiation of the trial, the Investigator will sign a fully executed confidentiality agreement with the Sponsor.

Electronic medical records alone are acceptable only if validated computerized systems are used and are compliant with US 21 CFR Part 11. If this is not the case, the investigator is obliged to print any records on an on-going basis, to sign and date them immediately after their creation, and to keep them on file as source documents which can be verified by the Sponsor or an inspector in comparison to the electronic records. Any later changes of the electronic records require the record to be reprinted, dated (with an indication of the date of change) and signed. Such records must also be kept together with the original printed copy.

Sponsor personnel, the IRB/IEC and the regulatory authorities will have direct access to source data/documents.

12.4 Monitoring, Auditing, and Archiving

12.4.1 Monitoring

Before the start of the trial (i.e., before the inclusion of the first subject by the first center), the staff investigators and the Sponsor's monitoring staff will meet at the "Site-initiation visit" to discuss the trial protocol and the detailed trial procedures, with emphasis on inclusion and exclusion criteria, visit timing, safety procedures, informed consent procedures, SAE reporting procedures, CRF completion, and sample and product handling.

The Sponsor's monitoring staff will ensure and document that all material to be used during the trial has been received and that the investigational team and local monitoring staff have been

properly informed about the trial, GCP and regulatory requirements, and the Sponsor's procedures. Specific training sessions for the investigational team and local Clinical Research Assistants (CRAs) on these topics may be performed, as necessary.

Specific instruction manuals will be provided for the completion of the CRF (CRF Completion Guide) and for the detailed trial procedures such as the laboratory and sample handling procedures (Operating Guidelines).

After the start of the trial, the Sponsor's monitoring staff will be in regular contact with the investigational team through telephone calls and regular follow-up visits to the trial centers.

The investigator must be available for these visits and will allow the monitoring staff direct access to subject medical files and CRFs. During monitoring visits, the monitoring staff will:

- Control the quality of the trial progress (e.g., with respect to the protocol and operating guidelines, quality of data collection and document completion, signature of consent forms, appearance of SAEs, sample and product management, cold chain monitoring, and archiving).
- Collect completed CRFs or CRF pages and any corresponding queries (DCFs and Complementary Information Forms [CIFs]).
- Evaluate the number of complete or ongoing observations.

Any identified problems will be discussed with the investigator and corrective or preventive actions will be determined, as appropriate.

Once the CRF pages corresponding to the last visit have been returned duly completed and signed, the investigator must be available to complete any queries (DCFs and CIFs) forwarded by the Sponsor until database lock.

At the end of the trial, a close-out visit will be performed to ensure that:

- The center has all the documents necessary for archiving.
- All samples have been shipped to the appropriate laboratories.
- All unused material and products have been returned to the Sponsor.

12.4.2 Audits and Inspections

A quality assurance (QA) audit may be performed by the Sponsor's QA Department or by independent auditors to verify that the trial has been conducted according to the protocol, GCP, ICH requirements and the applicable regulations. An inspection may be conducted by regulatory authorities. The investigator must allow direct access to trial documents during these inspections and audits.

12.4.3 Archiving

The investigator must keep all trial documents for at least 15 years after the completion or discontinuation, whatever the nature of the investigational center (private practice, hospital, institution). The investigator will inform sanofi pasteur of any address change.

The Sponsor, or subsequent owner, will retain all documentation pertaining to the trial for the lifetime of the product. Archived data may be held on microfiche or electronic record, provided that a back-up exists and that hard copy can be obtained if required. The protocol, documentation, approvals and all other documents related to the trial, including certificates attesting that satisfactory audit and inspection procedures have been carried out, will be kept by the Sponsor in the Trial Master File. Data on AEs are included in the Trial Master File. All data and documents will be made available if requested by relevant authorities.

12.5 Financial Contract and Insurance Coverage

An agreement will be signed by all the parties involved in the trial's performance, if relevant. Adequate insurance coverage for all subjects to be included in the trial is supplied by the Sponsor.

12.6 Stipends for Participation

The trial is set up in 4 centers.

[redacted]

Only completed visits will be taken into account.

12.7 Publication Policy

Data derived from the trial are the exclusive property of the Sponsor. Any publication or presentation related to the trial must be approved by the Sponsor before submission of the manuscript. After publication of the results of the trial, any participating center may publish or otherwise use its own data provided that any publication of data from the trial gives recognition to the trial group. In addition, the Sponsor shall be associated with all such publications, it being understood that the Sponsor is entitled to refuse the association.

The Sponsor must have the opportunity to review all proposed abstracts, manuscripts or presentations regarding this trial at least 60 days prior to submission for publication/presentation. Any information identified by the Sponsor as confidential must be deleted prior to submission, it being understood that the results of this trial are not to be considered confidential.

The Sponsor review can be expedited to meet publication guidelines.

13 Reference List

1 Laver G and Garman E. Pandemic influenza: its origin and control. Microbes Infect. 2002; 4(13): 1309-16.

2 Nguyen-Van-Tam JS and Hampson AW. The epidemiology and clinical impact of pandemic influenza. Vaccine 2003; 21 (16): 1762-68.

3 Fox JP, Hall CE, Cooney MK and Foy HM. Influenza virus infections in Seattle families, 1975-1979. I. Study design, methods and the occurrence of infections by time and age. Am J Epidemiol. 1982; 116: 212-27.

4 Fleming DM. The impact of three influenza epidemics on primary care in England and Wales. Pharmacoeconomics 1996; 9 (Suppl 3): 38-45.

5 Wood JM. Selection of influenza vaccine strains and developing pandemic vaccines. Vaccine 2002; 20: B40-B44.

6 Fedson DS. Pandemic influenza and the global vaccine supply. Clin Infect Dis. 2003; 36 (12): 1552-61.

7 Simonsen L. The global impact of influenza on morbidity and mortality. Vaccine 1999; 17: S3-S10.

8 Centers for Diseases Control and Prevention. Prevention and Control of Influenza: Recommendations of the Advisory Committee on Immunization Practices (ACIP). MMWR 2002; 51 (No. RR-3): 1-31.

9 Vu T, Farish S, Jenkins M and Kelly H. A meta-analysis of effectiveness of influenza vaccine in persons aged 65 years and over living in the community. Vaccine 2002; 20: 1831-36.

10 Nicholson KG, Colegate AE, Podda A, Stephenson I, Wood J, Ypma E et al. Safety and antigenicity of non-adjuvanted and MF59-adjuvanted influenza A/Duck/Singapore/97 (H5N3) vaccine: a randomised trial of two potential vaccines against H5N1 influenza. The Lancet. 2001; 357: 1937-43.

11 Stephenson I, Nicholson KG, Colegate A, Podda A, Wood J, Ypma E, et al Boosting immunity to influenza H5N1 with MF59-adjuvanted H5N3 A/Duck/singapore/97 vaccine in a primed human population. (2003) Vaccine 21: 1687-1693

12 Dose/Formulation Finding of Different Formulations of an Intramuscular A/H5N1 Inactivated, Split Virion Pandemic Influenza Vaccine in Adults (GPA01). Clinical Trial Protocol, Version 5.0, 04 April 05

13 Guideline of the EMEA on Dossier Structure and Content for Pandemic Influenza Vaccine Marketing Authorisation Application (CPMP/VEG/4717/03).

14 Guideline of the EMEA on Submission of Marketing Authorization Applications for Pandemic Influenza Vaccine Through the Centralised Procedure (CPMP/VEG/4986/03).

15 Ulanova M, Tarkowski A, Hahn-Zoric, M. The common vaccine adjuvant aluminum hydroxide up-regulates accessory properties of human monocytes via an interleukin-4-dependant mechanism. Infection and Immunity, 2001; 1151-59.

16 Aymard-Henry M, Coleman MT, Dowdle WR, Laver WG, Schild GC and Webster RG Influenzavirus neuraminidase and neuraminidase-inhibition test procedures. Bull. Wld. Hlth. Org. 1973, 48: 199-202

17 Newcombe R.G., Two-sided confidence intervals for the single proportion: comparison of seven methods, Statistics in Medecine, (1998) 17, 857-872.

List of Appendices

Appendix 1 List of Investigators, Trial Centers and Other Personnel Involved in the Trial
Appendix 2 Sample Informed Consent Forms (UK and Belgium Versions)
Appendix 3 Signature Page

Protocol Appendix 1: List of Investigators, Trial Centers, and Other Personnel Involved in the Trial

Protocol Appendix 1: List of Investigators, Trial Centers and Other Personnel Involved in the Trial

Coordinating Investigators	Investigator address	Center number, name and adress
[redacted] MD	Same as center	Center 2 [redacted] Belgium
[redacted] MD	Same as center	Center 3 [redacted] UK

Other investigators	Investigator address	Center number, name and adress
[redacted] MD	Same as center	Center 1 [redacted] Belgium
[redacted] MD	Same as center	Center 4 [redacted] Belgium

sanofi pasteur involved personnel	Name
Clinical Franchise Leader	[redacted] MB BS
Clinical Scientist	[redacted]
Clinical Trial Manager (CTM)	[redacted]
Statistician	[redacted]
Product Safety Officer	[redacted]

Protocol Appendix 2: Sample Informed Consent Forms (UK and Belgium Versions)

[redacted]

Bird Flu Vaccine Study

Safety and Immunogenicity of an Intramuscular, Inactivated, Split-Virion, Pandemic Influenza A/H5N1 Vaccine in Adults and the Elderly (GPA02)

Information Sheet

Dear Potential Participant,

The [redacted] is carrying out a research study of a new vaccine aimed at protecting against a pandemic influenza virus. We would like to invite you to take part in this study. This information sheet explains what the study is about and what it would involve if you decided to take part. This letter has been delivered to you by means of a general mailout to all postcodes in your area or because you have expressed an interest in the study. Please note that the [redacted] only have your name and address details if you have contacted us requesting information. [redacted] The rest of this information sheet will refer to sanofi pasteur as "the Sponsor".

This study is also being conducted simultaneously in 3 centres in Belgium.

What is Bird Flu and Pandemic Influenza?

Since December 2003, a growing number of countries have reported outbreaks of avian influenza ('bird flu') in chickens and ducks, resulting in the death or culling of more than 100 million poultry. A highly aggressive strain, A/H5N1, has been the cause of most of these outbreaks. Between December 2003 and mid-January 2006, there were 151 confirmed cases (including 82 deaths) of the A/H5N1 virus in adults and children in Vietnam, Thailand, Cambodia, China, Indonesia and Turkey.

There is concern that the bird flu (A/H5N1) virus will develop into a form that is highly infectious and for which humans would have no natural immunity (that is, the population would have no antibody in the blood to protect against the virus). Such a virus could spread easily from one person to another, resulting in pandemic influenza. Pandemic influenza means that the virus could spread over the whole world from person to person. This type of influenza is likely to be characterised by the sudden onset of severe typical influenza symptoms such as headache, high fever, muscle and joint aches, reduced appetite, nausea, vomiting and cough, which typically last for 2 to 4 days. Some individuals develop pneumonia, and although many patients recover, some may die rapidly due to difficulties with their breathing.

What treatments may be used to prevent or treat Pandemic Influenza?

It may be possible to use antiviral drugs, which are sometimes used to treat individuals infected with the influenza virus. However, vaccination is likely to remain the most effective preventative medical action against pandemic influenza.

[redacted]

Why do we want to do a study?

The aim of the study is to test two, new, influenza vaccine formulations in adults that are made from the bird flu strain, A/H5N1. We want to assess how well an adult's immune system responds to the vaccine as well as monitoring the safety of the vaccine.

A similar influenza vaccine has already been administered to 300 adults in a study conducted in France to assess various vaccine formulations with and without an adjuvant. An adjuvant is a component of many vaccines which is added to enhance the immune response. The results of this previous study showed that the highest antibody response was observed following vaccination with the 30 µg dose with adjuvant (antibodies are substances that protect the body from infections). Responses were also observed following the other doses/formulations, including 7.5 µg without adjuvant. This lower dose is of interest as it could potentially allow the administration of vaccine to a larger number of people in the event of a pandemic. All formulations were generally safe and well tolerated. Therefore, the 30 µg of vaccine with an adjuvant, and 7.5 µg of vaccine without adjuvant formulations have been chosen for further assessment in the present study.

What happens in the study?

A total of 600 adults would take part in this study in the UK (approximately 150 subjects) and Belgium (approximately 450 subjects); 300 aged 18 to 60 years and 300 aged over 60 years. Participants would be randomised into one of two groups to determine which vaccine would be given. Group 1 would receive 30 µg of vaccine with adjuvant and Group 2 would receive 7.5 µg of vaccine without adjuvant. A computer program would determine this allocation, which would be decided by chance (similar to tossing a coin). Neither you nor the study team would be able to influence which group you would be allocated to. For each vaccine formulation, 150 adults of each age group would be given two doses of vaccine (one dose at each of the first and second visits). In addition, a booster vaccination would be administered at either 6 or 12 months after the first vaccination (the timing of the booster vaccination is to be decided following a review of the data from a previous similar clinical trial).

Should you decide to volunteer for the study, you would be asked to come to [redacted] Hospital for six or seven visits over a period of 12 or 18 months. The tables below outline the two possible schedules:

For 6-month booster design:

Study Visit No:	1	2	3	4	5	6
Timescale:	Day 0	Day 21	Day 42	6 mths	6 mths + 21 days	12 mths
Informed Consent	X					
Medical History	X					
Physical Examination	X	X	X	X	X	X
Recent Health		X	X	X	X	X
Pregnancy Test (Females)	X	X		X		
Blood Sampling	X	X	X	X	X	
Vaccination	X	X		X		
Review of Diary Card		X	X	X	X	X

For 12-month booster design [redacted]

Study Visit No:	1	2	3	4	5	6	7
Timescale:	Day 0	Day 21	Day 42	6 mths	12 mths	12 mths + 21 days	18 mths
Informed Consent	X						
Medical History	X						
Physical Examination	X	X	X	X	X	X	X
Recent Health		X	X	X	X	X	X
Pregnancy Test (Females)	X	X			X		
Blood Sampling	X	X	X	X	X	X	
Vaccination	X	X			X		
Review of Diary Card		X	X	X	X	X	X

At all but the final visit a medical examination would be performed and a 30 mL (6 teaspoons) blood sample would be taken. The final 'visit' would consist of a telephone call to assess your health, with further review at the [redacted] Hospital if required. Female participants would be required to undergo a pregnancy test prior to each vaccination and would have to use an effective method of birth control for at least 4 weeks prior to and 4 weeks after each vaccination. At the appropriate visits the vaccine would be administered into the upper arm. To ensure your safety, you would stay at the study centre under medical observation for 30 minutes following vaccination. We would ask you to keep a diary of any medical events or symptoms that may appear after vaccination until the following visit, as well as any medication taken during that time. We would provide you with a thermometer and ask you to measure your oral temperature in the evening for the 7 days after each vaccination and at any time that you feel feverish. A 24 hour telephone number would be provided to enable you to contact one of our doctors should you have any concerns. You would be asked to contact us if, for any reason, you attend hospital during the study period.

What are the possible side effects of the vaccines and of blood sampling?
Influenza vaccines are usually well tolerated and this vaccine is made in a similar way to the vaccines that are given to millions of individuals around the world every year. As with the administration of any other vaccine, you may experience a variety of unwanted reactions at the injection site in the days after vaccination including: pain, redness, hardness, swelling or bruising. You may also feel unwell or experience headache, muscle and joint pain, high temperature or malaise. These effects usually disappear without treatment within 3 days. Following immunisation with influenza vaccines, between 1 in 100 and 1 in 1000 of people experience a more widespread rash. Nerve pain, paraesthesia (a feeling of 'pins and needles'), convulsions and temporary problems with blood clotting have occurred rarely (i.e., between 1 in 1000 and 1 in 10,000 of those receiving the vaccine). Very rare (less than 1 in 10,000) reactions like neurological disorders have been reported. This is not a complete list of possible effects. There is no reason to think that these conditions will be any more likely with this new vaccine. It is very important that you let the study doctor or nurse know about any effects you experience.

As with any vaccination, there is a rare possibility of an allergic reaction. This may cause a severe narrowing of the air passages and breathing difficulties. You would be observed for 30 minutes following each vaccination to monitor for allergic reactions; staff are specifically equipped to deal with such rare events. In addition, there may occasionally be some slight bruising following the blood sampling.

Influenza vaccines have been given safely in pregnancy but we do not intend to give this new vaccine to anyone who is pregnant as we do not yet know whether there could be any risks to the baby. If you were to become pregnant

[redacted]
during the study you would be withdrawn but would be followed until the end of your pregnancy to monitor the health of you and your baby.

What will happen to your blood samples?
As a part of this study, blood samples would be taken to test the effect of the vaccine on your body's immune system. These tests would be done by the Sponsor or by another laboratory for the Sponsor. Your samples would be shipped out of the United Kingdom for these tests and would be stored in a freezer until these tests are done. Therefore, you should only agree to take part in this study if you agree to this use of your blood samples. They would be stored until at least 10 years after the vaccine being tested has been approved for use by the health authorities.

What should you know about possible future use of your samples for research?
The Sponsor would also like to keep any unused part of your blood samples for use in other research in the future. The aim of any future research is unknown today, and may not be related to this particular study. It may be to either improve our knowledge of vaccines or infectious diseases, or to improve laboratory methods. When these samples came to be used in future studies your identifying details would be removed. You would not be informed about the types of research that would be conducted or any of the results, although genetic tests would never be performed on these samples. You can agree to participate in the study and still refuse the storage of any unused blood samples for future use by the Sponsor. You would not be reimbursed for allowing the Sponsor to keep and later use any remaining part of your blood samples.

What are the possible benefits to me?
We do not yet know if this vaccine will protect against the bird flu strain that has caused the infections in humans described at the beginning of this information sheet. If the bird flu virus does develop into a pandemic virus that transmits readily from person to person, we do not know if it will be close enough to the strain used in this vaccine for you to receive any benefit. No benefit can be guaranteed.

Who will see my medical and personal information?
If you decide to take part, your study records may be read by representatives of the Sponsor, the [redacted] and other regulatory authorities to check that the information collected is correct and that the study is being carried out correctly. Should it be necessary (for example, if you were admitted to hospital) we would ask your permission to access your medical records. All information that is collected about you would be kept strictly confidential. Any information about you that leaves the study centre would have your name and address removed so that you cannot be identified from it and you may exercise your right to access and correct your data. This anonymised information would be computerised and sent to the Sponsor, and, if necessary, to authorised regulatory authorities. Your name would not appear in any publication or report that may be produced from this study.

What happens if I say no?
Taking part in research is voluntary. If you decide to say ‘no’ this will not affect your routine care in any way. You are also free to change your mind at any point during the study duration without giving any reason. If you withdraw from the study, the blood samples collected before your withdrawal would be used unless you specifically request otherwise.

What if I wish to complain about the way in which this study has been conducted?
If you have any cause to complain about any aspect of the way in which you have been approached or treated during the course of this study, the normal National Health Service complaints mechanisms are available to you and are not

[redacted] compromised in any way because you have taken part in a research study. [redacted]

What else do you need to know before agreeing to take part in this study?

This study has the approval of the [redacted] Committee. In the event that you suffer any harm during the study you are fully indemnified. As with all studies of this kind, insurance for participants in the study has been arranged according to the guidelines issued by the Association of the British Pharmaceutical Industry (1991). [redacted]

You would be informed as soon as possible should any information become available during the course of the study that may affect your continued participation. The study medical team may withdraw you from the study at anytime, for example if the Investigator decides that it is in your best interest or you are unable to attend study appointments.

If you withdraw, or are withdrawn, from the study the Investigator may recommend that certain laboratory tests or physical examinations should be done to ensure your safety and well-being. You would be requested not to have any vaccinations in the 4 weeks before or after receiving the study vaccine. Taking part in this study would not remove the need for the routine influenza vaccine in individuals for whom it is indicated. With your permission we would inform your GP that you are taking part. We would reimburse you for your time and travel expenses to a total of [redacted] per study visit.

So, in summary, what would happen if I decide to take part in the study?

- You would receive 3 doses of a pandemic influenza virus vaccine over 6 or 12 months
- Over the study period you would have 5 or 6 samples of blood collected
- All study visits would take place at the [redacted] Hospital
- You would be asked to complete a diary card after each visit
- You would have 24-hour telephone access to a study doctor in case you have any concerns following immunisation
- You would be enrolled in the study for a period of 12 or 18 months
- You would receive a copy of the signed Informed Consent Form

What do I do now?

Thank you for considering taking part in this study. You do not need to make a final decision straight away. Please fill in the attached form indicating your preference and return it in the pre-paid envelope enclosed. [redacted]

Yours sincerely

[redacted]

[redacted]

[redacted]

Bird Flu Vaccine Study

Safety and Immunogenicity of an Intramuscular, Inactivated, Split-Virion, Pandemic Influenza A/H5N1 Vaccine in Adults and the Elderly (GPA02).

Consent Form

First and last name: ____

Initials: ___ ___ Subject Number: [redacted] ___ ___ ___ ___ ___ *Initial each box if you agree*

I have read the information letter *(Information/Reply Sheet Version 2 dated 04 April 06)* ☐

I have had the opportunity to ask questions and discuss the study, and understand that I may ask additional questions at any time ☐

I have received satisfactory answers to my questions ☐

I have received enough information about the study ☐

Who have you spoken to? (print name) ……………………………………………………………

I understand that I am free to withdraw from the study, at any time, without having to give a reason, and without it affecting my routine medical care ☐

I agree to my GP being informed of my participation in the study ☐

I agree to my medical records being accessed if it is deemed necessary ☐

I understand that information collected about me during the study will be computerised and may be looked at by individuals from the sponsor or regulatory authorities ☐

I am aware that I may exercise my right of access and correction of these data at any time with the Investigator ☐

I understand that if I withdraw my consent, blood samples collected before my withdrawal will be used unless I specifically request otherwise ☐

I authorise the shipment of my blood samples to the Sponsor's laboratory (or other location as applicable) for doing tests related to this study, and I understand that the laboratory may be in another country ☐

The next question is for females participants only:

I agree to have a routine pregnancy test prior to each vaccination and to use birth control methods as outlined in the 'What happens in the study?' section of the information sheet ☐

[redacted]

If all of the applicable questions above are initialled then please continue

I agree to participate in this study ☐

Please note: you can still participate in this study whether or not you agree to the next statement

I agree that any remaining blood may be stored and used in future research by the Sponsor as outlined in the section 'What should I know about possible future use of my blood samples for research?'

Yes: ☐ No: ☐

**Volunteer's name:** .... … <br>**Signature:** **Date: |___ ___| |___ ___| |___ ___|**
**Investigator/Study nurse's name** (*please delete as appropriate*)**:** …. .... <br>**Signature:** **Date: |___ ___| |___ ___| |___ ___|**

[redacted]

[redacted]

Bird Flu Vaccine Study

Safety and Immunogenicity of an Intramuscular, Inactivated, Split-Virion, Pandemic Influenza A/H5N1 Vaccine in Adults and the Elderly (GPA02)

Addendum #1 to Information Sheet

Dear Participant,

At the beginning of the study you were informed that there would be a booster vaccination in the schedule but that the timing of this had not been decided. This letter is to inform you that the booster dose will now be at 12 months. We would also like to inform you of some changes that have been made to the study design by the Sponsor. The changes are as follows:

- The booster will be based on a new strain of the H5N1 (bird flu) virus; this is slightly different from that used in the vaccine you have already received.
- The dose of the booster doses remains as 30µg + adjuvant or 7.5µg without adjuvant. At the 12 month visit you will be randomised into one of the 2 vaccine groups; this means that you may or may not receive the same dose of the vaccine as you received previously.
- At one site in Belgium participants will receive the booster vaccination at 6 months. The vaccine used in this group will be the same H5N1 vaccine as that used in the primary phase.
- An additional 100 participants will be recruited into the study at a site in Belgium in May 2007. These participants will receive the two initial doses (but no booster dose) of the vaccine based on the new H5N1 strain.

The changes to the study design have been made in response to a change in the circulating H5N1 virus. Except for the strain, this new vaccine is made in the same way as the vaccine you received in the first part of the study. Variations in the H5N1 virus have always been expected and mean that we may need to be prepared to immunise against more than one strain in a pandemic.

As was outlined in the original information sheet we do not yet know if the vaccines used in this study will protect against the bird flu strains that have caused infections in humans. Furthermore, if the bird flu virus does develop into a pandemic virus we do not know if it will be close enough to the strains used in these vaccines for you to receive any benefit or cross protection. Although no protection to you can be guaranteed the results of the study will help us to be better prepared to produce a suitable vaccine in the event of a pandemic.

[redacted]

[redacted]

At the 6 month visit, when a blood sample will be obtained but no vaccine administered, we will discuss these changes with you and answer any questions you may have. You will be asked to complete the section at the end of this letter to confirm that you are happy to participate in the booster part of this study. However, as always, participation is voluntary.

As the booster vaccination is now scheduled for May/June 2007 there is no issue concerning the timing of the winter flu vaccine and the study vaccine. For those of you who are due to receive the winter flu vaccine, please do so as advised by your General Practitioner.

We would be very grateful if, over the coming months, you could inform us of any changes to your details so that we can remain in contact. [redacted]

Yours sincerely

[redacted] [redacted] [redacted]

[redacted]

**Bird Flu Vaccine Study**<br><br>Safety and Immunogenicity of an Intramuscular, Inactivated, Split-Virion, Pandemic Influenza A/H5N1 Vaccine in Adults and the Elderly (GPA02).

Addendum #1 to Information Sheet

First and last name: ____

Initials: ___ ___ Subject Number: [redacted] ___ ___ ___ ___ ___ *Initial each box if you agree*

I have read this addendum to the Information Sheet and I certify that I voluntarily accept to continue participating in the study and to receive the booster vaccination at 12 months after the first vaccination as described in this addendum.

**Volunteer's name:** ....<br><br>**Signature:** **Date: |___ ___| |___ ___| |___ ___|**
**Investigator/Study nurse's name** (*please delete as appropriate*)**:** ....<br><br>**Signature:** **Date: |___ ___| |___ ___| |___ ___|**

[redacted]

Bird Flu Vaccine Study

Safety and Immunogenicity of an Intramuscular, Inactivated, Split-Virion, Pandemic Influenza A/H5N1 Vaccine in Adults and the Elderly (GPA02)

Addendum #2 to Information Sheet

Dear Participant,

At your last visit we informed you that the booster vaccination would be performed 12 months after your first vaccination. The booster vaccine was to be based on a new strain of the H5N1 (bird flu) virus that is slightly different from that used in the vaccine you have already received.

Unfortunately, due to difficulties in production, the booster vaccine will not be ready in time to carry out the next phase as planned. This unplanned delay in the booster vaccination, has meant that the study design has had to be modified. While it is still intended to administer the same booster vaccination as described in Addendum #1 to the Information Sheet we are currently unable to let you know when this will be, but will inform you once this date is known. In the meantime we would like to invite you for an additional visit in September 2007 for a 30 ml (6 teaspoons) blood test but no vaccination. This blood test will give us important information on the persistence of responses to the vaccine. As for the other visits, you will be re-imbursed for your time and travel expenses to a total of [redacted] per study visit.

At your next visit, we will discuss these changes with you and answer any questions you may have. You will be asked to complete the section at the end of this letter to confirm that you agree to participate in the additional visit and are happy to continue to participate in the booster part of this study. However, as always, participation is voluntary.

As was outlined in the original information sheet we do not yet know if the vaccines used in this study will protect against the bird flu strains that have caused infections in humans. Furthermore, if the bird flu virus does develop into a pandemic virus we do not know if it will be close enough to the strains used in these vaccines for you to receive any benefit or cross protection. Although no protection to you can be guaranteed the results of the study will help us to be better prepared to produce a suitable vaccine in the event of a pandemic.

[redacted]

We would be very grateful if, over the coming months, you could inform us of any changes to your details so that we can remain in contact. [redacted]

Yours sincerely

[redacted] [redacted] [redacted]

Addendum #2 to Information Sheet

First and last name: ____

Initials: ___ _ Subject Number: [redacted] ___ ___ ___ ___

I have read this addendum to the Information Sheet and I certify that I voluntarily: *Please initial to indicate 'yes'*

agree to participate in the additional visit according to the changes described in this addendum ☐

agree to continue participating in the study according to the changes described in this addendum. ☐

Please note: you can still participate in this study whether or not you agree to the next statement

I agree that any remaining blood may be stored and used in future research by the Sponsor as outlined in the section 'What should I know about possible future use of my blood samples for research?' of the original Information Sheet

Yes: ☐ No: ☐

Volunteer's name: ....

Signature: **Date:** |___ ___| |___ ___| |___ ___|

Investigator/Study nurse's name (*please delete as appropriate*)**:** ....

Signature: **Date:** |___ ___| |___ ___| |___ ___|

[redacted]

Bird Flu Vaccine Study

Safety and Immunogenicity of an Intramuscular, Inactivated, Split-Virion, Pandemic Influenza A/H5N1 Vaccine in Adults and the Elderly (GPA02)

Addendum #3 to Information Sheet for participants who have received the 30 µg vaccine with adjuvant for the first and second vaccinations

Dear Participant,

A booster phase has been planned from the start of the study, however several changes have been made since that time. At your last appointment we informed you that the booster vaccine was to be made from a slightly different strain of the H5N1 virus. This booster dose was to be either a 30 µg with adjuvant or 7.5 µg without adjuvant. At that time we were unsure of the timing of the booster phase due to problems with the production of the new vaccine.

We can now confirm that the booster vaccination phase will be in March 2008, 22 months from the start of the study. Although the booster vaccination will still be based on a slightly different strain of the virus, only the 30 µg dose with adjuvant and not the 7.5 µg dose without adjuvant will be used. The 7.5 µg vaccine without adjuvant is no longer being used as poor immune responses were found when it was given as a booster dose to a group of participants in Belgium. Furthermore no participants who received the 7.5 µg vaccine without adjuvant will complete the booster phase of the study. The final change to the study is an additional appointment for a blood test 7 days after the booster vaccine as detailed below.

Study Visit No:	5	5_add	6	Follow-up phone call
Timescale:	Month 22	Month 22 + 7 days	Month 22 + 21 days	28 months
Informed Consent	X			
Physical Examination	X	X	X	
Recent Health		X	X	X
Pregnancy Test (Females)	X			
Blood Sampling	X	X	X	
Vaccination	X			
Review of Diary Card		X	X	
Review of Memory Card	X			X

[redacted]

In total 3 blood tests of 30 ml (6 teaspoons) would be taken: one just before the vaccine, one at 7 and one 21 days after the vaccine. The extra blood test at 7 days will give us information on how quickly you respond to the vaccine; this is a measure of the influence, if any, of the vaccines given to you in the first part of the study. As after the previous vaccinations we would ask you to wait for 30 minutes as a precaution and to also fill in a diary card of any symptoms you have in the 21 days after vaccination. Once again, female participants would be required to undergo a pregnancy test prior to vaccination and would have to use an effective method of birth control for at least 4 weeks prior to and 4 weeks after vaccination. A re-imbursement of [redacted] per appointment will be made at the end of the study.

During your next appointment we will discuss the changes with you in detail and answer any questions you may have. If you are happy to go ahead we will ask you to complete a consent form as shown at the end of this letter (there is no need to complete this until your appointment). As always your continued participation is voluntary.

The results of the study will help us to be better prepared to produce a suitable vaccine in the event of a pandemic. No protection from the study vaccines can be guaranteed as we do not know which bird flu strains if any will cause a pandemic.

There is no need for you to do anything now ase will be contacting you over the next month to arrange your next appointments. [redacted]

Thank you again for you participation in the study so far. We apologise for any inconvenience the changes to the study may have caused.

Yours sincerely

[redacted]

[redacted]

[redacted]

[redacted]

[redacted]

[redacted]

[redacted]

Bird Flu Vaccine Study

Safety and Immunogenicity of an Intramuscular, Inactivated, Split-Virion, Pandemic Influenza A/H5N1 Vaccine in Adults and the Elderly (GPA02).

Addendum #3 to Information Sheet

First and last name: ____

Initials: ___ ___ __ Subject Number: [redacted] ___ ___ ___ ___ ___ *Initial each box if you agree*

I have read this addendum to the Information Sheet and I certify that I voluntarily: *Please initial to indicate 'yes'*

- agree to continue participating in the study according to the changes described in this addendum. ☐

Please note: you can still participate in this study whether or not you agree to the next statement

I agree that any remaining blood may be stored and used in future research by the Sponsor as outlined in the section 'What should I know about possible future use of my blood samples for research?' of the original Information Sheet

Yes: ☐ No: ☐

Volunteer's name: ....

Signature: **Date:** |___ ___| |___ ___| |___ ___|

Investigator/Study nurse's name (*please delete as appropriate*): ....

Signature: **Date:** |___ ___| |___ ___| |___ ___|

INFORMED CONSENT FORM

Safety and Immunogenicity of an Intramuscular, Inactivated, Split-Virion, Pandemic Influenza A/H5N1 Vaccine in Adults and the Elderly (GPA02).

Name and Address of Investigator:	[redacted] Belgium (Center 1).
	[redacted] Belgium. (Center 4).
Name and Address of Sponsor:	sanofi pasteur<br>2, avenue Pont Pasteur<br>F-69367 Lyon cedex 07<br>France

You are being asked to volunteer to take part in a research study to help to develop a vaccine against a pandemic influenza virus, which is being conducted in the UK and Belgium. Although this vaccine has been approved for use in this particular study, it has not been licensed for use in the UK or Belgium by the Medicines and Health-Care Products Regulatory Agency (MHRA) or the Directorate-General for Public Health Protection – Medicinal Products (DGMP), respectively. No guarantee or assurance can be made regarding the results of this study.

This study is being paid for by sanofi pasteur (the rest of this form will refer to sanofi pasteur as "the Sponsor"). Dr. ………………….. is the "investigator" for the study, and his/her institution will receive a research grant for the conduct of this study. The Ethics Committees (EC)/Institutional Review Boards (IRB) of your center approved this study.

This document will provide you with the information needed to help you decide whether you wish to take part in this study. If any part or word of this document is unclear, or if you have any questions or want additional information at any time, please do not hesitate to ask one of the study team members.

What is Pandemic Influenza?

Since December 2003, a growing number of Asian and Eastern European countries have reported outbreaks of highly pathogenic avian influenza in chickens and ducks, resulting in the death or culling of more than 100 million poultry. A highly pathogenic strain, A/H5N1, has been the cause of most of these outbreaks.

Between December 2003 and mid-January 2006, A/H5N1 viruses have caused at least 151 confirmed cases (including 82 deaths) in human adults and children in Vietnam, Thailand, Cambodia, China, Indonesia and Turkey. To date, people who have been infected with the A/H5N1 virus have been in direct contact with infected and sick birds. The virus has not been infectious between humans.

There is a concern that the A/H5N1 virus, if given sufficient opportunity, will mutate into a form that is highly infectious and for which humans would have no natural immunity (that is, the population would have no antibody in the blood to protect against the virus). Such a virus could spread easily from one person to another, resulting in pandemic influenza.

Past influenza pandemics have been characterized by the sudden onset of severe typical influenza symptoms such as headache, high fever, muscle and joint aches, reduced appetite, nausea, vomiting and cough, which typically last for 2 to 4 days. Some patients, after an initial recovery, subsequently develop pneumonia, and although most patients ultimately recover, some die rapidly due to tracheo-bronchitis associated with breathing difficulties.

What vaccines or other treatments may be used to prevent or treat Pandemic Influenza?

In the event of a pandemic, it will be possible to use antiviral drugs, which are commonly used to treat influenza each year (for more information, ask your doctor), to control pandemic influenza and to treat those infected with the virus however the effectiveness of anti-viral drugs in the treatment of pandemic influenza has yet to be demonstrated. Vaccination may be effective at preventing pandemic influenza but as with antiviral drugs the use of an A/H5N1 vaccine in an A/H5N1 pandemic has to date not been demonstrated.

What is the purpose of this study?

This is a medical research study, the purpose of which is to test two influenza pandemic vaccine formulations as a two-primary dose schedule plus a booster vaccination in A/H5N1-naïve subjects (that is, subjects who have not previously been exposed to the virus contained in the vaccine) aged either 18 to 60 years, or >60 years. The influenza pandemic vaccine is produced from an avian influenza strain, specifically an A/H5N1 virus ("H" and "N" stand for two proteins present on the surface of the virus and these two proteins are responsible for the virulence of the virus).

A similar influenza vaccine has already been injected in 300 naïve adults (aged 18 to 40 years) in a study conducted in France. This study tested several adjuvanted and non-adjuvanted A/H5N1 vaccine formulations (from 7.5 µg to30 µg of vaccine given as a two-dose schedule. An adjuvant is a component of many vaccines, which is added to enhance the immune response. Each formulation of the vaccine appeared to be generally safe and well tolerated. The highest antibody (substances that protect the body from infections) response was observed following vaccination with the 30 µg dose with adjuvant, and responses were also observed following the other doses/formulations, especially following 7.5 µg without adjuvant. This study is ongoing, with an evaluation of the amount of antibodies (the substances that protect the body from viral infections) in the blood to be completed at 6 and 12 months following the first vaccination.

Based on these results, the 30 µg of vaccine with adjuvant and 7.5 µg of vaccine without adjuvant formulations have been selected for use in the present trial. The objectives of the present study are to describe the safety and the immune response following administration of two vaccinations (separated by 21 days) followed by a booster vaccination. The booster dose will be administered 6

or 12 months after the first vaccination. A final decision regarding the time of administration of the booster (6 or 12 months) and the formulation (30 µg of vaccine with adjuvant and 7.5 µg of vaccine without adjuvant) will be made following a review of further data from the previous study.

How will this study be done?

This study will consist of six (design with booster at 6 months) or seven (design with booster at 12 months) visits (although the final 'visit' may be conducted by telephone) and will last 12 or 18 months, respectively. It will involve 300 adults aged 18 to 60 years and 300 adults aged >60 years, with 150 adults in each age group receiving either one or the other of the two vaccine formulations. The trial will be performed in the UK and Belgium.

At the start of the trial, you will be assigned by chance to receive one of the two selected formulations (30 µg of vaccine with adjuvant or 7.5 µg of vaccine without adjuvant) and will receive two injections of the same vaccine formulation, separated by an interval of 21 days. In addition, a third (booster) vaccination will be performed 6 or 12 months after the first vaccination.

Should you decide to volunteer for the study, you will be asked to come to the study center for six or seven visits, according to the schedule that will be decided following a review of the data from the previous study, and to record any unwanted medical effects that could occur between these visits. At each visit, the Investigator will examine you and will ask you questions about your health. In addition, a blood sample (30 mL) will be taken from each subject at each visit to measure the amount of antibodies.

If you are a participant in Center 4 ([redacted] Belgium), you will have additional blood samples (20 mL extra blood per sample) to be taken at Visits 1, 3, 4 and 5 or Visits 1, 3, 5 and 6 for further measurements of the immune response.

Once all the results of the previous study are available, the decision to administer a booster vaccination at either 6 or 12 months after the first vaccination, and the formulation to be used, will be made. Both options for a booster at 6 months or 12 months are presented below and you will be informed of the decision once this information is available.

- First visit:

All the risks and benefits associated with the study will be explained to you before signing the following informed consent statement. After having received satisfactory answers to all your questions, and if you agree to take part in the study, you will sign the informed consent form.

The Investigator will examine you and check your medical history to make sure that you can take part in the study. This involves a physical examination, a urine pregnancy test if you are a woman of child-bearing potential, and recording of your medical history (especially regarding any allergic reactions you may have had to previous vaccinations or any allergy to egg proteins). You will be asked not to participate in another research study for the whole period of the present study.

If you are eligible for the study, 30 mL of blood will be taken in order to measure your level of antibodies before vaccination. In addition, an extra 20 mL will be taken only from subjects enrolled at Center 4 for further immunogenicity analyses. You will then receive an injection of one of the two vaccine formulations into your upper-arm muscle. To ensure your safety, you will stay at the study center under medical observation for 30 minutes following vaccination.

You will then be given a digital thermometer, a ruler, and a diary card to note any unwanted effects that may happen between vaccination and the next visit, and you will be instructed on how to use them. On the evening of vaccination and on each of the next 7 days, you will be asked to record your oral temperature and any specified symptoms commonly observed after any vaccine administration. You will also be asked to record any medications (other than the vaccine) that you may take during the 7 days following the vaccination. In addition, any non-specified symptoms occurring between the vaccination and the second visit should be recorded in the diary card.

- Second visit: 21 days after the first vaccination:

The Investigator will examine you, review with you the diary card that you will have completed since the first vaccination and will question you on your health since vaccination. A urine pregnancy test will be performed if you are a woman of child-bearing potential. A 30 mL blood sample will be taken to test the effect of the vaccine on your body's immune system. Then you will receive the second vaccine dose into your upper-arm muscle. To ensure your safety, you will stay at the study center under medical observation for 30 minutes following vaccination.

You will then be given a second diary card to note any medications that you may take and unwanted effects that may happen between the second vaccination and the next visit.

- Third visit: 21 days after the second vaccination:

The Investigator will examine you, review with you the diary card that you will have completed since the previous visit and will question you on your health since the previous visit. A 30 mL blood sample (and an extra 20 mL for those subjects enrolled in Center 4) will be taken in order to further investigate the effect of the vaccine on your body's immune system.

A memory aid will be provided to help you to record any important events until the next visit.

Trial design with a 6-month booster design: fourth visit to sixth visit:

- Fourth visit: 6 months after the first vaccination:

The Investigator will examine you, review with you the memory aid that you will have completed since the previous visit and will question you on your health since the previous visit. A urine pregnancy test will be performed if you are a woman of child-bearing potential. A 30 mL blood sample (and an extra 20 mL for those subjects enrolled in Center 4) will be taken in order to further investigate the effect of the vaccine on your body's immune system. Then you will receive the third vaccine dose (booster vaccination) into your upper-arm muscle. To ensure your safety, you will stay at the study center under medical observation for 30 minutes following vaccination.

You will then be given a third diary card to note any medications that you may take and unwanted effects that may happen between the booster vaccination and the next visit.

- Fifth visit: 21 days after the booster vaccination:

The Investigator will examine you, review with you the diary card that you will have completed since the previous visit and will question you on your health since the previous visit. A 30 mL blood sample (and an extra 20 mL for those subjects enrolled in Center 4) will be taken in order to further investigate the effect of the vaccine on your body's immune system.

A second memory aid will be provided to help you to record any important events until the next visit.

- Sixth visit (or telephone call): 12 months after the first vaccination:

The Investigator will examine you (or interview you over the telephone), review with you the memory aid that you will have completed since the previous visit and will question you on your health since the previous visit. This visit will mark the end of your participation in the study.

Trial design with a 12-month booster design: fourth visit to seventh visit:

- Fourth visit: 6 months after the first vaccination:

The Investigator will examine you, review with you the memory aid that you will have completed since the previous visit and will question you on your health since the previous visit. A 30 mL blood sample will be taken in order to further investigate the effect of the vaccine on your body's immune system.

A second memory aid will be provided to help you to record any important events until the next visit.

- Fifth visit: 12 months after the first vaccination:

The Investigator will examine you, review with you the memory aid that you will have completed since the previous visit and will question you on your health since the previous visit. A urine pregnancy test will be performed if you are a woman of child-bearing potential. A 30 mL blood sample (and an extra 20 mL for those subjects enrolled in Center 4) will be taken in order to further investigate the effect of the vaccine on your body's immune system. Then you will receive the third vaccine dose (booster vaccination) into your upper-arm muscle. To ensure your safety, you will stay at the study center under medical observation for 30 minutes following vaccination.

You will then be given a third diary card to note any medications that you may take and unwanted effects that may happen between the booster vaccination and the next visit.

- Sixth visit: 21 days after the booster vaccination:

The Investigator will examine you, review with you the diary card that you will have completed since the previous visit and will question you on your health since the previous visit. A 30 mL blood sample (and an extra 20 mL for those subjects enrolled in Center 4) will be taken in order to further investigate the effect of the vaccine on your body's immune system.

A third memory aid will be provided to help you to record any important events until the next visit.

- Seventh visit (or telephone call): 18 months after the first vaccination:

The Investigator will examine you (or interview you over the telephone), review with you the memory aid that you will have completed since the previous visit and will question you on your health since the previous visit.

This visit will mark the end of your participation in the study.

What are the risks and possible unwanted effects of vaccination and blood sampling?

Influenza vaccines are usually well tolerated when injected into a muscle. However, as with the administration of any other vaccine, you may experience a variety of unwanted events at the injection site, such as pain, redness, hardness, swelling, or bruising, which usually occur within

3 days after vaccination and resolve spontaneously within 3 days. You may also feel unwell or experience headache, muscle and joint pain, high temperature (39°C to 40°C), or malaise. These effects usually disappear without treatment within 3 days. This is not a complete list of possible effects.

Since influenza vaccines have been marketed, a few other reactions like itching, urticaria, or redness on the entire body have been observed infrequently (i.e., in less than 1 out of 100 persons and more than 1 out of 1,000). Nerve pain, paresthesia (feeling of "pins and needles"), convulsions, and temporary low blood platelet count have occurred rarely (i.e., in less than 1 out of 1,000 persons and more than 1 out 10,000). Very rare reactions like neurological disorders have been reported (in less than 1 out of 10,000 persons). As with any vaccination, there is a rare possibility of an allergic reaction. This may cause a severe narrowing of the air passages and breathing difficulties. If this occurs, you will need immediate medical attention. This is why you are requested to remain under medical observation in the study center for 30 minutes after each vaccination.

In addition, the sampling of blood from a vein may cause some pain, bruising at the puncture site, or lightheadedness.

There may be also other risks for you or, if you are a woman, for your unborn child, should you become pregnant during the study, that are not yet known. If you are pregnant at the moment, inform the Investigator. If you become pregnant between the first and the booster visit, the Investigator will withdraw you from the study but will follow you until the end of your pregnancy to monitor your health and that of your baby.

What are the possible benefits for you?

The efficacy of the vaccine has not yet been established and there is no certainty that the influenza strain used in this vaccine will induce a pandemic. As such, no benefit can be guaranteed.

What will you have to do?

If you agree to take part in this study, you will have to attend the six (design with booster at 6 months) or seven (design with booster at 12 months) planned visits. As described above, after each vaccination, you will be given a diary card to note any medical events or symptoms that may appear after vaccination until the following visit, as well as any medication taken during that time. You will also have to measure your temperature orally in the evening following each vaccination and for the seven subsequent days, and each time you feel feverish.

You or a family member must inform the Investigator as soon as possible if a serious medical event occurs, e.g., if you are admitted to hospital or visit an emergency department.

Women will have to use an effective method of birth control for at least 4 weeks prior to and 4 weeks after each vaccination (including the booster vaccination at 6 or 12 months). You will have to inform the investigator as soon as possible should you become pregnant. If you plan to become pregnant during the next 12 months or 18 months, you should not participate in the study.

Who will see your medical and personal information?

Personal information collected during the study will be confidential (with the exception of the situation described in the paragraph below); this confidential information will be computerized and sent to the Sponsor, and, if necessary, to authorized regulatory authorities. The personal

information that will be collected includes medical data, date of birth, and age. You may exercise your right to access and correct your data. In these files, your name will be coded using your initials and a study number. This is to protect your privacy. Your name and other information identifying you will not be forwarded to the Sponsor.

In addition to the use of computerized information as described above, any personal records, including your medical records and health history, relating to this study may be inspected in the Investigator's offices by authorized representatives of the Sponsor, or the health authorities in any country where the study vaccine may be considered for approval. Absolute confidentiality of your records can therefore not be guaranteed.

The results of this study may be presented at meetings or in publications. Your identity will not be revealed in these presentations.

What happens if you refuse to take part in the study or change your mind after you agree?

Your participation in this study is entirely voluntary. If you do not want to take part then you do not have to. Also, if you agree to take part but change your mind, you have the right to do so. You can withdraw from the study at any time without any liability for you. If you withdraw from the study, the blood samples collected before your withdrawal will be used unless if you specifically request otherwise.

Whatever your decision, you will continue to qualify for full medical care without any penalty or loss of benefits to which you are otherwise entitled.

The Investigator or Sponsor may withdraw you from this study for any reason at any time even without your consent, for example if the Investigator decides that it is in your best interest, or if you do not follow the study instructions.

If you do decide to withdraw from the study, or if you are withdrawn by the Investigator, then the Investigator may recommend that certain laboratory tests or physical examinations should be done to ensure your safety and well-being.

What else do you need to know before agreeing to take part in this study?

Taking part in this research is entirely voluntary. You will be provided with any new relevant information during the study that may affect your willingness to continue taking part.

The investigator will inform your general practitioner about your participation in this study unless you do not consent to this.

This study has been designed and will be carried out in accordance with the ICH/GCP guidelines laid down in the Declaration of Helsinki for the protection of individuals participating in clinical trials.

You will receive a copy of this signed Informed Consent Form. Please keep it safe and use it for information and reference throughout the trial.

In accordance with the law of 07 May 2004 regarding experiments involving human subjects, the Sponsor, even if faultless, is liable for any damages suffered by the participants which are directly or indirectly related to the study. Insurance has been taken out for this purpose.

You will be given a stipend for participation of [redacted] per trial visit.

Do not sign this Informed Consent Form or participate in this study unless you are comfortable with the risks involved and you have had an opportunity to ask questions and feel you have received satisfactory answers.

What will happen to your blood samples?

As a part of this study, blood samples will be taken to test the effect of the A/H5N1 vaccine on your body's immune system. These tests will be done by the Sponsor or by another laboratory for the Sponsor. Your samples will be shipped out of your country for these tests and will be stored in a freezer until these tests are done. Therefore, you should only agree to take part in this study if you agree to this use of your blood samples.

Any unused part of your blood samples will be securely stored by the Sponsor in the USA. They will be stored until at least 10 years after the vaccine being tested has been approved for use by the health authorities.

What should you know about possible future use of your samples for research?

The Sponsor would also like to keep any unused part of your blood samples for use in other evaluation studies, e.g. studies of the immune response to influenza vaccines, improvement of knowledge and documentation of the safety of this vaccine by using new developed laboratory methods or applying newly discovered concepts. You will not be informed about the types of research that will be conducted or any of the results, although, genetic tests will never be performed on these samples.

You have the right to agree to take part in the study proposed to you today and still refuse to allow the Sponsor keep any unused blood samples for future use. You will be asked to decide whether or not you permit this future use of your blood samples and to indicate your decision on the consent form.

You will not be paid for allowing the Sponsor to keep and later use any remaining part of your blood samples.

Should you refuse to allow the Sponsor to keep your unused blood samples then they will not be used for any testing other than that directly related to this study.

Who can you contact if you have questions?

If you have any questions about this study or if you want more information on compensation or medical treatment in the case of study injuries, please contact:

Name: ……………….

Tel. Number: …………………..

If you have any questions about your rights as a subject in this study, please contact:

Name: ……………….

Tel. Number: …………………..

Informed consent statement for participating in the study

Subject's Last and First Names: ____

Subject’s Study Number: ______________ Subject’s Initials: _________

By signing this form I certify to all of the following:

- I have read this entire Informed Consent Form (or had the information read to me) and understand what will be done to me and what I am being asked to do.
- I consent to take part in this study.
- I consent to make confidential personal information available for review (direct access) to the Sponsor's representative or to any Competent Authorities, Institutions or governmental agencies assigned this task in this country or in another country where the study vaccine may be considered for approval, or, if applicable, the IRB Ethics Committee.
- I understand and accept that information related to me, including information on my ethnic origin, collected during the study will be coded so that my name does not appear in my record, and that this information is computerized and shared with the Sponsor. I am aware that my computerized information may be handled in another country. I am aware that I may exercise my right of access and correction of these data at any time with the investigator.
- I have had the opportunity to ask questions and I understand that I will receive a signed copy of this Informed Consent Form.
- I understand that I may ask additional questions about this study at any time.
- I understand that I am free to withdraw from the study at any time without justifying my decision to do so and without it affecting my medical care.
- I understand that if I withdraw my consent, blood samples collected before my withdrawal will be used unless I specifically request otherwise.
- I authorize the shipment of my blood samples to the Sponsor’s laboratory (or other location as applicable) for doing tests related to this study, and I understand that the laboratory may be in another country.

Specific consent statement for the future use of your blood samples in research:

Please indicate your choice by checking (ticking) one of the two boxes below:

☐ I authorize the future use of my blood samples for future research by the Sponsor under the conditions described in this information sheet (see the paragraph called "*What should you know about possible future use of you blood samples for research"*)

OR

☐ I authorize the use of my blood samples for doing tests related to this study *BUT* do *NOT* authorize the future use of my blood samples for any other research.

____

Volunteer's signature Date

Investigator's statement (or person performing the Informed Consent procedure)

I certify that I have explained to the above individual the nature and purpose of the study, potential benefits, and reasonably foreseeable risks associated with participation in this research study. I have answered any questions that have been raised and have witnessed the above signature. I have explained the trial, as described in the informed consent form, to the above volunteer on the date stated on this consent form.

____

Last and first name and signature Date
(of the person performing the Informed
Consent procedure)

INFORMED CONSENT FORM
ADDENDUM 1

Safety and Immunogenicity of an Intramuscular, Inactivated, Split-Virion, Pandemic Influenza A/H5N1 Vaccine in Adults and the Elderly (GPA02).

Name and Address of Investigator: [redacted]
Belgium (Center 1).

[redacted]
Belgium. (Center 4).

Name and Address of Sponsor: sanofi pasteur
2, avenue Pont Pasteur
F-69367 Lyon cedex 07
France

This addendum modifies the Informed Consent Form used for this clinical research study in which you are participating, in order to describe the booster vaccination.

Based on the results of the previous study, the Sponsor has now defined the timing of the booster vaccination and the formulations that will be used.

You will receive a booster vaccination of either 30 µg of vaccine with adjuvant or 7.5 µg of vaccine without adjuvant at 12 months following the first vaccination. A new strain of the same (H5N1) vaccine type will be used for the booster vaccination.

As at the beginning of the trial, the formulation that you will be assigned to receive will be decided at random.

The same changes to the study design also apply to the UK study site, and have been made in response to a change in the circulating H5N1 virus. Except for the new virus strain, this new vaccine is made in the same way as the vaccine you received in the first part of the study. Variations in the H5N1 virus have always been expected and mean that we may need to be prepared to immunise against more than one strain in the event of a pandemic.

There are no definite benefits following vaccination in this trial. You may develop an antibody response to this new vaccine strain, although this can not be guaranteed. Furthermore, in the event that an H5N1 strain is found in birds or circulates as a pandemic, cross-protection of such an antibody response cannot be guaranteed.

At the 6 month visit, when a blood sample will be obtained but no vaccine administered, we will discuss these changes with you and answer any questions you may have. You will be asked to

complete the section at the end of this addendum to the Informed Consent Form to confirm that you are happy to continue participating in the study. However, as always, participation is voluntary.

As the booster vaccination is now scheduled for May/June 2007 there is no issue concerning the timing of the winter flu vaccine and the study vaccine. For those of you who are due to receive the winter flu vaccine, please do so as advised by your General Practitioner.

For your information, some changes have been made to the study design by the Sponsor.

- At one other site in Belgium, participants will receive a booster vaccination of 7.5 µg of vaccine without adjuvant at 6 months. The vaccine used in this group will be the same H5N1 vaccine as that used in the first part of the study.
- An additional 100 participants will be recruited into the study at one or more sites in Belgium in May 2007. These participants will receive two doses of the vaccine based on the new H5N1 strain.

If you have any questions about this study or if you want more information on compensation or medical treatment in the case of study injuries, please contact:

Name: ……………….

Tel. Number: …………………..

If you have any questions about your rights as a subject in this study, please contact:

Name: ……………….

Tel. Number: …………………..

Informed consent statement for participating in the study

Subject's Last and First Names: ____

Subject's Study Number: _______________ Subject's Initials: _________

By signing this form I certify that I voluntarily accept to continue participating in the study and to receive the booster vaccination at 12 months after the first vaccination as described in this Addendum 1 to the Informed Consent Form.

____

Subject's signature Date

Investigator's statement (or person performing the Informed Consent procedure)

I certify that I have explained the above modifications to the subject. I have answered any questions that have been raised and have witnessed the above signature.

____

Last and first name and signature Date
(of the person performing the Informed
Consent procedure)

INFORMED CONSENT FORM
ADDENDUM 1

Safety and Immunogenicity of an Intramuscular, Inactivated, Split-Virion, Pandemic Influenza A/H5N1 Vaccine in Adults and the Elderly (GPA02).

Name and Address of Investigator: [redacted] Belgium. (Center 2).

Name and Address of Sponsor: sanofi pasteur
2, avenue Pont Pasteur
F-69367 Lyon cedex 07
France

This addendum modifies the Informed Consent Form used for this clinical research study in which you are participating, in order to describe the booster vaccination.

Based on the results of the previous study, the Sponsor has now defined the timing of the booster vaccination and the formulations that will be used.

You will receive a booster vaccination of 7.5 µg of vaccine without adjuvant at 6 months following the first vaccination. The vaccine strain will be the same as that used in the first two vaccinations.

There are no definite benefits following vaccination in this trial. You may develop an antibody response to this vaccine strain, although this cannot be guaranteed. Furthermore, in the event that an H5N1 strain is found in birds or circulates as a pandemic, cross-protection of such an antibody response cannot be guaranteed.

At the 6 month visit, before blood sampling and vaccination, we will discuss these changes with you and answer any questions you may have. You will be asked to complete the section at the end of this addendum to the Informed Consent Form to confirm that you are happy to continue participating in the study. However, as always, participation is voluntary.

Seasonal flu vaccination could be received at least 4 weeks before this booster vaccination. The Sponsor will provide the vaccine for seasonal flu vaccination.

For your information, some changes have been made to the study design by the Sponsor.

- At the other sites in Belgium and at the site in the UK, subjects will receive a booster vaccination of either 30 µg of vaccine with adjuvant or 7.5 µg of vaccine without adjuvant at 12 months following the first vaccination. A new strain of the same (H5N1) vaccine type will be used for the booster vaccination in May 2007.

- An additional 100 participants will be recruited into the study at one or more site in Belgium in May 2007. These participants will receive two doses of the vaccine based on the new H5N1 strain.

If you have any questions about this study or if you want more information on compensation or medical treatment in the case of study injuries, please contact:

Name: ……………….

Tel. Number: …………………..

If you have any questions about your rights as a subject in this study, please contact:

Name: ……………….

Tel. Number: …………………..

Informed consent statement for participating in the study

Subject's Last and First Names: ____

Subject’s Study Number: _______________ Subject’s Initials: __________

By signing this form I certify that I voluntarily accept to continue participating in the study and to receive the booster vaccination at 6 months after the first vaccination as described in this Addendum 1 to the Informed Consent Form.

____

Subject's signature Date

Investigator's statement (or person performing the Informed Consent procedure)

I certify that I have explained the above modifications to the subject. I have answered any questions that have been raised and have witnessed the above signature.

____

Last and first name and signature Date
(of the person performing the Informed Consent procedure)

INFORMED CONSENT FORM
ADDENDUM 2

Safety and Immunogenicity of an Intramuscular, Inactivated, Split-Virion, Pandemic Influenza A/H5N1 Vaccine in Adults and the Elderly (GPA02).

Name and Address of Investigator:	[redacted]<br>Belgium (Center 1).<br><br>[redacted]<br>Belgium. (Center 4).
Name and Address of Sponsor:	sanofi pasteur<br>2, avenue Pont Pasteur<br>F-69367 Lyon cedex 07<br>France

At the 6-month visit you were informed that the booster vaccination would be performed at 12 months after the first vaccination, and that this would be based on a new strain of the H5N1 (bird flu) virus that is slightly different from that used in the vaccine you have already received.

This Addendum #2 to the Information Sheet is to inform you that, due to the late availability of an important component of the vaccine, the booster vaccination will be delayed. Because of this unplanned delay in the booster vaccination, the study design has been modified so that your next visit will take place at 15 months (rather than 12 months) after the first vaccination, and will include the planned blood test (30 mL) but no booster vaccination. As for the other visits, you will be given a stipend for participation of [redacted].

While it is still intended to administer a booster dose of the new H5N1 vaccine, we are currently not able to indicate exactly when this will be, but we will inform you once this date is known. The visits following the booster vaccination will be maintained at the original timing relative to the booster vaccination, i.e. a visit to provide a blood sample 21 days following the booster and a safety follow-up visit (or telephone call) at 6 months after the booster.

At the 15-month visit, when a blood sample will be obtained but no vaccine administered, we will discuss these changes with you and answer any questions you may have.

The same changes to the study design also apply to the UK study site.

There are no definite benefits following vaccination in this trial. You may develop an antibody response to this new vaccine strain, although this can not be guaranteed. Furthermore, in the event that an H5N1 strain is found in birds or circulates as a pandemic, cross-protection of such an antibody response cannot be guaranteed.

You will be asked to complete the section at the end of this addendum to the Informed Consent Form to confirm that you agree to this additional visit and that you are happy to continue participating in the study. However, as always, participation is voluntary.

If you have any questions about this study or if you want more information on compensation or medical treatment in the case of study injuries, please contact:

Name: ……………….

Tel. Number: …………………..

If you have any questions about your rights as a subject in this study, please contact:

Name: ……………….

Tel. Number: …………………..

Specific consent statement for the future use of your blood sample in research:

Please indicate your choice by checking (ticking) one of the two boxes below:

☐ I authorize the future use of my blood sample for future research by the Sponsor under the conditions described in the original Informed Consent Form (see the paragraph called "*What should you know about possible future use of you blood samples for research"*)

<u>OR</u>

☐ I authorize the use of my blood sample for doing tests related to this study *BUT* do *NOT* authorize the future use of my blood samples for any other research.

____

Volunteer's signature Date

Informed consent statement for continued participation in the study

Subject's Last and First Names: ______________________________

Subject's Study Number: ______________ Subject's Initials: _________

By signing this form I certify that I voluntarily:

☐ accept to participate in the additional visit according to the changes described in this addendum.

☐ accept to continue participating in the study according to the changes described in this addendum.

____

Subject's signature Date

Investigator's statement (or person performing the Informed Consent procedure)

I certify that I have explained the above modifications to the subject. I have answered any questions that have been raised and have witnessed the above signature.

____

Last and first name and signature Date
(of the person performing the Informed Consent procedure)

INFORMED CONSENT FORM
ADDENDUM 3
for subjects having received the 30 µg with adjuvant vaccine for the first and second vaccinations

Safety and Immunogenicity of an Intramuscular, Inactivated, Split-Virion, Pandemic Influenza A/H5N1 Vaccine in Adults and the Elderly (GPA02).

Name and Address of Investigator:	[redacted]<br>Belgium (Center 1).
Name and Address of Sponsor:	sanofi pasteur<br>2, avenue Pont Pasteur<br>F-69367 Lyon cedex 07<br>France

In the Informed Consent Form and the Addendum 2 to the Informed Consent Form, you were informed that you will receive a booster vaccination of either 30 µg of vaccine with adjuvant or 7.5 µg of vaccine without adjuvant, but that the timing of this booster was not decided. A new strain of the same (H5N1) vaccine type will be used for the booster vaccination.

At the 15-month visit you were informed that we will come back to you to let you know when the booster vaccination would be performed.

This Addendum 3 to the Informed Consent Form is to inform you, that the booster will now be at 22 months after the first vaccination and only the 30 µg with adjuvant vaccine will be used for this booster dose. Indeed, results obtained with the 6-month booster with 7.5 µg without adjuvant vaccine showed that this vaccine did not induce a good immune response.

Because of these results and keeping in mind the safety of the participants, it was decided by the Sponsor to adjust the study design as follows:

- All subjects having received the 7.5µg vaccine without adjuvant for the first and second vaccinations will receive no booster and stop the trial at the visit at 22 months. Further studies with this vaccine will not be undertaken. For ethical reasons we do not wish to expose participants to a vaccine that will not be further developed. During the last visit at M22, the Investigator will interview the participant on any important medical events which may have occurred since the last visit.
- As a smaller subset of subject is sufficient to analyze the booster vaccination response, a subset of subjects having received the 30µg with adjuvant vaccine for the first and second vaccinations will receive no booster and stop the trial at the visit at 22 months.

- In your center all subjects having received the 30µg with adjuvant vaccine for the first and second vaccinations will receive their booster with the 30µg with adjuvant vaccine at the 22-month visit.

All the changes have been approved by the Ethics Committee.

During the booster visit at M22, a 30 mL blood sample will be taken to assess the presence of antibodies before performing the booster vaccination. As for the first and second vaccinations, to ensure your safety, you will stay at the study center under medical observation for 30 minutes following vaccination. You will then be given a third diary card to note any medications that you may take and unwanted effects that may happen between the second vaccination and the next visit.

In order to better assess the booster effect on your immune response, an additional visit will take place 7 days after the booster (V05_Add), during which a 30 mL blood sample will be taken to assess how quickly your immune system responds to the vaccine.

The study will last a total of 28 months.

At the 22-month visit, we will discuss these changes with you and answer any questions you may have.

The same changes to the study design also apply to the UK study site, whereas in the study center of [redacted] the participation of all subjects will stop at M22.

There are no definite benefits following vaccination in this trial. You may develop an antibody response to this new vaccine strain, although this can not be guaranteed. Furthermore, in the event that an H5N1 strain is found in birds or circulates as a pandemic, cross-protection of such an antibody response cannot be guaranteed.

As for the other visits, you will be given a stipend for participation of [redacted]

You will be asked to complete the section at the end of this addendum to the Informed Consent Form to confirm that you agree to this additional visit and that you are happy to continue participating in the study. However, as always, participation is voluntary.

If you have any questions about this study or if you want more information on compensation or medical treatment in the case of study injuries, please contact:

Name: … [redacted] …………….

Tel. Number: [redacted]

If you have any questions about your rights as a subject in this study, please contact:

Name: [redacted]

Tel. Number: [redacted]

Specific consent statement for the future use of your blood sample in research:

Please indicate your choice by checking (ticking) one of the two boxes below:

☐ I authorize the future use of my blood sample for future research by the Sponsor under the conditions described in the original Informed Consent Form (see the paragraph called "*What should you know about possible future use of you blood samples for research"*)

<u>OR</u>

☐ I authorize the use of my blood sample for doing tests related to this study *BUT* do *NOT* authorize the future use of my blood samples for any other research.

____

Volunteer's signature Date

Informed consent statement for continued participation in the study

Subject's Last and First Names: ______________________________________

Subject's Study Number: ____________ Subject's Initials: ________

By signing this form I certify that I voluntarily:

☐ accept to continue participating in the study according to the changes described in this addendum.

____

Subject's signature Date

Investigator's statement (or person performing the Informed Consent procedure)

I certify that I have explained the above modifications to the subject. I have answered any questions that have been raised and have witnessed the above signature.

____

Last and first name and signature Date
(of the person performing the Informed
Consent procedure)

INFORMED CONSENT FORM
Additional subjects

Safety and Immunogenicity of an Intramuscular, Inactivated, Split-Virion, Pandemic Influenza A/H5N1 Vaccine in Adults and the Elderly (GPA02).

Name and Address of Investigator: [redacted] Belgium. (Center 2).

Name and Address of Sponsor: sanofi pasteur
2, avenue Pont Pasteur
F-69367 Lyon cedex 07
France

You are being asked to volunteer to take part in a research study to help to develop a vaccine against a pandemic influenza virus, which is being conducted in the UK and Belgium since May 2006. Although this vaccine has been approved for use in this particular study, it has not been licensed for use in the UK or Belgium by the Medicines and Health-Care Products Regulatory Agency (MHRA) or the Federal Agency for Medicines and Health Products (AFMPS/FAGG). No guarantee or assurance can be made regarding the results of this study.

This study is being paid for by sanofi pasteur (the rest of this form will refer to sanofi pasteur as "the Sponsor"). [redacted] the "Investigator" for the study, and his institution will receive a research grant for the conduct of this study. The Ethics Committees (EC)/Institutional Review Boards (IRB) of your center approved this study.

This document will provide you with the information needed to help you decide whether you wish to take part in this study. If any part or word of this document is unclear, or if you have any questions or want additional information at any time, please do not hesitate to ask one of the study team members.

What is Pandemic Influenza?

Since December 2003, a growing number of Asian and Eastern European countries have reported outbreaks of highly pathogenic avian influenza in chickens and ducks, resulting in the death or culling of more than 100 million poultry. A highly pathogenic strain, A/H5N1, has been the cause of most of these outbreaks.

Between December 2003 and mid-November 2007, A/H5N1 viruses have caused at least 335 confirmed cases (including 206 deaths) in human adults and children in Azerbaijan, Cambodia, China, Djibouti, Egypt, Indonesia, Iraq, Lao People's Democratic Republic, Nigeria, Thailand, Turkey, and Vietnam. To date, people who have been infected with the A/H5N1 virus

have been in direct contact with infected and sick birds. The virus has not been infectious between humans.

There is a concern that the A/H5N1 virus, if given sufficient opportunity, will mutate into a form that is highly infectious and for which humans would have no natural immunity (that is, the population would have no antibody in the blood to protect against the virus). Such a virus could spread easily from one person to another, resulting in pandemic influenza.

Past influenza pandemics have been characterized by the sudden onset of severe typical influenza symptoms such as headache, high fever, muscle and joint aches, reduced appetite, nausea, vomiting and cough, which typically last for 2 to 4 days. Some patients, after an initial recovery, subsequently develop pneumonia, and although most patients ultimately recover, some die rapidly due to tracheo-bronchitis associated with breathing difficulties.

What vaccines or other treatments may be used to prevent or treat Pandemic Influenza?

In the event of a pandemic, it will be possible to use antiviral drugs, which are commonly used to treat influenza each year (for more information, ask your doctor), to control pandemic influenza and to treat those infected with the virus however the effectiveness of anti-viral drugs in the treatment of pandemic influenza has yet to be demonstrated. Vaccination may be effective at preventing pandemic influenza but as with antiviral drugs the use of an A/H5N1 vaccine in an A/H5N1 pandemic has to date not been demonstrated.

What is the purpose of this study?

This is a medical research study, the purpose of which is to test two influenza pandemic vaccine formulations as a two-primary dose schedule plus a booster vaccination in subjects aged either 18 to 60 years, or >60 years. The influenza pandemic vaccine is produced from an avian influenza strain, specifically an A/H5N1 virus (“H” and “N” stand for two proteins present on the surface of the virus and these two proteins are responsible for the virulence of the virus).

A similar influenza vaccine has already been injected in 300 adults (aged 18 to 40 years) in a study conducted in France. This study tested several adjuvanted and non-adjuvanted A/H5N1 vaccine formulations (from 7.5 µg to30 µg) given as a two-dose schedule. An adjuvant is a component of many vaccines, which is added to enhance the immune response. Each formulation of the vaccine appeared to be generally safe and well tolerated. The highest antibody (substances that protect the body from infections) response was observed following vaccination with the 30 µg dose with adjuvant, and responses were also observed following the other doses/formulations, especially following 7.5 µg without adjuvant.

Based on these results, the 30 µg of vaccine with adjuvant and 7.5 µg of vaccine without adjuvant formulations were selected for use in the first part of the present trial. The objectives of the present study are to describe the safety and the immune response following administration of two vaccinations (separated by 21 days) followed by a booster vaccination. The booster dose will be administered 6 or 22 months after the first vaccination. Subjects have already been recruited in the study for the assessment of these objectives.

Due to the circulation of new A/H5N1 virus strain in birds and in isolated human cases, it has been decided to perform the booster at 22 months with a new vaccine strain derived from the new virus strain.

Additional participants will be recruited and will receive two vaccinations of the 30 µg vaccine with adjuvant of this new strain 21 days apart, in parallel to the booster vaccination at 22 months, in order to assess the effect of this strain in subjects never exposed to the virus or to a vaccine. You will be part of these additional participants.

How will this study be done?

For you, this study will consist of four visits (although the final 'visit' may be conducted by telephone) and will last 6 months. It will involve 50 adults aged 18 to 60 years in Belgium.

You will receive two injections of the 30 µg of vaccine with adjuvant, separated by an interval of 21 days.

Should you decide to volunteer for the study, you will be asked to come to the study center for three visits, and to record any unwanted medical effects that could occur between these visits. At each visit, the Investigator will examine you and will ask you questions about your health. In addition, a blood sample (30 mL) will be taken from each subject at each visit to measure the amount of antibodies.

- First visit:

All the risks and benefits associated with the study will be explained to you before signing the following informed consent statement. After having received satisfactory answers to all your questions, and if you agree to take part in the study, you will sign the informed consent form.

The Investigator will examine you and check your medical history to make sure that you can take part in the study. This involves a physical examination, a urine pregnancy test if you are a woman of child-bearing potential, and recording of your medical history (especially regarding any allergic reactions you may have had to previous vaccinations or any allergy to egg proteins). You will be asked not to participate in another research study for the whole period of the present study.

If you are eligible for the study, 30 mL of blood will be taken in order to measure your level of antibodies before vaccination. You will then receive an injection of the vaccine into your upper-arm muscle. To ensure your safety, you will stay at the study center under medical observation for 30 minutes following vaccination.

You will then be given a digital thermometer, a ruler, and a diary card to note any unwanted effects that may happen between vaccination and the next visit, and you will be instructed on how to use them. On the evening of vaccination and on each of the next 7 days, you will be asked to record your oral temperature and any specified symptoms commonly observed after any vaccine administration. You will also be asked to record any medications (other than the vaccine) that you may take during the 21 days following the vaccination. In addition, any non-specified symptoms occurring between the vaccination and the second visit should be recorded in the diary card.

- Second visit: 21 days after the first vaccination:

The Investigator will examine you, review with you the diary card that you will have completed since the first vaccination and will question you on your health since vaccination. A urine pregnancy test will be performed if you are a woman of child-bearing potential. A 30 mL blood sample will be taken to test the effect of the vaccine on your body's immune system. Then you will receive the second vaccine dose into your upper-arm muscle. To ensure your safety, you will stay at the study center under medical observation for 30 minutes following vaccination.

You will then be given a second diary card to note any medications that you may take and unwanted effects that may happen between the second vaccination and the next visit.

- Third visit: 21 days after the second vaccination:

The Investigator will examine you, review with you the diary card that you will have completed since the previous visit and will question you on your health since the previous visit. A 30 mL blood sample will be taken in order to further investigate the effect of the vaccine on your body's immune system.

A memory aid will be provided to help you to record any important events until the next visit.

- Fourth visit (or telephone call): 6 months after the second vaccination:

The Investigator will examine you (or interview you over the telephone), review with you the memory aid that you will have completed since the previous visit and will question you on your health since the previous visit. This visit will mark the end of your participation in the study.

What are the risks and possible unwanted effects of vaccination and blood sampling?

Influenza vaccines are usually well tolerated when injected into a muscle. However, as with the administration of any other vaccine, you may experience a variety of unwanted events at the injection site, such as pain, redness, hardness, swelling, or bruising, which usually occur within 3 days after vaccination and resolve spontaneously within 3 days. You may also feel unwell or experience headache, muscle and joint pain, high temperature (39°C to 40°C), or malaise. These effects usually disappear without treatment within 3 days. This is not a complete list of possible effects.

Since influenza vaccines have been marketed, a few other reactions like itching, urticaria, or redness on the entire body have been observed infrequently (i.e., in less than 1 out of 100 persons and more than 1 out of 1,000). Nerve pain, paresthesia (feeling of “pins and needles”), convulsions, and temporary low blood platelet count have occurred rarely (i.e., in less than 1 out of 1,000 persons and more than 1 out 10,000). Very rare reactions like neurological disorders have been reported (in less than 1 out of 10,000 persons). As with any vaccination, there is a rare possibility of an allergic reaction. This may cause a severe narrowing of the air passages and breathing difficulties. If this occurs, you will need immediate medical attention. This is why you are requested to remain under medical observation in the study center for 30 minutes after each vaccination.

In addition, the sampling of blood from a vein may cause some pain, bruising at the puncture site, or lightheadedness.

There may be also other risks for you or, if you are a woman, for your unborn child, should you become pregnant during the study, that are not yet known. If you are pregnant at the moment, inform the Investigator. If you become pregnant during the study, the Investigator will withdraw you from the study but will follow you until the end of your pregnancy to monitor your health and that of your baby.

What are the possible benefits for you?

The efficacy of the vaccine has not yet been established and there is no certainty that the influenza strain used in this vaccine will induce a pandemic. As such, no benefit can be guaranteed.

What will you have to do?

If you agree to take part in this study, you will have to attend the three planned visits. As described above, after each vaccination, you will be given a diary card to note any medical events or symptoms that may appear after vaccination until the following visit, as well as any medication taken during that time. You will also have to measure your temperature orally in the evening following each vaccination and for the seven subsequent days, and each time you feel feverish.

You or a family member must inform the Investigator as soon as possible if a serious medical event occurs, e.g., if you are admitted to hospital or visit an emergency department.

Women will have to use an effective method of birth control for at least 4 weeks prior to and 4 weeks after each vaccination. You will have to inform the investigator as soon as possible should you become pregnant. If you plan to become pregnant during the next 6 months, you should not participate in the study.

Who will see your medical and personal information?

Personal information collected during the study will be confidential (with the exception of the situation described in the paragraph below); this confidential information will be computerized and sent to the Sponsor, and, if necessary, to authorized regulatory authorities. The personal information that will be collected includes medical data, date of birth. You may exercise your right to access and correct your data. In these files, your name will be coded using your initials and a study number. This is to protect your privacy. Your name and other information identifying you will not be forwarded to the Sponsor.

In addition to the use of computerized information as described above, any personal records, including your medical records and health history, relating to this study may be inspected in the Investigator's offices by authorized representatives of the Sponsor, or the health authorities in any country where the study vaccine may be considered for approval. Absolute confidentiality of your records can therefore not be guaranteed.

The results of this study may be presented at meetings or in publications. Your identity will not be revealed in these presentations.

What happens if you refuse to take part in the study or change your mind after you agree?

Your participation in this study is entirely voluntary. If you do not want to take part then you do not have to. Also, if you agree to take part but change your mind, you have the right to do so. You can withdraw from the study at any time without any liability for you. If you withdraw from the study, the blood samples collected before your withdrawal will be used unless if you specifically request otherwise.

Whatever your decision, you will continue to qualify for full medical care without any penalty or loss of benefits to which you are otherwise entitled.

The Investigator or Sponsor may withdraw you from this study for any reason at any time even without your consent, for example if the Investigator decides that it is in your best interest, or if you do not follow the study instructions.

If you do decide to withdraw from the study, or if you are withdrawn by the Investigator, then the Investigator may recommend that certain laboratory tests or physical examinations should be done to ensure your safety and well-being.

What else do you need to know before agreeing to take part in this study?

Taking part in this research is entirely voluntary. You will be provided with any new relevant information during the study that may affect your willingness to continue taking part.

This study has been designed and will be carried out in accordance with the ICH/GCP guidelines laid down in the Declaration of Helsinki for the protection of individuals participating in clinical trials.

You will receive a copy of this signed Informed Consent Form. Please keep it safe and use it for information and reference throughout the trial.

In accordance with the law of 07 May 2004 regarding experiments involving human subjects, the Sponsor, even if faultless, is liable for any damages suffered by the participants which are directly or indirectly related to the study. Insurance has been taken out for this purpose.

You will be given a stipend for participation of [redacted] per trial visit.

Do not sign this Informed Consent Form or participate in this study unless you are comfortable with the risks involved and you have had an opportunity to ask questions and feel you have received satisfactory answers.

What will happen to your blood samples?

As a part of this study, blood samples will be taken to test the effect of the A/H5N1 vaccine on your body's immune system. These tests will be done by the Sponsor or by another laboratory for the Sponsor. Your samples will be shipped out of your country for these tests and will be stored in a freezer until these tests are done. Therefore, you should only agree to take part in this study if you agree to this use of your blood samples.

Any unused part of your blood samples will be securely stored by the Sponsor in the USA. They will be stored until at least 10 years after the vaccine being tested has been approved for use by the health authorities.

What should you know about possible future use of your samples for research?

The Sponsor would also like to keep any unused part of your blood samples for use in other evaluation studies, e.g. studies of the immune response to influenza vaccines, improvement of knowledge and documentation of the safety of this vaccine by using new developed laboratory methods or applying newly discovered concepts. You will not be informed about the types of research that will be conducted or any of the results, although, genetic tests will never be performed on these samples.

You have the right to agree to take part in the study proposed to you today and still refuse to allow the Sponsor keep any unused blood samples for future use. You will be asked to decide whether or not you permit this future use of your blood samples and to indicate your decision on the consent form.

You will not be paid for allowing the Sponsor to keep and later use any remaining part of your blood samples.

Should you refuse to allow the Sponsor to keep your unused blood samples then they will not be used for any testing other than that directly related to this study.

Who can you contact if you have questions?

If you have any questions about this study or if you want more information on compensation or medical treatment in the case of study injuries, please contact:

Name: ………[redacted] ……….

Tel. Number: [redacted]

If you have any questions about your rights as a subject in this study, please contact:

Name: ……[redacted] ………….

Tel. Number: [redacted]

Informed consent statement for participating in the study

Subject's Last and First Names: ____________________________

Subject’s Study Number: __[redacted]___________ Subject’s Initials: _________

By signing this form I certify to all of the following:

- I have read this entire Informed Consent Form (or had the information read to me) and understand what will be done to me and what I am being asked to do.
- I consent to take part in this study.
- I consent to make confidential personal information available for review (direct access) to the Sponsor's representative or to any Competent Authorities, Institutions or governmental agencies assigned this task in this country or in another country where the study vaccine may be considered for approval, or, if applicable, the IRB Ethics Committee.
- I understand and accept that information related to me, including information on my ethnic origin, collected during the study will be coded so that my name does not appear in my record, and that this information is computerized and shared with the Sponsor. I am aware that my computerized information may be handled in another country. I am aware that I may exercise my right of access and correction of these data at any time with the investigator.
- I have had the opportunity to ask questions and I understand that I will receive a signed copy of this Informed Consent Form.
- I understand that I may ask additional questions about this study at any time.
- I understand that I am free to withdraw from the study at any time without justifying my decision to do so and without it affecting my medical care.
- I understand that if I withdraw my consent, blood samples collected before my withdrawal will be used unless I specifically request otherwise.
- I authorize the shipment of my blood samples to the Sponsor’s laboratory (or other location as applicable) for doing tests related to this study, and I understand that the laboratory may be in another country.

Specific consent statement for the future use of your blood samples in research:

Please indicate your choice by checking (ticking) one of the two boxes below:

☐ I authorize the future use of my blood samples for future research by the Sponsor under the conditions described in this information sheet (see the paragraph called "*What should you know about possible future use of you blood samples for research"*)

OR

☐ I authorize the use of my blood samples for doing tests related to this study *BUT* do *NOT* authorize the future use of my blood samples for any other research.

____

Volunteer's signature Date

Investigator's statement (or person performing the Informed Consent procedure)

I certify that I have explained to the above individual the nature and purpose of the study, potential benefits, and reasonably foreseeable risks associated with participation in this research study. I have answered any questions that have been raised and have witnessed the above signature. I have explained the trial, as described in the informed consent form, to the above volunteer on the date stated on this consent form.

____

Last and first name and signature Date
(of the person performing the Informed
Consent procedure)

Protocol Appendix 3: Signature Page
