# Supplementary material for: Antibody Persistence and Booster Responses to Split-Virion H5N1 Avian Influenza Vaccine in Young and Elderly Adults
Source: PLoS One. 2016 Nov 4;11(11):e0165384. doi: 10.1371/journal.pone.0165384 (PMC5096706; doi:10.1371/journal.pone.0165384)
Supplement: S1 Table — (DOCX) [file pone.0165384.s004.docx]

**S1 Table : Baseline Characteristics of Groups at time of first vaccination**

|  | Booster Vaccination group Age: 18 to 60 | Booster Vaccination group Age: > 60 | Primary Vaccination group |
| --- | --- | --- | --- |
| Age Mean ± SD (years) | 36.6 ± 13.4 | 67.3 ± 5.1 | 30.2 ± 13.5 |
| Male:Female | 0.9 | 1.3 | 1.3 |
| Weight Mean ± SD (kg) | 74.7 ± 16.4 | 76.9 ± 13.4 | 69.6 ± 11.8 |
